# Study on the Relationship of Ions (Na, K, Ca) Absorption and Distribution to Photosynthetic Response of *Salix matsudana* Koidz Under Salt Stress

**DOI:** 10.3389/fpls.2022.860111

**Published:** 2022-05-03

**Authors:** Xin Ran, Xiao Wang, Xiaoxi Huang, Changming Ma, Haiyong Liang, Bingxiang Liu

**Affiliations:** ^1^Department of Forest Cultivation, College of Forestry, Hebei Agricultural University, Baoding, China; ^2^Hebei Urban Forest Health Technology Innovation Center, Baoding, China

**Keywords:** chlorophyll content, fluorescence parameters, ion absorption and distribution, photosynthetic response, salt stress

## Abstract

To identify the key indicators for salt tolerance evaluation of *Salix matsudana* Koidz, we explored the relationship of ion absorption and distribution with chlorophyll, fluorescence parameters (leaf performance index, maximum photochemical efficiency), and photosynthetic gas parameters (net photosynthetic rate, transpiration, stomatal conductance, intercellular carbon dioxide concentration) under salt stress. We established 4 treatment groups and one control group based on salinity levels of NaCl hydroponic solutions (171, 342, 513, and 684 mm). The Na^+^/K^+^, Na^+^/Ca^2+^, chlorophyll fluorescence, and photosynthetic parameters of leaves were measured on the 1st, 3rd, 5th, 8th, 11th, and 15th days to analyze the correlations of chlorophyll, chlorophyll fluorescence and photosynthetic parameters to the ion distribution ratio. The results showed that (1) The ratio of the dry weight of roots to leaves gradually increased with increasing salt concentration, whereas the water content of leaves and roots first increased and then decreased with increasing time. (2) The content of Na^+^, Na^+/^K^+^, and Na^+^/Ca^2+^ in roots and leaves increased with increasing salt stress concentration and treatment time, and the difference gradually narrowed. (3) Ca^2+^ was lost more than K^+^ under salt stress, and Na^+^/Ca^2+^ was more sensitive to the salt stress environment than Na^+^/K^+^. (4) Because the root system had a retention effect, both Na^+^/K^+^ and Na^+^/Ca^2+^ in roots under different NaCl concentrations and different treatment times were higher than those in leaves, and Na^+^/Ca^2+^ was much higher than Na^+^/K^+^ in roots. (5) Na^+/^K^+^ had a higher correlation with fluorescence parameters than Na^+^/Ca^2+^. Among them, Na^+^/K^+^ had a significantly negative correlation with the maximum photochemical efficiency, and the correlation coefficient *R*^2^ was 0.8576. (6) Photosynthetic gas parameters had a higher correlation with Na^+^/Ca^2+^ than with Na^+^/K^+^. Among them, significantly negative correlations were noted between Na^+^/Ca^2+^ and Gs as well as between Na^+^/Ca^2+^ and E under salt stress. The correlation between Na^+^/Ca^2+^ and Gs was the highest with a correlation coefficient of 0.9368. (7) Na^+^/K^+^ and Na^+^/Ca^2+^ had no significant correlation with chlorophylls. Na^+^/Ca^2+^ was selected as a key index to evaluate the salt tolerance of *S. matsudana* Koidz, and the results provided a reference for analyzing the relationship between ion transport and distribution for photosynthesis.

## Introduction

Soil salinization is one of the most severe environmental problems facing the world (Fang et al., 2021). It not only influences the normal growth and development of plants but also restricts the increase in yield and quality of agricultural products. There are approximately 2.6 million square kilometers of saline-alkali lands in China, which is equivalent to 1/4 of the country’s total national territorial area (Guan et al., 2010). These lands are mainly extensively distributed in coastal and arid areas (Lu et al., 2021). The assessment of the salt tolerance of plants plays a crucial role in improving the application of plants in saline-alkali areas. At present, scholars’ evaluation of plant salt tolerance mainly focuses on growth and development indicators, photosynthetic pigments, photosynthetic system characteristics, ion absorption, ion distribution and transportation indicators, and chlorophyll fluorescence parameters. Their research objects mainly cover some halophytes (Liu W. C. et al., 2013; Sun L. et al., 2021) and crops (Xu Y. et al., 2020; Dou et al., 2021), but few studies have focused on forest trees.

Most of the plants in the family Salicaceae resist salinization. *Salix matsudana* Koidz, an important economic and green tree species belonging to the family Salicaceae, features fast growth, waterlogging tolerance (Wang C. Y. et al., 2017) and easy propagation. It can grow well under saline-alkali, drought, waterlogging, and poor soil conditions. It also has the characteristics of wind prevention and sand fixation, so it is widely used for urban landscaping. *S. matsudana* Koidz has the strong ecological ability and mainly propagates through cottage with a very high survival rate (Liu et al., 2018), so it is difficult to be replaced by other tree species. For the above reasons, willows have received extensive attention from experts and scholars, and related research is being constantly deepened. At present, research on *S. matsudana* Koidz mainly focuses on germplasm resource screening (Yu et al., 2018), cultivation management (Ma et al., 2021), stress-resistant physiology, and genetic diversity (Zhang J. et al., 2021). Regarding stress resistance, studies have mainly focused on physiological characteristics under the conditions of plant diseases and insect pests (Zhang M. Y. et al., 2021), heavy metals (Yuan et al., 2019), moisture (Jiménez-Rodríguez et al., 2019), and salinity (Kumari et al., 2021). However, studies on the relationship of ion absorption and distribution to photosynthesis of roots and leaves under salt stress are rarely reported. It remains to be clarified whether the salt ion content and its ratio in *S. matsudana* leaves are the main factors affecting photosynthesis. What are the effects of sodium ions, sodium-potassium ratios, and sodium-to-calcium ratios, which are widely used in current research to evaluate plant salt tolerance, on the photosynthetic system? Which indicator has a greater impact on the photosynthetic system? Which index is more suitable for evaluating the effect of leaf ion distribution on the photosynthetic system? To clarify these problems, this study used *S. matsudana* as a representative plant to analyze the correlation between leaf salt ion content and its ratio and photosynthetic system to provide a theoretical reference for evaluating the salt tolerance of plants in saline-alkali areas.

## Materials and Methods

### Plant Material and Salt Treatments

The test materials were collected from the germplasm resource nursery of Jinshatan Forest Farm in Huai’an County, Hebei Province. In 2020, annotinous branches of *S. matsudana* Koidz, which basically maintained the same strong growth vigor and were free of diseases and insect pests, were collected before germination in early spring and stored in the freezer at 0°C. On 26 June 2020, a water culture experiment was conducted in the artificial climate chamber of the west campus of Hebei Agricultural University (38°48′N, 115°25′E) of Baoding, Hebei Province. The temperature of the climate chamber was set to 28/25°C (light/dark), the LED cold light source maintained the light intensity at 1,000 μmol⋅m^–2^⋅ ^–1^, the photoperiod was 14/10 h (light/dark) and the relative humidity was 60%. Some studies have shown that PAR = 1,000 μmol⋅m^–2^⋅^–1^ is beneficial to determine the best photosynthetic system parameters (Liu T. et al., 2013; Zhu Y. X. et al., 2019). In addition, some scholars also set the PAR at 800–1,000 μmol⋅;m^–2^⋅s^–1^ and the RH at 50–70% when setting up the experiment (Gerona et al., 2019; Zhu H. L. et al., 2019; Zhang et al., 2020; Liu et al., 2022; Ma et al., 2022). Therefore, we set the light intensity and relative humidity according to the previous research and the actual situation. The middle two-thirds of the selected branches were cut into 20 cm long cuttings. The uppermost bud was 0.5–1 cm from the top of the cuttings. The uppercut was a flat cut, and the lower cut was an oblique cut. The selection of branches refers to the method of Ran et al. (2021). On 15 July, when their growth reached the treatment requirement (the average root length was approximately 5 cm), seedlings with uniform growth potential and root systems were selected for stress treatment.

The test material was placed in a 55 cm × 38 cm × 15 cm (length × width × height) plastic box for hydroponic culture. When most scholars study the effects of salt stress on plants under hydroponic conditions, the salt concentration is mostly set between 100 and 700 mmol. We also converted the corresponding concentration into international units. For these reasons, we decided to adopt such a salt concentration (Miranda-Apodaca et al., 2020; Roman et al., 2020; Gao et al., 2022; Gou et al., 2022; Ni et al., 2022). A 1/2 dilution of Hoagland’s complete nutrient solution was used as the base to prepare hydroponic solutions with NaCl concentrations of 171, 342, 513, and 684 mmol, and 1/2 Hoagland’s complete nutrient solution (pH = 7.2) was used as a control (CK). In brief, 1/2 Hoagland’s complete nutrient solution includes: 30 mg NaFeC1_0_H_12_N_2_O_8_⋅3H_2_O, 472.5 mg Ca(NO_3_)_2_⋅_2_O, 15 mg FeSO_4_, 303.5 mg K_2_SO_4_, 0.05 mg CuSO_4_, 57.5 mg NH_4_H_2_PO_4_, 2.13 mg MnSO_4_, 246.5 mg MgSO_4_, 2.86 mg H_3_BO_3_, 4.5 mg Na_2_B_4_O_7_⋅10H_2_O, 0.22 mg ZnSO_4_, and 0.02 mg H_8_MoN_2_O_4_.

During the experiment, each treatment was repeated thrice, and 10 seedlings were directly placed in the solution with more than half of the height covered in the prepared solution. We changed the nutrient solution every 5 days during their growth. To prevent excessive accumulation of salt, we removed the seedlings and carefully rinsed off the residual salt from the roots with clean water before changing the nutrient solution. We randomly selected 3 seedlings with average growth vigor on the 1st, 3rd, 5th, 8th, 11th, and 15th days of the stress test for the determination of various growth physiological indices.

### Growth Indicators

The ratio of the dry weight of roots and leaves and the water content of leaves and roots were determined by Li (2000). We took 3 weighing bottles (repeated thrice using the same procedure as described below), numbered them in turn and accurately weighed them. We selected the sample to be measured, immediately placed it into the above weighing bottle, closed the bottle cap tightly, and accurately weighed it. The weighing bottle was placed in an oven at 105°C for 15 min to kill the plant tissue cells and then baked at 80–90°C to a constant weight (it must be placed in a desiccator when weighing and weighed after cooling). Let the weight of the weighing bottle be W1, the weight of the weighing bottle and the sample be W2, and the weight of the weighing bottle and the dried sample be W3 (the above weight units are all g as described below). Then, the total water content of the plant tissue (%) can be calculated as follows. The value of the total water content of the plant tissue obtained by three repetitions was obtained, and the average value was further obtained.


(1) W=[(W2-W3)/(W2-W1)]/3*100%

(2) Rootwatercontent:Wroot=[(W2root-W3root)/(W2root-W1)]/3*100%

(3) Leavewatercontent:Wleave=[(W2leave-W3leave)/(W2leave-W1)]/3*100%

(4) Root⁢dry⁢weirht:WDroot=(W3⁢root-W2⁢root)/3

(5) The⁢leaf⁢dry⁢weight:WDleave=(W3⁢leave-W2⁢leave)/3

(6) The⁢root⁢dry⁢weight/the⁢leaf⁢dry⁢weight=WDroot/WDleave

### Ion Content, Absorption, and Transport in Roots and Leaves

The determination of ion content was performed according to the methods of Yang (2010) and Meng et al. (2020). The sample was first baked at 105°C for 30 min and then dried at 70–80°C to a constant weight. After it was ground into powder, the fixed mass was weighed. After 30 ml of deionized water was added, the sample was shaken well and placed in a boiling water bath for 2 h. After cooling, the sample was filtered and diluted to 50 ml. The Na^+^, K^+^, and Ca^2+^ contents were determined by the atomic absorption method. Zeenit 700P instrument of Analytik Jena Company in Germany was used for determination. The selective absorption and transport coefficients of X (K^+^ and Ca^2+^) ions in roots and leaves were calculated by referring to the methods of Yang et al. (2003) and Zhang et al. (2018). The following formula was used to calculate the selective absorption and transport coefficients of ions X (K^+^ and Ca^2+^) by roots and leaves, where ion absorption coefficient Eq. 7 and ion transport coefficient Eq. 8. In the formula, the K^+^ content was 272 mg, and the Ca^2+^ content was 230 mg in the medium (culture broth).


(7) $${\text{SA}}_{X,\mathrm{Na}}=\mathrm{Root}\left(\left[X\right]/{\text{[Na}}^{+}\right])/\mathrm{Medium}\left(\left[X\right]/{\text{[Na}}^{+}\right])$$

(8) $${\text{ST}}_{X,\mathrm{Na}}=\mathrm{Leaf}\left(\left[X\right]/{\text{[Na}}^{+}\right])/\mathrm{Root}(\left[X\right]/\left[{\mathrm{Na}}^{+}\right])$$

### Measurements of Chlorophyll Content, Chlorophyll Fluorescence Parameters and Photosynthetic Parameters

Chlorophyll content was determined using the method proposed by Li (2000). The measurement of chlorophyll fluorescence parameters adopted the method of Ran et al. (2021). After the beginning of the stress test, three seedlings with average growth vigor were selected for each treatment to the determination of fluorescence parameters. Before the determination, leaves were subjected to dark adaptation for 15 min, and then the rapid chlorophyll fluorescence induction kinetic curve and related parameters were measured using a Pocket PEA plant efficiency analyzer (Hansatech, United Kingdom). The obtained OJIP curve was used to analyze the fast chlorophyll fluorescence induction curve data (Wang et al., 2004; Giorio and Sellami, 2021; Stefanov et al., 2021) (JIP-test), and related parameters were calculated. The initial fluorescence (Fo), maximum fluorescence (Fm), maximum photochemical efficiency (Fv/Fm) after dark adaptation, and performance index (PI_*ABS*_) based on absorbed light energy were directly determined for leaves.

The photosynthetic parameters adopted the method of Ran et al. (2021). During the stress treatment, 3 seedlings with average growth vigor were selected for each treatment. After the 3 seedlings were subjected to normal lighting in the climate chamber for 3 h, the 3rd–5th fully expanded leaves of the same position, size, and light receiving direction were selected from top to bottom to determine photosynthetic gas exchange parameters, such as stomatal conductance (Gs), intercellular CO_2_ concentration (Ci), transpiration rate € and net photosynthetic rate (Pn), with the aid of a Li-6800 portable photosynthesizer (LI-COR of United States).

### Experimental Design and Statistical Analysis

Microsoft Excel and Origin were used to fit the response curve data. The test data were calculated, processed, and plotted using Excel 2020 for the correlation evaluation and analysis of the ion absorption, distribution, and photosynthetic indices of *S. matsudana* Koidz seedlings. SPSS 18.0 data processing software was used for statistical analysis and the significance of the difference at the *p* < 0.05 level was tested based on the least significant difference (LSD).

## Results

### Analysis of the Dry Weight Ratio and Water Content of Roots and Leaves Under Salt Stress

**FIGURE 1:**
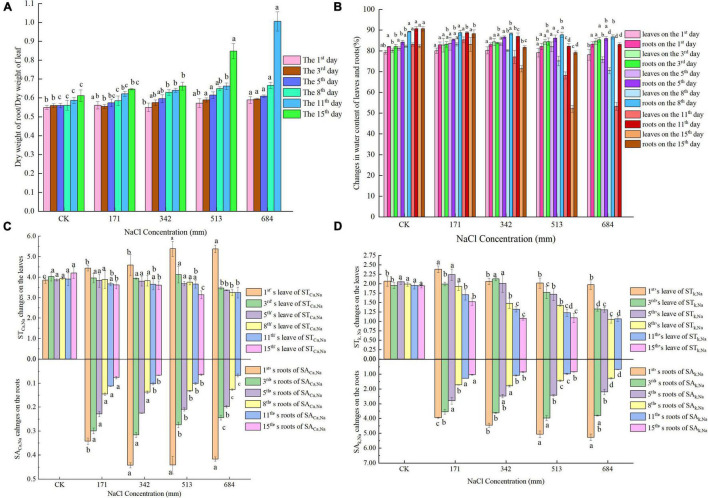
Effects of salt stress on ratio of dry weight and water content of roots and leaves, ratio of selective absorption and transport of Na^+^/ K^+^ and Na^+^/ Ca^2+^ in roots and leaves; **(A)** effects of salt stress on ratio of roots and leaves dry weight of *S. matsudana*; **(B)** effects of salt stress on water content of roots and leaves of *S. matsulosa* **(C)** effects of salt stress on selective uptake and transportation of Ca^2+^ in roots and leaves of *S. matsudana*; **(D)** effects of salt stress on selective uptake and transportation of K^+^ in roots and leaves of *S. matsudana*. Data shown are means ± SEM. Different small letters indicate significant differences between treatments at 0.05 level among treatment.

Under different concentrations of salt stress and different action times, the dry weight ratio of dry willow seedling roots to leaves and the degree of change in water content were different (Figures 1A,B). Intensified salt stress led to a gradual increase in the ratio of root to leaf dry weight. On the 1st day of stress, the water content of *S. matsudana* leaves was not significantly affected by salt stress; on the 3rd day, the water content of the leaves showed an increasing trend with increasing salt concentration. On the 5th day, the root water content showed an overall upward trend. Then, the root water content gradually decreased with increasing stress time and further aggravation of the stress.

### Analysis of Selective Absorption and Transport Proportions of Na^+^/K^+^ and Na^+^/Ca^2+^ in Roots and Leaves Under Salt Stress

Figures 1C,D shows that under stress by different concentrations of NaCl, the SA _k, Na_ values of *S. matsudana* Koidz roots showed an overall downward trend as the treatment time increased. On the 1st day, the SA _k, Na_ values of *S. matsudana* Koidz roots showed an overall upward trend with increasing NaCl concentration. After 3 days of stress, at each stress time, SA _k, Na_ gradually decreased with increasing stress, and the difference between treatments was significant. In the first 3 days of stress, the SA _*Ca*, Na_ values of roots showed a decreasing trend after increasing as the salt concentration increased. On the first day, the SA _*Ca*, Na_ values under each treatment were always significantly higher than the SA_*Ca*, Na_ values obtained under 171 mm NaCl treatment. Stresses at different concentrations of NaCl all caused the SA _*Ca*, Na_ of *S. matsudana* Koidz roots to show an overall downward trend as the treatment time increased. In the first 5 days, the ST _k, Na_ of leaves showed a decreasing trend after increasing as the degree of stress intensified, and the decrease was not significant. When the concentration of NaCl was greater than 342 mm, the ST _k, Na_ of leaves gradually decreased as the treatment time increased. On the 1st day, as NaCl stress intensified, the ST _*Ca*, Na_ values of leaves showed an increasing trend, and the difference was significant. In general, the ST _*Ca*, Na_ values of leaves under different salt concentrations gradually decreased as the treatment time increased, and the difference was significant.

### Correlation Analysis of Leaf Na^+^/K^+^ and Na^+^/Ca^2+^ Under Salt Stress

**FIGURE 2:**
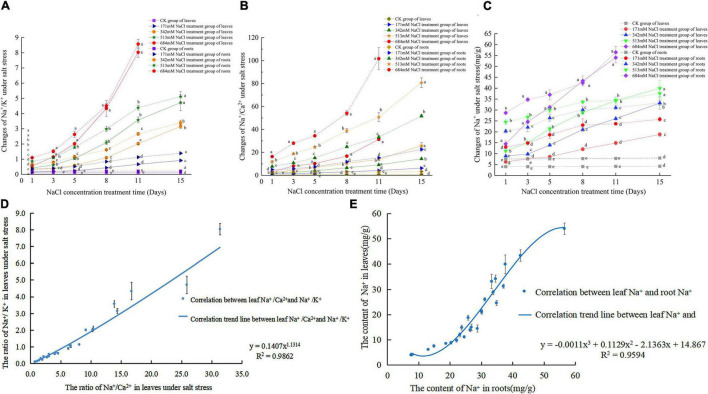
Changes and correlation of Na^+^,Na^+^/ K^+^ and Na^+^/ Ca^2+^ in roots and leaves of *S. matsudana* under salt stress; **(A)** changes of Na^+^/ K^+^ in roots and leaves under salt stress; **(B)** changes of Na^+^/ Ca^2+^ in roots and leaves under salt stress; **(C)** changes of Na^+^ in roots and leaves under salt stress; **(D)** correlation between Na^+^/ K^+^ and Na^+^/ Ca^2+^ in leaves under salt stress; **(E)** correlation between Na^+^ in leaves and roots under salt stress.

With the increase in NaCl treatment days, the Na^+^/K^+^ of roots and leaves showed an increasing trend, and the increasing rate increased faster than that noted before the 5th day after treatment (Figure 2A). In particular, 684 mm NaCl increased with increasing treatment days. In addition, when the NaCl treatment time was the same, the Na^+^/K^+^ of roots and leaves increased with increasing NaCl stress, and the Na^+^/K^+^ of roots was greater than that of leaves. With the extension of NaCl treatment time, the Na^+^/Ca^2+^ in roots and leaves also showed a gradual upward trend (Figure 2B). Under the same NaCl treatment time, Na^+^/Ca^2+^ also increased with increasing NaCl stress concentration. Under the same concentration of NaCl, the Na^+^/Ca^2+^ levels of the root system were greater than that of the leaves. When NaCl is greater than 342 mm, as the concentration of NaCl increases, Na^+^/Ca^2+^ increases sharply. When NaCl is less than 342 mm, Na^+^/Ca^2+^ rises slowly in roots and leaves. In the first 8 days, the Na^+^ content in roots was higher than that in leaves at the same time (Figure 2C). Figure 2D shows that after NaCl stress treatment, Na^+^/K^+^ and Na^+^/Ca^2+^ in leaves showed a linear correlation Eq. 9 (*R*^2^ = 0.9862). Figure 2E shows that the correlation equation of Na^+^ content in roots and leaves under salt stress is Eq. 10 (*R*^2^ = 0.9594).


(9) $$\text{y}=0.1407\,{\text{x}}^{1.1314}$$

(10) $$\text{y}=-0.0011\,{\text{x}}^{3}+0.1129\,{\text{x}}^{2}-2.1363\,{\text{x}}^{1}+14.867$$

### Analysis of the Correlation of Chlorophyll and Leaf Ions of *Salix matsudana* Koidz Under Salt Stress

**FIGURE 3:**
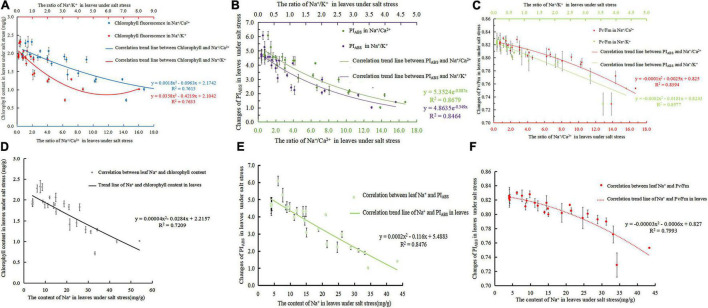
The ion ratio of salt stress to leaves was correlated with chlorophyll and fluorescence parameters; **(A)** correlation of Na^+^/ Ca^2+^ and Na^+^/ K^+^ to chlorophyll content in the leaves of *S. matsudana* under salt stress; **(B)** correlation between Na^+^/Ca^2+^, Na^+^/ K^+^, and PI_*ABS*_ in the leaves of *S. matsudana* under salt stress; **(C)** correlation of Na^+^/Ca^2+^ and Na^+^/K^+^ in the leaves of *Salix matsudana* with Fv/Fm under salt stress; **(D)** correlation between Na^+^ and chlorophyll content in *S. matsulosa* leaves under salt stress; **(E)** correlation between Na^+^ and PI_*ABS*_ in *S. matsulosa* leaves under salt stress; **(F)** correlation between Na^+^ and Fv/Fm in *S. matsulosa* leaves under salt stress.

With the increase in Na^+^/Ca^2+^ caused by NaCl stress, the chlorophyll content decreased significantly. A negative linear correlation was noted between Na^+^/Ca^2+^ and chlorophyll content under NaCl stress (Figure 3A). The fitted curve was Eq. 11 (*R*^2^ = 0.7615). Na^+^/K^+^ increased and chlorophyll decreased under salt stress. The correlation between Na^+^/K^+^ and chlorophyll content under salt stress was a negative exponential, and the correlation equation was Eq. 12 (*R*^2^ = 0.7633). In addition, Na^+^/K^+^ were negatively correlated with chlorophyll content. With the increase in salt stress, Na^+^ continued to accumulate, and Ca^2+^ and K^+^ were gradually displaced, resulting in a significant increase in Na^+^, Na^+^/Ca^2+^, and Na^+^/K^+^ and a decrease in chlorophyll content. Figure 3D shows the correlation equation between Na^+^ content and chlorophyll content in leaves as Eq. 13 (*R*^2^ = 0.7209).


(11) $$\text{y}=-0.0018\,{\text{x}}^{2}-0.0963\,{\text{x}}^{1}+2.1742$$

(12) $$\text{y}=0.0358\,{\text{x}}^{2}-0.4219\,{\text{x}}^{1}+2.10742$$

(13) $$\text{y}=0.00004\,{\text{x}}^{2}-0.0284\,{\text{x}}^{1}+2.2157$$

### Analysis of the Correlation Between Leaf Fluorescence Parameters and Ions Under Salt Stress

#### Analysis of the Correlation Between the Leaf Performance Index PI_*ABS*_ and Ions Under Salt Stress

As shown in Figure 3B, Na^+^/Ca^2+^ and PI_*ABS*_ are exponentially related (Eq. 14) (*R*^2^ = 0.8679). Na^+^/K^+^ and PI_*ABS*_ are also exponentially related (Eq. 15) (*R*^2^ = 0.8464). Figure 3E shows that the fitting curve of leaf Na^+^ and PI_*ABS*_ is Eq. 16 (*R*^2^ = 0.8476). As salt stress intensifies or time increases, Na^+^/Ca^2+^ and Na^+^/K^+^ increase, and PI_*ABS*_ decreases significantly. Both Na^+^/Ca^2+^ and Na^+^/K^+^ have a negative exponential relationship with PI_*ABS*_. As the ion concentration ratio increases, PI_*ABS*_ continues to decrease. The degree of correlation between Na^+^/Ca^2+^ and PI_*ABS*_ is greater than that between Na^+^/K^+^ and PI_*ABS*_.


(14) $$\text{y}=5.3524\,{\text{e}}^{-0.087\,x}$$

(15) $$\text{y}=4.8635\,{\text{e}}^{-0.349\,x}$$

(16) $$\text{y}=0.0002\,{\text{x}}^{2}-0.116\,{\text{x}}^{1}+5.4883$$

#### Analysis of the Correlation Between Leaf Maximum Photochemical Efficiency and Ions Under Salt Stress

Studies have confirmed that Fv/Fm can indicate the response of plants to stress environments (Zhang et al., 2009), so its decline is an important indicator of plants being inhibited by light. As shown in Figure 3C, the fitting relationship curves of Na^+^/Ca^2+^ and Na^+^/K^+^ with Fv/Fm under salt stress are Eq. 17 (*R*^2^ = 0.8394) and Eq. 18 (*R*^2^ = 0.8577). Figure 3F shows that the fitting equation of Na^+^ content and Fv/Fm in leaves was Eq. 19 (*R*^2^ = 0.7993). As the NaCl concentration intensifies or the time increases, both Na^+^/Ca^2+^ and Na^+^/K^+^ are linearly negatively correlated with the maximum photochemical efficiency Fv/Fm. With the increase in salt stress, Fv/Fm will be irreversibly damaged.


(17) $$\text{y}=-0.0001\,{\text{x}}^{2}-0.0025\,{\text{x}}^{1}+0.825$$

(18) $$\text{y}=-0.0002\,{\text{x}}^{2}-0.0181\,{\text{x}}^{1}+0.8243$$

(19) $$\text{y}=-0.00003\,{\text{x}}^{2}-0.0006\,{\text{x}}^{1}+0.827$$

### Analysis of the Correlation Between Photosynthetic Gas Parameters and Ions in Leaves Under Salt Stress

#### Analysis of the Correlation Between Leaf Net Photosynthetic Rate and Ions Under Salt Stress

**FIGURE 4:**
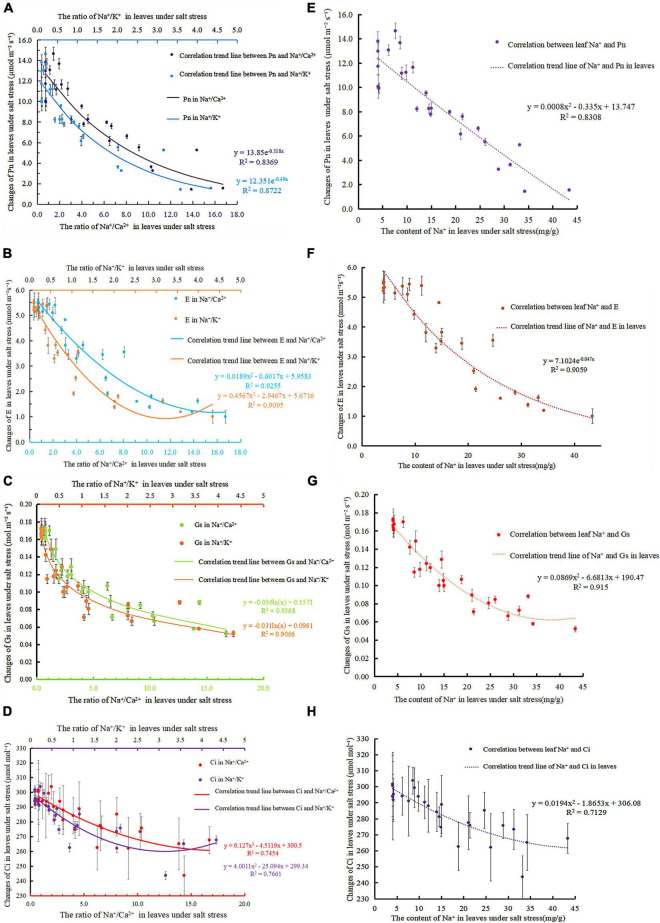
Correlation between photosynthetic gas parameters and ions in leaves under salt stress; **(A)** correlation of Na^+^/ Ca^2+^ and Na^+^/ K^+^ in the leaves of *S. matsudana* to Pn under salt stress; **(B)** correlation of Na^+^/ Ca^2+^ and Na^+^/ K^+^ in the leaves of *S. matsudana* to E under salt stress; **(C)** analysis of the correlation of Na^+^/Ca^2+^ and Na^+^/K^+^ in the leaves of *S. matsudana* with Gs under salt stress; **(D)** correlation of Na^+^/Ca^2+^ and Na^+^/ K^+^ in the leaves of *S. matsudana* to Ci under salt stress; **(E)** correlation of Na^+^ and Pn in the leaves of *S. matsudana* under salt stress; **(F)** correlation of Na^+^ in the leaves of *S. matsudana* to E under salt stress; **(G)** correlation of Na^+^ in the leaves of *S. matsudana* with Gs under salt stress; **(H)** correlation of Na^+^ in the leaves of *S. matsudana* with Ci under salt stress.

Photosynthetic rate is an important parameter to measure the strength of plant photosynthesis that is a direct reflection of the function of the photosynthetic system and a key indicator of whether the plant’s photosynthetic system is normal. Under salt stress, Na^+^/Ca^2+^, Na^+^/K^+^, and Pn showed exponential negative correlations (Figure 4A), and the correlations reached *R*^2^ = 0.8369 (Eq. 20) and *R*^2^ = 0.8722 (Eq. 21). Figure 4E shows that the fitting equation of Na^+^ content and Pn in leaves was Eq. 22 (*R*^2^ = 0.8308). As salt stress intensified, Na^+^/Ca^2+^, and Na^+^/K^+^ increased, whereas Pn decreased. Na^+^/Ca^2+^ and Na^+^/K^+^ in leaves can reflect the changes in the photosynthetic capacity of leaves and reveal the reason for the reduction in the photosynthetic rate to a certain extent.


(20) $$\text{y}=13.85\,{\text{e}}^{-0.118\,x}$$

(21) $$\text{y}=12.351\,{\text{e}}^{-0.49\,x}$$

(22) $$\text{y}=0.0008\,{\text{x}}^{2}-0.335\,{\text{x}}^{1}+13.747$$

#### Analysis of the Correlation Between Leaf Transpiration E and Ions Under Salt Stress

As shown in Figures 4B,E decreases significantly with increasing Na^+^/Ca^2+^ concentration. Na^+^/Ca^2+^ and E have a negative correlation, and the fitted relationship curve is Eq. 23 (*R*^2^ = 0.9255). Na^+^/K^+^ increased with increasing NaCl stress, whereas E showed a downward trend. The correlation between Na^+^/K^+^ and E is exponential, and the correlation equation is Eq. 24 (*R*^2^ = 0.9095). The correlation equation between Na^+^ content and E in leaves from Figure 4F was Eq. 25 (*R*^2^ = 0.9059).


(23) $$\text{y}=0.0189\,{\text{x}}^{2}-0.6017\,{\text{x}}^{1}+5.9583$$

(24) $$\text{y}=0.4567\,{\text{x}}^{2}-2.9467\,{\text{x}}^{1}+5.6716$$

(25) $$\text{y}=7.1024\,{\text{e}}^{-0.047\,x}$$

#### Analysis of the Correlation Between Leaf Stomatal Conductance and Ions Under Salt Stress

Figure 4C shows that Na^+^/Ca^2+^ and Gs have a negative logarithmic correlation under salt stress with the correlation reaching *R*^2^ = 0.9368 (Eq. 26). Under salt stress, Na^+^/K^+^ and Gs have a negative logarithmic correlation with the correlation reaching *R*^2^ = 0.9066 (Eq. 27). Figure 4G shows the correlation equation between Na^+^ content and Gs in leaves under salt stress : Eq. 28 (*R*^2^ = 0.915).


(26) $$\text{y}=-35.53\,\ln\,\left({\text{x}}^{1}\right)+157.14$$

(27) $$\text{y}=-30.62\,\ln\,\left({\text{x}}^{1}\right)+96.097$$

(28) $$\text{y}=0.0869\,{\text{x}}^{2}-6.6813\,{\text{x}}^{1}+190.47$$

#### Analysis of the Correlation Between Intercellular Carbon Dioxide Concentration and Ions in Leaves Under Salt Stress

As salt stress intensifies, the continuous loss of Ca^2+^ leads to an increase in the ratio of Na^+^/Ca^2+^. Photosynthesis is subsequently affected, and Ci decreases accordingly (Figure 4D). Under salt stress, Na^+^/Ca^2+^ and Ci show a negative correlation relationship, and the fitted relationship curve is Eq. 29 (*R*^2^ = 0.7454). As shown in Figure 4D, under NaCl stress, the ratio of Na^+^/K^+^ increased continuously, and Ci decreased accordingly. At the initial stage of salt stress, when the ratio of Na^+^/K^+^ is less than 1, Ci is at a higher level. Then, as the ratio of Na^+^/K^+^ increases, Ci gradually decreases. Under salt stress, Na^+^/K^+^ and Ci show a negative logarithmic relationship, and the fitted relationship curve is Eq. 30 (*R*^2^ = 0.7661). Figure 4H shows that the correlation equation between Na^+^ content and Ci in leaves under salt stress was Eq. 31 (*R*^2^ = 0.7129).


(29) $$\text{y}=0.127\,{\text{x}}^{2}-4.5119\,{\text{x}}^{1}+300.5$$

(30) $$\text{y}=4.0011\,{\text{x}}^{2}-25.094\,{\text{x}}^{1}+299.34$$

(31) $$\text{y}=0.0194\,{\text{x}}^{2}-1.8653\,{\text{x}}^{1}+306.08$$

## Discussion

### Relationship Between Na^+^ in Roots and Leaves and Na^+^/K^+^ and Na^+^/Ca^2+^ in Leaves Under Salt Stress

The damage of salt stress to plants starts from the root system, and the cell membrane is the initial location of salt stress (Meng et al., 1996). The cell plasma membrane is first damaged by saline ions, leading to the continuous exosmosis of mineral ions, such as P^+^, Mg^2+^, K^+^, and Ca^2+^, in the cell; the entry of external saline ions, such as Na^+^ and Cl^–^, into the cell; disturbed physiological function of the cell membrane, affected transport of Na^+^, K^+^, and Ca^2+^ to the ground; changes in ion content in the leaves and irreversible damage to the corresponding functions of leaves (Zhou et al., 2021). Therefore, their water content suggests that the ability of roots and leaves to hold water is threatened. The ratio of root to leaf dry weight was also changed because growth was inhibited by salt stress. The change in the ion distribution law mirrors the damage extent of adverse environments to cells, and plants maintain the balance of nutrition by enhancing the absorption and transport of ions, which also reflects to a certain extent the ability of plants to resist stress. Therefore, the changes in Na^+^/K^+^ and Na^+^/Ca^2+^ can represent the degree of plant damage and the change in nutrient balance in plants.

K^+^ and Na^+^ may compete for absorption sites on the cell membrane (Tyerman and Skerrett, 1998; Frans and Anna, 1999; Mark and Romola, 2003). Therefore, salt stress not only induces K^+^ efflux from cells but also reduces K^+^ absorption by cells. When the K^+^ efflux increases but the influx decreases, the K^+^ content in the cell will be greatly reduced, the Na^+^/K^+^ ratio in the cell will be changed, and the plant will be injured. Calcium is an essential mineral element for plants and participates in various reactions, including stress and biological stimuli, such as salt and drought (Kudla et al., 2010). In this experiment, the selective absorption capacity of roots for K^+^ and Ca^2+^ at the initial stage of stress increased as the stress intensified; the selective absorption capacity of roots for different salt stress levels increased as the treatment time increased. This finding indicates that large amounts of Na^+^ in the roots of the plant in the early stage result in a violent stress response of root cells. Then, root cells increase the selective absorption capacity of K^+^ and Ca^2+^ and control the transport of ions toward the ground to sustain low cell osmotic potential and cell membrane stability. This phenomenon is also the reason why the Na^+^ content in roots is greater than that in leaves.

Under the same salt concentration and the same treatment time, the selective absorption of K^+^ by roots was stronger than that of Ca^2+^, and the selective transport of Ca^2+^ by leaves was stronger than that of K^+^. Under different treatment times, Na^+^/K^+^ and Na^+^/Ca^2+^ levels in roots were greater than those in leaves, indicating that roots have a certain role in retaining Na^+^ ions under salt stress and increasing the selective absorption and transportation of K^+^ and Ca^2+^. However, after reaching a certain degree of salt stress, the limited ability cannot offset the harm caused by ions. Once this ability is disrupted, the Na^+^/K^+^ and Na^+^/Ca^2+^ of plant roots and leaves will increase significantly. Studies have shown that the increase in Ca^2+^ content in cells under salt stress can restrain the efflux of K^+^ (Guo et al., 2015), thereby relieving the damage caused by salt stress to plants. The correlation of Na^+^/K^+^ and Na^+^/Ca^2+^ in leaves reached 0.9614, indicating that K^+^ and Ca^2+^ are closely related under salt stress. *S. matsudana* Koidz will adjust the root system’s selective absorption of K^+^ and Ca^2+^ from the soil and transport it to the leaves to minimize the adverse effects caused by the blocked absorption function. However, this ability has a certain limit, as too high a concentration of salt stress will directly destroy the ability of selective absorption and cause irreparable damage to plants.

### Relationship Between Na^+^/K^+^ and Na^+^/Ca^2+^ With Chlorophyll and Fluorescence Under Salt Stress

The more chlorophyll on the thylakoid membrane on plant leaves, the stronger the photosynthesis power of plants. This is the basic ability of plants to maintain life activities and the basis for judging the ability of plants to resist stress (Zhang et al., 2013; Wang et al., 2021). In other respects, photosynthesis also affects the accumulation of plant dry matter and final yield (Thakur and Kumar, 2021). Yang’s experiment showed that damage to chloroplast membrane structure could be observed under salt stress (Yang et al., 2020). Studies have shown that the chlorophyll content in the leaves of Jerusalem artichoke (Sun W. J. et al., 2021) and oats (Li et al., 2021) decreases significantly under salt stress. Therefore, many researchers regard the chlorophyll content under salt stress as an important index to measure the salt tolerance of plants. In this study, the correlations between Na^+^/K^+^ and chlorophyll and between Na^+^/Ca^2+^ and chlorophyll were 0.7633 and 0.7615, respectively. This finding indicates that the correlation between the ion concentration ratio and chlorophyll is very high under salt stress, but no remarkable difference was noted between them. The correlation between Na^+^/K^+^ and chlorophyll was greater than that between Na^+^/Ca^2+^ and chlorophyll. This may be because Na^+^ is flushed into the cells of salt-stressed plants, causing the accumulation of Na^+^ and affecting the absorption of K^+^ by plants. This process induces ion toxicity and results in a higher correlation between Na^+^/K^+^ and chlorophyll (Zhang et al., 2022).

Fluorescence parameters are often used to examine the relationship between the photosynthesis of plants and the environment where they live (Zhao et al., 2021). Researchers generally observe the photosynthetic physiological status of plants under salt stress by means of the OJIP curve, Fv/Fm and PI_*ABS*_. The Fv/Fm and PI_*ABS*_ of PS II are important indices to evaluate whether plants are stressed. The more intense the stress, the lower the values of both and the greater the damage to plant photosynthetic capacity. Studies have shown that the ratio of Fv/Fm is stable under non-stress conditions, and the ratio decreases obviously when stress conditions exist in the environment (Zhao et al., 2021), which was demonstrated by salt stress experiments with mangosteen seedlings (Li et al., 2005; Shu et al., 2021). In this study, the correlation between Na^+^/Ca^2+^ and PI_*ABS*_ (*R*^2^ = 0.8679) was greater than that between Na^+^/K^+^ and PI_*ABS*_ (*R*^2^ = 0.8464). The correlation between Na^+^/K^+^ and Fv/Fm (*R*^2^ = 0.8577) was greater than that between Na^+^/Ca^2+^ and Fv/Fm (*R*^2^ = 0.8394). The correlation between Na^+^/K^+^ and Fv/Fm (*R*^2^ = 0.8577) was the highest among these indices, indicating that the chlorophyll fluorescence kinetic parameters can also reflect the photoenergy utilization status of photosystem II (Yu and Guy, 2004; Zhao et al., 2006). Under salt stress, *S. matsudana* Koidz leaves exhibited photoinhibition, which resulted in a notable decrease in Fv/Fm efficiency. However, the change in chlorophyll was relatively slow, which might be due to the gradual increase in chlorophyll content under low salt stress to increase light energy utilization. Under high salt stress, the synthesis and decomposition of chlorophyll were affected. The decomposition process of chlorophyll lagged somewhat, so the correlation with ions was lower than other indicators.

### Relationship of Na^+^/K^+^ and Na^+^/Ca^2+^ in Leaves to Gas Exchange Parameters Under Salt Stress

Salt stress can affect the normal growth of plants by reducing the photosynthesis of plants, so photosynthetic parameters can be used as crucial indicators to judge the salt–alkali tolerance of plants (Zhou et al., 2021). Photosynthetic parameters mainly include the net photosynthetic rate (Pn), transpiration rate (E), stomatal conductance (Gs), and intercellular CO_2_ concentration (Ci). Gs and E exhibit a high correlation with Na^+^/Ca^2+^ and Na^+^/K^+^, respectively, and Gs has the highest correlation with Na^+^/Ca^2+^ (*R*^2^ = 0.9368). Stomatal limitation is tightly related to the photosynthetic rate of plants under adverse stress (Yang et al., 2019). Normally, under mild stress, stomatal limitation is the dominant reason for the reduction in the plant photosynthetic rate (Sarabi et al., 2019). Studies have indicated that salt stress causes changes in plant osmotic potential, thereby affecting the plant transpiration rate (Cambridge et al., 2017; Wang L. H. et al., 2017). With increasing salt concentration, the Gs of *S. matsudana* Koidz leaves is affected by osmotic stress and thus decreases. Then, the stomatal resistance of the leaves increases. By inhibiting E to increase the water potential, the influence of salt stress is reduced, and finally, the loss of water is reduced to ensure the stability of photosynthesis. Therefore, this notion may explain the higher correlation between Gs and E and the ion concentration ratio.

As the main factor affecting photosynthesis, transpiration, and respiration, stomatal conductance affects the circulation of oxygen, carbon dioxide, and water in plants (Xu P. Y. et al., 2020; Yan et al., 2020). Studies have shown that the Gs of plant leaves is significantly reduced due to intensified salt stress (Mafakheri et al., 2010; Zhao et al., 2019). The change direction of Ci is not only one of the main reasons for determining the photosynthetic rate change but also an indispensable basis for judging whether it is a stomatal factor. The correlation between it and Na^+^/Ca^2+^, Na^+^/K^+^ is relatively low (*R*^2^ = 0.7183, *R*^2^ = 0.7229). This finding may be attributed to the notion that the factors affecting Ci include not only Gs but also the photosynthetic activity of mesophyll cells, mesophyll conductance and the CO_2_ concentration in the air around leaves, which cause the response of Ci to lag behind other photosynthetic gas parameters.

In this experiment, the correlation between Na^+^/Ca^2+^, Na^+^/K^+^, and Pn was lower than their correlation with E, which is different from the results of previous studies. Although many studies believe that the Pn of leaves under salt stress is a more accurate indicator that can directly reflect plant salt tolerance, its correlation with the transport concentration ratio of leaf ions is lower than that between other photosynthetic gas parameters and the leaf ion transport concentration ratio. This finding indicates that although Pn and the leaf ion concentration ratio are highly related, there are still differences in the sensitivity of net photosynthetic parameters as an indicator for different types of plants.

## Conclusion

By increasing the ratio of dry weight and water content of roots and leaves, *S. matsudana* could lessen the damage caused by salt stress to a certain extent, which was of significance to the survival of plants under adverse situations. Different salt conditions have different effects on the ratio of ion absorption and transport, photosynthetic parameters, and fluorescence parameters of *S. matsudana* Koidz. The ratio of ion content in leaves had different correlations with photosynthetic and fluorescence parameters. Some scholars think that it is impossible to judge the salt tolerance level of plants by using a certain indicator or a certain type of indicator and ignore the correlation of each indicator in the process of development. Therefore, it is very important to select and evaluate the key indicator that has a high correlation coefficient with salt tolerance for the appraisal of salt tolerance of plants.

In this experiment, the loss of Ca^2+^ in roots was more serious than the loss of K^+^ under salt stress, and the selective transport of Ca^2+^ in leaves was stronger than that of K^+^, resulting in higher Na^+^/Ca^2+^ than Na^+^/K^+^ in roots and leaves, indicating that Na^+^/Ca^2+^ is more responsive to salt stress than Na^+^/K^+^. Under salt stress, the leaf Na^+^, Na^+^/Ca^2+^, and Na^+^/K^+^ contents, which are commonly used to evaluate the salt tolerance of plants, were compared with the above parameters. The correlation between leaf Na^+^ content and these indices was lower than that of Na^+^/Ca^2+^ and Na^+^/K^+^. The correlations of the measured parameters with Na^+^/Ca^2+^ and Na^+^/K^+^ were examined when evaluating the salt tolerance of *S. matsudana* Koidz. Among the photosynthetic gas parameters, Gs and E were highly correlated with Na^+^/Ca^2+^ and Na^+^/K^+^, and the correlation with Na^+^/Ca^2+^ was the highest, indicating that the influence of Na^+^/Ca^2+^ on photosynthetic parameters is more obvious than that of Na^+^/K^+^. Among all the parameters, the correlation between Ci of photosynthetic gas parameters and the two was the lowest followed by the correlation between Pn and Na^+^/Ca^2+^. The correlation of the chlorophyll fluorescence parameters PI_*ABS*_ and Fv/Fm to Na^+^/K^+^ was generally higher than that with Na^+^/Ca^2+^. The correlations of Na^+^/K^+^ and Na^+^/Ca^2+^ with chlorophyll content were basically the same.

Overall, with increasing time and salt concentration, Na^+^/Ca^2+^ in leaves showed dynamic accumulation changes, which ultimately affected the performance of the photosynthetic system, and the Gs and E of leaves were greatly affected. In addition, the correlation between Na^+^/Ca^2+^ and photosynthesis and fluorescence indicators is generally high, and it has a greater impact on the photosynthetic system. The research results provide a basis for understanding the relationship between ion absorption and distribution, fluorescence and photosynthetic parameters of *S. matsudana* in a saline environment.

We chose indicators of sodium, sodium potassium, sodium calcium, which are often used by scholars to evaluate salt tolerance. Because magnesium is an indispensable element in the molecular structure of chlorophyll, the synthesis of chlorophyll is hindered when magnesium is deficient, which also affects photosynthesis. This has been confirmed by Li et al. (2020): In chloroplasts, Mg^2+^ is dependent on transport genes, resulting in a phenomenon of diurnal fluctuation, which regulates plant photosynthesis by affecting enzyme activity. Therefore, increasing the input of Mg^2+^ ions may be a potential method to improve the photosynthetic efficiency of plants. Iron is not only a component of cytochrome and non-heme ferritin in photosynthesis but also an important raw material for the synthesis of chlorophyll–protein complex in chloroplasts. Focusing on the effects of iron ions, magnesium ions, the sodium-iron ratio, and the sodium-magnesium ratio on photosynthesis in future research will be more helpful to clarify the overall impact of salt stress ion allocation on the photosynthetic system.

## Data Availability Statement

The original contributions presented in the study are included in the article/Supplementary Material, further inquiries can be directed to the corresponding author/s.

## Author Contributions

XR and XW conceptualized the manuscript. XR, XW, CM, HL, BL, and XH made the data curation. XW, XR, and BL made formal analysis. HL and BL made funding acquisition. CM and BL made the methodology. BL and XW provided the resources and wrote the original draft. CM, HL, and BL wrote the review. All authors contributed to the article and approved the submitted version.

## Conflict of Interest

The authors declare that the research was conducted in the absence of any commercial or financial relationships that could be construed as a potential conflict of interest.

## Publisher’s Note

All claims expressed in this article are solely those of the authors and do not necessarily represent those of their affiliated organizations, or those of the publisher, the editors and the reviewers. Any product that may be evaluated in this article, or claim that may be made by its manufacturer, is not guaranteed or endorsed by the publisher.
