# Supplementary material for: Study on the Relationship of Ions (Na, K, Ca) Absorption and Distribution to Photosynthetic Response of Salix matsudana Koidz Under Salt Stress
Source: Front Plant Sci. 2022 May 3;13:860111. doi: 10.3389/fpls.2022.860111 (PMC9111522; doi:10.3389/fpls.2022.860111)
Supplement: Supplementary file 1 [file Data_Sheet_1.DOCX]

**Supporting Information**

Study on the relationship of ion absorption and distribution to photosynthetic response of *Salix matsudana* Koidz in saline environment

**Summary**

**Table S1** Effects of salt stress on ratio of roots and leaves dry weight of *Salix matsudana.* (data in Fig.1A)

**Table S2** Effects of salt stress on water content of roots and leaves of *Salix matsulosa.* (data in Fig.1B)

**Table S3** Effect of salt stress on selective uptake and transportation of Ca^2+^ in roots and leaves of *Salix matsulosa* (data in Fig.1C )

**Table S4** Effect of salt stress on selective uptake and transportation of K^+^ in roots and leaves of *Salix matsulosa* (data in Fig.1D )

**Table S5** Changes of Na^+^/ K^+^ in roots and leaves under salt stress **(**data in Fig.2A)

**Table S6** Changes of Na^+^/ Ca^2+^ in roots and leaves under salt stress **(**data in Fig.2B)

**Table S7** Changes of Na^+^ in roots and leaves under salt stress **(**data in Fig.2C)

**Table S8** Correlation between Na^+^/ K^+^ and Na^+^/ Ca^2+^ in leaves under salt stress **(**data in Fig.2D)

**Table S9** Correlation between Na^+^ in leaves and roots under salt stress **(**data in Fig.2E)

**Table S10** Correlation of Na^+^/ Ca^2+^ and Na^+^/ K^+^ to Chlorophyll Content in the Leaves of *Salix matsudana* under Salt Stress **(**data in Fig.3A)

**Table S11** Correlation between Na^+^/Ca^2+^, Na^+^/ K^+^ and PI_ABS_ in the Leaves of *Salix matsudana* under Salt Stress **(**data in Fig.3B)

**Table S12** Correlation of Na^+^/Ca^2+^ and Na^+^/K^+^ in the Leaves of *Salix matsudana* with Fv/Fm under Salt Stress. **(**data in Fig.3C)

**Table S13** Correlation between Na^+^ and chlorophyll content in *salix matsulosa* leaves under salt stress **(**data in Fig.3D)

**Table S14** Correlation between Na^+^ and PI_ABS_ in s*alix matsulosa* leaves under salt stress **(**data in Fig.3E)

**Table S15** Correlation between Na^+^ and Fv/Fm in *salix matsulosa* leaves under salt stress **(**data in Fig.3F)

**Table S16** Correlation of Na^+^/ Ca^2+^ and Na^+^/ K^+^ in the Leaves of *Salix matsudana* to Pn under Salt Stress **(**data in Fig.4A)

**Table S17** Correlation of Na^+^/ Ca^2+^ and Na^+^/ K^+^ in the Leaves of *Salix matsudana* to E under Salt Stress **(**data in Fig.4B)

**Table S18** Correlation of Na^+^/Ca^2+^ and Na^+^/K^+^ in the Leaves of *Salix matsudana* with Gs under Salt Stress **(**data in Fig.4C)

**Table S19** Correlation of Na^+^/Ca^2+^ and Na^+^/K^+^ in the Leaves of *Salix matsudana* with Ci under Salt Stress **(**data in Fig.4D)

**Table S20** Correlation of Na^+^ and Pn in the Leaves of *Salix matsudana* under Salt Stress **(**data in Fig.4E)

**Table S21** Correlation of Na^+^ in the Leaves of *Salix matsudana* to E under Salt Stress. **(**data in Fig.4F)

**Table S22** Correlation of Na^+^ in the Leaves of *Salix matsudana* with Gs under Salt Stress(data in Fig.4G)

**Table S23** Correlation of Na^+^ in the Leaves of *Salix matsudana* with Ci under Salt Stress **(**data in Fig.4H)

**Fig. S1** Effects of salt stress on ratio of roots and leaves dry weight of *Salix matsudana.* (data in Fig.1A)

**Fig. S2** Effects of salt stress on water content of roots and leaves of *Salix matsulosa.* (data in Fig.1B)

**Fig. S3** Effect of salt stress on selective uptake and transportation of Ca^2+^ in roots and leaves of *Salix matsudana* (data in Fig.1C )

**Fig. S4** Effect of salt stress on selective uptake and transportation of K^+^ in roots and leaves of *Salix matsudana* (data in Fig.1D )

**Fig. S5** Changes of Na^+^/ K^+^ in roots and leaves under salt stress(data in Fig.2A )

**Fig. S6** Changes of Na^+^/ Ca^2+^ in roots and leaves under salt stress(data in Fig.2B )

**Fig. S7** Changes of Na^+^ in roots and leaves under salt stress (data in Fig.2C )

**Fig. S8** Correlation between Na^+^/ K^+^ and Na^+^/ Ca^2+^ in leaves under salt stress (data in Fig.2D )

**Fig. S9** Correlation between Na^+^ in leaves and roots under salt stress (data in Fig.2E )

**Fig. S10** Correlation of Na^+^/ Ca^2+^ and Na^+^/ K^+^ to Chlorophyll Content in the Leaves of *Salix matsudana* under Salt Stress (data in Fig.3A )

**Fig. S11** Correlation between Na^+^/Ca^2+^, Na^+^/ K^+^ and PI_ABS_ in the Leaves of *Salix matsudana* under Salt Stress (data in Fig.3B )

**Fig. S12** Correlation of Na^+^/Ca^2+^ and Na^+^/K^+^ in the Leaves of *Salix matsudana* with Fv/Fm under Salt Stress (data in Fig.3C )

**Fig. S13** Correlation between Na^+^ and chlorophyll content in *salix matsulosa* leaves under salt stress (data in Fig.3D )

**Fig. S14** Correlation between Na^+^ and PI_ABS_ in *salix matsulosa* leaves under salt stress (data in Fig.3E)

**Fig. S15** Correlation between Na^+^ and Fv/Fm in *salix matsulosa* leaves under salt stress (data in Fig.3F)

**Fig. S16** Correlation of Na^+^/ Ca^2+^ and Na^+^/ K^+^ in the Leaves of *Salix matsudana* to Pn under Salt Stress (data in Fig.4A)

**Fig. S17** Correlation of Na^+^/ Ca^2+^ and Na^+^/ K^+^ in the Leaves of *Salix matsudana* to E under Salt Stress (data in Fig.4B)

**Fig. S18** Analysis of the Correlation of Na^+^/Ca^2+^ and Na^+^/K^+^ in the Leaves of *Salix matsudana* with Gs under Salt Stress (data in Fig.4C)

**Fig. S19** Correlation of Na^+^/Ca^2+^ and Na^+^/ K^+^ in the Leaves of *Salix matsudana* to Ci under Salt Stress (data in Fig.4D)

**Fig. S20** Correlation of Na^+^ and Pn in the Leaves of *Salix matsudana* under Salt Stress (data in Fig.4E)

**Fig. S21** Correlation of Na^+^ in the Leaves of *Salix matsudana* to E under Salt Stress (data in Fig.4F)

**Fig. S22** Correlation of Na^+^ in the Leaves of *Salix matsudana* with Gs under Salt Stress (data in Fig.4G)

**Fig. S23** Correlation of Na^+^ in the Leaves of *Salix matsudana* with Gs under Salt Stress (data in Fig.4H)

**Table S1**  Effects of salt stress on ratio of roots and leaves dry weight of *Salix matsudana.* (data in Fig.1A)

|  | NaCl Concentration(mm) | The processing time of NaCl concentration(Days) | | | | | |
| --- | --- | --- | --- | --- | --- | --- | --- |
|  |  | 1 | 3 | 5 | 8 | 11 | 15 |
| Dry weight of roots/dry weight of leaves | CK | 0.550±0.010b | 0.559±0.018b | 0.559±0.011c | 0.561±0.026c | 0.587±0.016c | 0.611±0.031c |
|  | 171 | 0.561±0.019ab | 0.555±0.010b | 0.574±0.020bc | 0.585±0.025c | 0.621±0.013bc | 0.646±0.004bc |
|  | 342 | 0.550±0.023b | 0.575±0.014ab | 0.596±0.021ab | 0.629±0.015b | 0.640±0.011b | 0.662±0.021b |
|  | 513 | 0.572±0.024ab | 0.589±0.011a | 0.615±0.018a | 0.649±0.010ab | 0.662±0.017b | 0.848±0.040a |
|  | 684 | 0.589±0.018a | 0.594±0.004a | 0.609±0.007a | 0.666±0.016a | 1.005±0.051a |  |
| Note: The number after ± is the standard deviation.Different small letters in the same column meant significant difference at 0.05 level among treatments. | | | | | | | |

**Table S2** Effects of salt stress on water content of roots and leaves of *Salix matsulosa.* (data in Fig.1B)

| Indicators | NaCl Concentration(mm) | The processing time of NaCl concentration(Days) | | | | | |
| --- | --- | --- | --- | --- | --- | --- | --- |
|  |  | 1 | 3 | 5 | 8 | 11 | 15 |
| Water content of leaves(%) | CK | 79.4±0.9ab | 80.2±0.8b | 81.2±0.8a | 82.2±0.6ab | 83.1±0.8a | 82.3±0.8a |
|  | 171 | 80.1±1.3a | 82.8±3.3a | 83.5±3.1a | 83.2±0.8a | 85.3±1.4a | 83.1±3.3a |
|  | 342 | 80.3±1.7a | 84.3±1.8a | 83.3±0.7a | 80.2±0.5b | 77.0±3.2b | 71.4±1.5b |
|  | 513 | 79.2±2.1ab | 84.3±1.2a | 82.0±2.3a | 75.2±2.3c | 68.2±1.7c | 52.1±1.7c |
|  | 684 | 78.2±2.6b | 84.7±1.5a | 75.8±1.4b | 70.5±1.5d | 53.3±1.9d |  |
| Water content of roots(%) | CK | 82.0±0.2a | 82.1±1.0b | 84.2±1.0b | 89.3±0.2a | 90.6±1.1a | 90.6±1.1a |
|  | 171 | 82.9±1.5a | 83.2±1.4ab | 85.5±0.4ab | 88.8±1.2a | 88.7±0.5b | 88.2±1.9b |
|  | 342 | 83.0±0.9a | 83.8±0.4ab | 86.7±0.6a | 88.3±0.5ab | 86.9±1.7c | 81.7±0.8c |
|  | 513 | 82.1±1.5a | 84.5±1.8ab | 86.2±1.5a | 87.8±1.3ab | 82.2±1.3d | 79.2±0.8d |
|  | 684 | 83.2±1.3a | 85.4±1.2a | 86.0±1.0ab | 86.8±1.0b | 83.1±0.8d |  |
| Note: The number after ± is the standard deviation.Different small letters in the same column meant significant difference at 0.05 level among treatments. | | | | | | | |

**Table S3** Effect of salt stress on selective uptake and transportation of Ca^2+^ in roots and leaves of *Salix matsulosa* (data in Fig.1C )

| Indicators | NaCl Concentration(mm) | The processing time of NaCl concentration(Days) | | | | | |
| --- | --- | --- | --- | --- | --- | --- | --- |
|  |  | 1 | 3 | 5 | 8 | 11 | 15 |
| ST_Ca, Na_ of leaves | CK | 3.856±0.159c | 4.028±0.211a | 3.866±0.049a | 3.932±0.044a | 3.902±0.314a | 4.206±0.275a |
|  | 171 | 4.434±0.133b | 3.956±0.233a | 3.830±0.326a | 3.885±0.426a | 3.667±0.082ab | 3.621±0.183b |
|  | 342 | 4.586±0.522b | 3.936±0.031a | 3.803±0.235a | 3.826±0.261a | 3.652±0.266ab | 3.606±0.205b |
|  | 513 | 5.385±0.370a | 4.124±0.404a | 3.685±0.109a | 3.753±0.120a | 3.663±0.195ab | 3.134±0.194c |
|  | 684 | 5.378±0.181a | 3.469±0.058b | 3.363±0.025b | 3.242±0.148b | 3.242±0.262b |  |
| SA_Ca, Na_ of roots | CK | ---- | ---- | ---- | ---- | ---- | ---- |
|  | 171 | 0.342±0.013b | 0.299±0.012a | 0.229±0.011a | 0.145±0.005a | 0.112±0.001a | 0.076±0.005a |
|  | 342 | 0.442±0.011a | 0.316±0.010a | 0.225±0.001a | 0.137±0.005a | 0.102±0.004b | 0.066±0.001b |
|  | 513 | 0.441±0.037a | 0.274±0.011b | 0.209±0.007b | 0.132±0.002b | 0.101±0.004b | 0.064±0.003b |
|  | 684 | 0.416±0.009a | 0.244±0.010c | 0.198±0.005b | 0.126±0.003c | 0.067±0.006c |  |
| **Note:** The number after ± is the standard deviation.Different small letters in the same column meant significant difference at 0.05 level among treatments. | | | | | | | |

**Table S4** Effect of salt stress on selective uptake and transportation of K^+^ in roots and leaves of *Salix matsulosa* (data in Fig.1D )

| **Fig 1 d.** Effect of salt stress on selective uptake and transportation of K^+^ in roots and leaves of *Salix matsulosa* | | | | | | | |
| --- | --- | --- | --- | --- | --- | --- | --- |
| Indicators | NaCl Concentration(mm) | The processing time of NaCl concentration(Days) | | | | | |
|  |  | 1 | 3 | 5 | 8 | 11 | 15 |
| STK, Na of leaves | CK | 2.066±0.133b | 1.949±0.079b | 2.050±0.065a | 1.990±0.056a | 1.948±0.089a | 1.942±0.053a |
|  | 171 | 2.382±0.091a | 1.990±0.046b | 2.238±0.150a | 1.928±0.107a | 1.705±0.139b | 1.522±0.096b |
|  | 342 | 2.060±0.083b | 2.125±0.047a | 2.003±0.230a | 1.480±0.135b | 1.322±0.071c | 1.077±0.042c |
|  | 513 | 2.015±0.135b | 1.768±0.163c | 1.714±0.159b | 1.423±0.020b | 1.230±0.098cd | 1.094±0.125c |
|  | 684 | 1.972±0.129b | 1.327±0.057d | 1.308±0.066c | 1.048±0.092c | 1.069±0.069d |  |
| SAK, Na of roots | CK | ---- | ---- | ---- | ---- | ---- | ---- |
|  | 171 | 3.956±0.036c | 3.556±0.160b | 2.799±0.231a | 1.718±0.012b | 1.278±0.009a | 1.041±0.023a |
|  | 342 | 4.463±0.140b | 3.623±0.045b | 2.481±0.112ab | 1.792±0.064a | 1.085±0.035b | 0.852±0.037b |
|  | 513 | 5.068±0.227a | 3.984±0.168a | 2.431±0.048b | 1.450±0.049c | 0.987±0.037c | 0.846±0.006b |
|  | 684 | 5.273±0.214a | 3.805±0.032ab | 2.204±0.183b | 1.281±0.041d | 0.675±0.016d |  |
| **Note:** The number after ± is the standard deviation.Different small letters in the same column meant significant difference at 0.05 level among treatments. | | | | | | | |

**Table S5** Changes of Na^+^/ K^+^ in roots and leaves under salt stress **(**data in Fig.2A)

| Indicators | NaCl Concentration(mm) | The processing time of NaCl concentration(Days) | | | | | |
| --- | --- | --- | --- | --- | --- | --- | --- |
|  |  | 1 | 3 | 5 | 8 | 11 | 15 |
| Na^+^/ K^+^of leaves | CK | 0.103±0.008e | 0.107±0.002e | 0.105±0.002e | 0.108±0.002e | 0.110±0.006e | 0.111±0.005d |
|  | 171 | 0.154±0.007d | 0.204±0.009d | 0.232±0.017d | 0.437±0.028d | 0.666±0.054d | 0.915±0.062c |
|  | 342 | 0.315±0.019c | 0.375±0.006c | 0.585±0.040c | 1.095±0.059c | 2.018±0.057c | 3.156±0.134b |
|  | 513 | 0.425±0.021b | 0.618±0.050b | 1.044±0.078b | 2.099±0.143b | 3.578±0.190b | 4.716±0.523a |
|  | 684 | 0.557±0.015a | 1.146±0.039a | 2.012±0.095a | 4.339±0.523a | 8.030±0.342a |  |
| Na^+^/ K^+^ of roots | CK | 0.212±0.002e | 0.208±0.005e | 0.215±0.007e | 0.214±0.003e | 0.214±0.003e | 0.215±0.004d |
|  | 171 | 0.365±0.003d | 0.406±0.020d | 0.516±0.048d | 0.841±0.006d | 1.131±0.008d | 1.389±0.031c |
|  | 342 | 0.648±0.020c | 0.798±0.060c | 1.167±0.054c | 1.614±0.059c | 2.665±0.086c | 3.398±0.150b |
|  | 513 | 0.855±0.058b | 1.088±0.047b | 1.781±0.095b | 2.985±0.170b | 4.390±0.159b | 5.116±0.334a |
|  | 684 | 1.097±0.045a | 1.519±0.113a | 2.634±0.209a | 4.516±0.144a | 8.572±0.306a |  |
| **Note:** The number after ± is the standard deviation.Different small letters in the same column meant significant difference at 0.05 level among treatments. | | | | | | | |

**Table S6** Changes of Na^+^/ Ca^2+^ in roots and leaves under salt stress **(**data in Fig.2B)

| Indicators | NaCl Concentration(mm) | The processing time of NaCl concentration(Days) | | | | | |
| --- | --- | --- | --- | --- | --- | --- | --- |
|  |  | 1 | 3 | 5 | 8 | 11 | 15 |
| Na^+^/ Ca^2+^ of leaves | CK | 0.730±0.015e | 0.734±0.024e | 0.736±0.016e | 0.746±0.031e | 0.754±0.041e | 0.773±0.058d |
|  | 171 | 1.127±0.033d | 1.449±0.110d | 1.961±0.039d | 3.057±0.249d | 4.168±0.071d | 6.237±0.114c |
|  | 342 | 1.694±0.158c | 2.743±0.081c | 3.993±0.241c | 6.505±0.318c | 9.147±0.326c | 14.354±0.571b |
|  | 513 | 2.171±0.085b | 4.557±0.323b | 6.663±0.251b | 10.380±0.490b | 13.860±0.817b | 25.855±2.602a |
|  | 684 | 3.040±0.067a | 8.060±0.315a | 10.243±0.465a | 16.708±0.892a | 31.349±0.731a |  |
| Na^+^/ Ca^2+^ of roots | CK | 2.814±0.086e | 2.952±0.093e | 2.844±0.100e | 2.931±0.093e | 2.932±0.083e | 3.240±0.132d |
|  | 171 | 4.994±0.185d | 5.716±0.226d | 7.515±0.760d | 11.807±0.389d | 15.278±0.119d | 22.594±1.479c |
|  | 342 | 7.716±0.197c | 10.798±0.346c | 15.147±0.043c | 24.835±0.821c | 33.351±1.334c | 51.678±1.086b |
|  | 513 | 11.693±0.947b | 18.706±0.764b | 24.542±0.764b | 38.915±1.624b | 50.720±3.853b | 80.740±4.239a |
|  | 684 | 16.339±0.332a | 27.969±1.170a | 34.441±0.803a | 54.089±2.078a | 101.710±9.624a |  |
| **Note:** The number after ± is the standard deviation.Different small letters in the same column meant significant difference at 0.05 level among treatments. | | | | | | | |

**Table S7** Changes of Na^+^ in roots and leaves under salt stress **(**data in Fig.2C)

| Indicators (mg/g) | NaCl Concentration(mm) | The processing time of NaCl concentration(Days) | | | | | |
| --- | --- | --- | --- | --- | --- | --- | --- |
|  |  | 1 | 3 | 5 | 8 | 11 | 15 |
| Na+ of leaves | CK | 4.052±0.159e | 4.038±0.118e | 4.045±0.020e | 4.076±0.209e | 4.161±0.147e | 4.246±0.118d |
|  | 171 | 6.206±0.132d | 7.634±0.459d | 8.586±0.184d | 12.052±0.780d | 14.885±0.918d | 18.795±0.906c |
|  | 342 | 8.920±0.499c | 9.807±0.630c | 13.942±0.684c | 21.049±0.833c | 26.066±0.689c | 33.184±2.790b |
|  | 513 | 11.229±0.449b | 15.028±1.293b | 21.411±0.881b | 28.842±0.931b | 34.227±1.420b | 39.972±3.709a |
|  | 684 | 14.500±1.450a | 24.655±0.973a | 31.249±0.778a | 43.365±2.276a | 54.004±2.312a |  |
| Na+ of roots | CK | 7.541±0.045e | 7.615±0.009e | 7.629±0.032e | 7.750±0.057e | 7.839±0.062e | 7.989±0.067d |
|  | 171 | 13.013±0.760d | 14.841±0.580d | 18.654±1.675d | 23.084±2.851d | 23.716±0.437d | 25.766±0.880c |
|  | 342 | 20.312±0.868c | 22.096±2.061c | 26.367±1.512c | 30.073±1.690c | 31.080±0.935c | 33.248±1.120b |
|  | 513 | 24.502±1.204b | 26.759±1.292b | 30.011±1.354b | 33.713±0.859b | 34.491±1.423b | 37.588±2.227a |
|  | 684 | 28.690±0.434a | 34.796±0.851a | 36.985±2.364a | 42.423±1.015a | 56.461±2.671a |  |
| **Note:** The number after ± is the standard deviation.Different small letters in the same column meant significant difference at 0.05 level among treatments. | | | | | | | |

**Table S8** Correlation between Na^+^/ K^+^ and Na^+^/ Ca^2+^ in leaves under salt stress **(**data in Fig.2D)

| Indicators | NaCl Concentration(mm) | The processing time of NaCl concentration(Days) | | | | | |
| --- | --- | --- | --- | --- | --- | --- | --- |
|  |  | 1 | 3 | 5 | 8 | 11 | 15 |
| Na^+^/ K^+^of leaves | CK | 0.103±0.008e | 0.107±0.002e | 0.105±0.002e | 0.108±0.002e | 0.110±0.006e | 0.111±0.005d |
|  | 171 | 0.154±0.007d | 0.204±0.009d | 0.232±0.017d | 0.437±0.028d | 0.666±0.054d | 0.915±0.062c |
|  | 342 | 0.315±0.019c | 0.375±0.006c | 0.585±0.040c | 1.095±0.059c | 2.018±0.057c | 3.156±0.134b |
|  | 513 | 0.425±0.021b | 0.618±0.050b | 1.044±0.078b | 2.099±0.143b | 3.578±0.190b | 4.716±0.523a |
|  | 684 | 0.557±0.015a | 1.146±0.039a | 2.012±0.095a | 4.339±0.523a | 8.030±0.342a |  |
| Na^+^/ Ca^2+^ of leaves | CK | 0.730±0.015e | 0.734±0.024e | 0.736±0.016e | 0.746±0.031e | 0.754±0.041e | 0.773±0.058d |
|  | 171 | 1.127±0.033d | 1.449±0.110d | 1.961±0.039d | 3.057±0.249d | 4.168±0.071d | 6.237±0.114c |
|  | 342 | 1.694±0.158c | 2.743±0.081c | 3.993±0.241c | 6.505±0.318c | 9.147±0.326c | 14.354±0.571b |
|  | 513 | 2.171±0.085b | 4.557±0.323b | 6.663±0.251b | 10.380±0.490b | 13.860±0.817b | 25.855±2.602a |
|  | 684 | 3.040±0.067a | 8.060±0.315a | 10.243±0.465a | 16.708±0.892a | 31.349±0.731a |  |
| **Note:** The number after ± is the standard deviation.Different small letters in the same column meant significant difference at 0.05 level among treatments. | | | | | | | |

**Table S9** Correlation between Na^+^ in leaves and roots under salt stress **(**data in Fig.2E)

| Indicators (mg/g)) | NaCl Concentration(mm) | The processing time of NaCl concentration(Days) | | | | | |
| --- | --- | --- | --- | --- | --- | --- | --- |
|  |  | 1 | 3 | 5 | 8 | 11 | 15 |
| Na^+^ of leaves (mg/g) | CK | 4.052±0.159e | 4.038±0.118e | 4.045±0.020e | 4.076±0.209e | 4.161±0.147e | 4.246±0.118d |
|  | 171 | 6.206±0.132d | 7.634±0.459d | 8.586±0.184d | 12.052±0.780d | 14.885±0.918d | 18.795±0.906c |
|  | 342 | 8.920±0.499c | 9.807±0.630c | 13.942±0.684c | 21.049±0.833c | 26.066±0.689c | 33.184±2.790b |
|  | 513 | 11.229±0.449b | 15.028±1.293b | 21.411±0.881b | 28.842±0.931b | 34.227±1.420b | 39.972±3.709a |
|  | 684 | 14.500±1.450a | 24.655±0.973a | 31.249±0.778a | 43.365±2.276a | 54.004±2.312a |  |
| Na^+^ of roots (mg/g) | CK | 7.541±0.045e | 7.615±0.009e | 7.629±0.032e | 7.750±0.057e | 7.839±0.062e | 7.989±0.067d |
|  | 171 | 13.013±0.760d | 14.841±0.580d | 18.654±1.675d | 23.084±2.851d | 23.716±0.437d | 25.766±0.880c |
|  | 342 | 20.312±0.868c | 22.096±2.061c | 26.367±1.512c | 30.073±1.690c | 31.080±0.935c | 33.248±1.120b |
|  | 513 | 24.502±1.204b | 26.759±1.292b | 30.011±1.354b | 33.713±0.859b | 34.491±1.423b | 37.588±2.227a |
|  | 684 | 28.690±0.434a | 34.796±0.851a | 36.985±2.364a | 42.423±1.015a | 56.461±2.671a |  |
| Note: The number after ± is the standard deviation.Different small letters in the same column meant significant difference at 0.05 level among treatments. | | | | | | | |

**Table S10** Correlation of Na^+^/ Ca^2+^ and Na^+^/ K^+^ to Chlorophyll Content in the Leaves of *Salix matsudana* under Salt Stress **(**data in Fig.3A)

| Indicators | NaCl Concentration(mm) | The processing time of NaCl concentration(Days) | | | | | |
| --- | --- | --- | --- | --- | --- | --- | --- |
|  |  | 1 | 3 | 5 | 8 | 11 | 15 |
| ChlorophyII content(mg/g) | CK | 1.889±0.050bc | 1.905±0.050b | 1.910±0.054b | 1.970±0.037b | 1.974±0.066a | 1.933±0.044a |
|  | 171 | 2.278±0.089a | 2.322±0.147a | 2.311±0.083a | 2.061±0.036a | 1.960±0.103a | 1.909±0.073a |
|  | 342 | 1.966±0.036b | 1.885±0.076b | 1.866±0.072b | 1.827±0.064c | 1.499±0.081b | 0.715±0.039b |
|  | 513 | 1.869±0.054c | 1.716±0.054c | 1.414±0.139c | 1.287±0.075d | 0.898±0.029c |  |
|  | 684 | 1.893±0.024bc | 1.450±0.085d | 1.235±0.038d | 1.013±0.044e |  |  |
| Na^+^/ Ca^2+^ of leaves | CK | 0.730±0.015e | 0.734±0.024e | 0.736±0.016e | 0.746±0.031e | 0.754±0.041e | 0.773±0.058d |
|  | 171 | 1.127±0.033d | 1.449±0.110d | 1.961±0.039d | 3.057±0.249d | 4.168±0.071d | 6.237±0.114c |
|  | 342 | 1.694±0.158c | 2.743±0.081c | 3.993±0.241c | 6.505±0.318c | 9.147±0.326c | 14.354±0.571b |
|  | 513 | 2.171±0.085b | 4.557±0.323b | 6.663±0.251b | 10.380±0.490b | 13.860±0.817b | 25.855±2.602a |
|  | 684 | 3.040±0.067a | 8.060±0.315a | 10.243±0.465a | 16.708±0.892a | 31.349±0.731a |  |
| Na^+^/ K^+^of leaves | CK | 0.103±0.008e | 0.107±0.002e | 0.105±0.002e | 0.108±0.002e | 0.110±0.006e | 0.111±0.005d |
|  | 171 | 0.154±0.007d | 0.204±0.009d | 0.232±0.017d | 0.437±0.028d | 0.666±0.054d | 0.915±0.062c |
|  | 342 | 0.315±0.019c | 0.375±0.006c | 0.585±0.040c | 1.095±0.059c | 2.018±0.057c | 3.156±0.134b |
|  | 513 | 0.425±0.021b | 0.618±0.050b | 1.044±0.078b | 2.099±0.143b | 3.578±0.190b | 4.716±0.523a |
|  | 684 | 0.557±0.015a | 1.146±0.039a | 2.012±0.095a | 4.339±0.523a | 8.030±0.342a |  |
| **Note:** The number after ± is the standard deviation.Different small letters in the same column meant significant difference at 0.05 level among treatments. | | | | | | | |

**Table S11** Correlation between Na^+^/Ca^2+^, Na^+^/ K^+^ and PI_ABS_ in the Leaves of *Salix matsudana* under Salt Stress **(**data in Fig.3B)

| Indicators | NaCl Concentration(mm) | The processing time of NaCl concentration(Days) | | | | | |
| --- | --- | --- | --- | --- | --- | --- | --- |
|  |  | 1 | 3 | 5 | 8 | 11 | 15 |
| PI_ABS_ | CK | 4.745±0.283b | 4.629±0.429b | 4.764±0.369a | 4.587±0.499a | 4.736±0.375a | 4.677±0.311a |
|  | 171 | 6.083±0.281a | 5.262±0.400a | 4.547±0.148a | 4.356±0.198a | 4.311±0.291b | 3.286±0.110b |
|  | 342 | 4.569±0.308b | 4.578±0.248b | 4.313±0.292a | 4.128±0.059a | 2.980±0.178c | 1.895±0.100c |
|  | 513 | 3.979±0.178c | 3.084±0.263c | 2.421±0.209b | 2.195±0.089b | 1.029±0.063d |  |
|  | 684 | 3.780±0.262c | 2.239±0.300d | 2.060±0.103b | 1.401±0.010c |  |  |
| Na^+^/ Ca^2+^ of leaves | CK | 0.730±0.015e | 0.734±0.024e | 0.736±0.016e | 0.746±0.031e | 0.754±0.041e | 0.773±0.058d |
|  | 171 | 1.127±0.033d | 1.449±0.110d | 1.961±0.039d | 3.057±0.249d | 4.168±0.071d | 6.237±0.114c |
|  | 342 | 1.694±0.158c | 2.743±0.081c | 3.993±0.241c | 6.505±0.318c | 9.147±0.326c | 14.354±0.571b |
|  | 513 | 2.171±0.085b | 4.557±0.323b | 6.663±0.251b | 10.380±0.490b | 13.860±0.817b | 25.855±2.602a |
|  | 684 | 3.040±0.067a | 8.060±0.315a | 10.243±0.465a | 16.708±0.892a | 31.349±0.731a |  |
| Na^+^/ K^+^of leaves | CK | 0.103±0.008e | 0.107±0.002e | 0.105±0.002e | 0.108±0.002e | 0.110±0.006e | 0.111±0.005d |
|  | 171 | 0.154±0.007d | 0.204±0.009d | 0.232±0.017d | 0.437±0.028d | 0.666±0.054d | 0.915±0.062c |
|  | 342 | 0.315±0.019c | 0.375±0.006c | 0.585±0.040c | 1.095±0.059c | 2.018±0.057c | 3.156±0.134b |
|  | 513 | 0.425±0.021b | 0.618±0.050b | 1.044±0.078b | 2.099±0.143b | 3.578±0.190b | 4.716±0.523a |
|  | 684 | 0.557±0.015a | 1.146±0.039a | 2.012±0.095a | 4.339±0.523a | 8.030±0.342a |  |
| **Note:** The number after ± is the standard deviation.Different small letters in the same column meant significant difference at 0.05 level among treatments. | | | | | | | |

**Table S12** Correlation of Na^+^/Ca^2+^ and Na^+^/K^+^ in the Leaves of *Salix matsudana* with Fv/Fm under Salt Stress. **(**data in Fig.3C)

| Indicators | NaCl Concentration(mm) | The processing time of NaCl concentration(Days) | | | | | |
| --- | --- | --- | --- | --- | --- | --- | --- |
|  |  | 1 | 3 | 5 | 8 | 11 | 15 |
| Fv/Fm | CK | 0.822±0.002ab | 0.826±0.009a | 0.824±0.011a | 0.824±0.014a | 0.820±0.005a | 0.824±0.004a |
|  | 171 | 0.830±0.002a | 0.826±0.008a | 0.811±0.005a | 0.812±0.007a | 0.808±0.001ab | 0.802±0.013b |
|  | 342 | 0.819±0.003b | 0.828±0.005a | 0.803±0.002b | 0.813±0.001a | 0.801±0.005b | 0.772±0.012c |
|  | 513 | 0.816±0.006b | 0.800±0.003b | 0.805±0.004ab | 0.793±0.006b | 0.729±0.017c |  |
|  | 684 | 0.816±0.010b | 0.795±0.015b | 0.790±0.005c | 0.753±0.001c |  |  |
| Na^+^/ Ca^2+^ of leaves | CK | 0.730±0.015e | 0.734±0.024e | 0.736±0.016e | 0.746±0.031e | 0.754±0.041e | 0.773±0.058d |
|  | 171 | 1.127±0.033d | 1.449±0.110d | 1.961±0.039d | 3.057±0.249d | 4.168±0.071d | 6.237±0.114c |
|  | 342 | 1.694±0.158c | 2.743±0.081c | 3.993±0.241c | 6.505±0.318c | 9.147±0.326c | 14.354±0.571b |
|  | 513 | 2.171±0.085b | 4.557±0.323b | 6.663±0.251b | 10.380±0.490b | 13.860±0.817b | 25.855±2.602a |
|  | 684 | 3.040±0.067a | 8.060±0.315a | 10.243±0.465a | 16.708±0.892a | 31.349±0.731a |  |
| Na^+^/ K^+^of leaves | CK | 0.103±0.008e | 0.107±0.002e | 0.105±0.002e | 0.108±0.002e | 0.110±0.006e | 0.111±0.005d |
|  | 171 | 0.154±0.007d | 0.204±0.009d | 0.232±0.017d | 0.437±0.028d | 0.666±0.054d | 0.915±0.062c |
|  | 342 | 0.315±0.019c | 0.375±0.006c | 0.585±0.040c | 1.095±0.059c | 2.018±0.057c | 3.156±0.134b |
|  | 513 | 0.425±0.021b | 0.618±0.050b | 1.044±0.078b | 2.099±0.143b | 3.578±0.190b | 4.716±0.523a |
|  | 684 | 0.557±0.015a | 1.146±0.039a | 2.012±0.095a | 4.339±0.523a | 8.030±0.342a |  |
| **Note:** The number after ± is the standard deviation.Different small letters in the same column meant significant difference at 0.05 level among treatments. | | | | | | | |

**Table S13** Correlation between Na^+^ and chlorophyll content in *salix matsulosa* leaves under salt stress **(**data in Fig.3D)

| Indicators (mg/g) | NaCl Concentration(mm) | The processing time of NaCl concentration(Days) | | | | | |
| --- | --- | --- | --- | --- | --- | --- | --- |
|  |  | 1 | 3 | 5 | 8 | 11 | 15 |
| Na^+^ of leaves (mg/g) | CK | 4.052±0.159e | 4.038±0.118e | 4.045±0.020e | 4.076±0.209e | 4.161±0.147e | 4.246±0.118d |
|  | 171 | 6.206±0.132d | 7.634±0.459d | 8.586±0.184d | 12.052±0.780d | 14.885±0.918d | 18.795±0.906c |
|  | 342 | 8.920±0.499c | 9.807±0.630c | 13.942±0.684c | 21.049±0.833c | 26.066±0.689c | 33.184±2.790b |
|  | 513 | 11.229±0.449b | 15.028±1.293b | 21.411±0.881b | 28.842±0.931b | 34.227±1.420b | 39.972±3.709a |
|  | 684 | 14.500±1.450a | 24.655±0.973a | 31.249±0.778a | 43.365±2.276a | 54.004±2.312a |  |
| ChlorophyII content(mg/g) | CK | 1.889±0.050bc | 1.905±0.050b | 1.910±0.054b | 1.970±0.037b | 1.974±0.066a | 1.933±0.044a |
|  | 171 | 2.278±0.089a | 2.322±0.147a | 2.311±0.083a | 2.061±0.036a | 1.960±0.103a | 1.909±0.073a |
|  | 342 | 1.966±0.036b | 1.885±0.076b | 1.866±0.072b | 1.827±0.064c | 1.499±0.081b | 0.715±0.039b |
|  | 513 | 1.869±0.054c | 1.716±0.054c | 1.414±0.139c | 1.287±0.075d | 0.898±0.029c |  |
|  | 684 | 1.893±0.024bc | 1.450±0.085d | 1.235±0.038d | 1.013±0.044e |  |  |
| **Note:** The number after ± is the standard deviation.Different small letters in the same column meant significant difference at 0.05 level among treatments. | | | | | | | |

**Table S14** Correlation between Na^+^ and PI_ABS_ in s*alix matsulosa* leaves under salt stress **(**data in Fig.3E)

| Indicators | NaCl Concentration(mm) | The processing time of NaCl concentration(Days) | | | | | |
| --- | --- | --- | --- | --- | --- | --- | --- |
|  |  | 1 | 3 | 5 | 8 | 11 | 15 |
| PI_ABS_ | CK | 4.745±0.283b | 4.629±0.429b | 4.764±0.369a | 4.587±0.499a | 4.736±0.375a | 4.677±0.311a |
|  | 171 | 6.083±0.281a | 5.262±0.400a | 4.547±0.148a | 4.356±0.198a | 4.311±0.291b | 3.286±0.110b |
|  | 342 | 4.569±0.308b | 4.578±0.248b | 4.313±0.292a | 4.128±0.059a | 2.980±0.178c | 1.895±0.100c |
|  | 513 | 3.979±0.178c | 3.084±0.263c | 2.421±0.209b | 2.195±0.089b | 1.029±0.063d |  |
|  | 684 | 3.780±0.262c | 2.239±0.300d | 2.060±0.103b | 1.401±0.010c |  |  |
| Na^+^ of leaves (mg/g) | CK | 4.052±0.159e | 4.038±0.118e | 4.045±0.020e | 4.076±0.209e | 4.161±0.147e | 4.246±0.118d |
|  | 171 | 6.206±0.132d | 7.634±0.459d | 8.586±0.184d | 12.052±0.780d | 14.885±0.918d | 18.795±0.906c |
|  | 342 | 8.920±0.499c | 9.807±0.630c | 13.942±0.684c | 21.049±0.833c | 26.066±0.689c | 33.184±2.790b |
|  | 513 | 11.229±0.449b | 15.028±1.293b | 21.411±0.881b | 28.842±0.931b | 34.227±1.420b | 39.972±3.709a |
|  | 684 | 14.500±1.450a | 24.655±0.973a | 31.249±0.778a | 43.365±2.276a | 54.004±2.312a |  |
| **Note:** The number after ± is the standard deviation.Different small letters in the same column meant significant difference at 0.05 level among treatments. | | | | | | | |

**Table S15** Correlation between Na^+^ and Fv/Fm in *salix matsulosa* leaves under salt stress **(**data in Fig.3F)

| Indicators | NaCl Concentration(mm) | The processing time of NaCl concentration(Days) | | | | | |
| --- | --- | --- | --- | --- | --- | --- | --- |
|  |  | 1 | 3 | 5 | 8 | 11 | 15 |
| Fv/Fm | CK | 0.822±0.002ab | 0.826±0.009a | 0.824±0.011a | 0.824±0.014a | 0.820±0.005a | 0.824±0.004a |
|  | 171 | 0.830±0.002a | 0.826±0.008a | 0.811±0.005a | 0.812±0.007a | 0.808±0.001ab | 0.802±0.013b |
|  | 342 | 0.819±0.003b | 0.828±0.005a | 0.803±0.002b | 0.813±0.001a | 0.801±0.005b | 0.772±0.012c |
|  | 513 | 0.816±0.006b | 0.800±0.003b | 0.805±0.004ab | 0.793±0.006b | 0.729±0.017c |  |
|  | 684 | 0.816±0.010b | 0.795±0.015b | 0.790±0.005c | 0.753±0.001c |  |  |
| Na^+^ of leaves (mg/g) | CK | 4.052±0.159e | 4.038±0.118e | 4.045±0.020e | 4.076±0.209e | 4.161±0.147e | 4.246±0.118d |
|  | 171 | 6.206±0.132d | 7.634±0.459d | 8.586±0.184d | 12.052±0.780d | 14.885±0.918d | 18.795±0.906c |
|  | 342 | 8.920±0.499c | 9.807±0.630c | 13.942±0.684c | 21.049±0.833c | 26.066±0.689c | 33.184±2.790b |
|  | 513 | 11.229±0.449b | 15.028±1.293b | 21.411±0.881b | 28.842±0.931b | 34.227±1.420b | 39.972±3.709a |
|  | 684 | 14.500±1.450a | 24.655±0.973a | 31.249±0.778a | 43.365±2.276a | 54.004±2.312a |  |
| **Note:** The number after ± is the standard deviation.Different small letters in the same column meant significant difference at 0.05 level among treatments. | | | | | | | |

**Table S16** Correlation of Na^+^/ Ca^2+^ and Na^+^/ K^+^ in the Leaves of *Salix matsudana* to Pn under Salt Stress **(**data in Fig.4A)

| Indicators | NaCl Concentration(mm) | The processing time of NaCl concentration(Days) | | | | | |
| --- | --- | --- | --- | --- | --- | --- | --- |
|  |  | 1 | 3 | 5 | 8 | 11 | 15 |
| Pn(µmol m⁻² s⁻¹) | CK | 11.74±0.68b | 13.81±0.77a | 12.98±0.66a | 10.07±0.97a | 9.95±0.33a | 9.94±0.44a |
|  | 171 | 13.08±0.42a | 14.66±0.64a | 13.68±0.51a | 8.23±0.21b | 7.79±0.20b | 7.98±0.16b |
|  | 342 | 11.17±0.51b | 11.24±0.62b | 9.54±0.19b | 6.16±0.44c | 5.52±0.29c | 5.27±0.08c |
|  | 513 | 11.66±0.45b | 8.28±0.46c | 7.61±0.26c | 3.26±0.05d | 1.44±0.08d |  |
|  | 684 | 8.26±0.37c | 6.62±0.20d | 3.64±0.12d | 1.55±0.06e |  |  |
| Na^+^/ Ca^2+^ of leaves | CK | 0.730±0.015e | 0.734±0.024e | 0.736±0.016e | 0.746±0.031e | 0.754±0.041e | 0.773±0.058d |
|  | 171 | 1.127±0.033d | 1.449±0.110d | 1.961±0.039d | 3.057±0.249d | 4.168±0.071d | 6.237±0.114c |
|  | 342 | 1.694±0.158c | 2.743±0.081c | 3.993±0.241c | 6.505±0.318c | 9.147±0.326c | 14.354±0.571b |
|  | 513 | 2.171±0.085b | 4.557±0.323b | 6.663±0.251b | 10.380±0.490b | 13.860±0.817b | 25.855±2.602a |
|  | 684 | 3.040±0.067a | 8.060±0.315a | 10.243±0.465a | 16.708±0.892a | 31.349±0.731a |  |
| Na^+^/ K^+^of leaves | CK | 0.103±0.008e | 0.107±0.002e | 0.105±0.002e | 0.108±0.002e | 0.110±0.006e | 0.111±0.005d |
|  | 171 | 0.154±0.007d | 0.204±0.009d | 0.232±0.017d | 0.437±0.028d | 0.666±0.054d | 0.915±0.062c |
|  | 342 | 0.315±0.019c | 0.375±0.006c | 0.585±0.040c | 1.095±0.059c | 2.018±0.057c | 3.156±0.134b |
|  | 513 | 0.425±0.021b | 0.618±0.050b | 1.044±0.078b | 2.099±0.143b | 3.578±0.190b | 4.716±0.523a |
|  | 684 | 0.557±0.015a | 1.146±0.039a | 2.012±0.095a | 4.339±0.523a | 8.030±0.342a |  |
| **Note:** The number after ± is the standard deviation.Different small letters in the same column meant significant difference at 0.05 level among treatments. | | | | | | | |

**Table S17** Correlation of Na^+^/ Ca^2+^ and Na^+^/ K^+^ in the Leaves of *Salix matsudana* to E under Salt Stress **(**data in Fig.4B)

| Indicators | NaCl Concentration(mm) | The processing time of NaCl concentration(Days) | | | | | |
| --- | --- | --- | --- | --- | --- | --- | --- |
|  |  | 1 | 3 | 5 | 8 | 11 | 15 |
| E(mmol m⁻² s⁻¹) | CK | 5.21±0.40ab | 5.31±0.17a | 5.47±0.18a | 5.53±0.35a | 5.50±0.37a | 5.33±0.42a |
|  | 171 | 5.14±0.20ab | 5.37±0.29a | 5.10±0.22a | 3.81±0.31b | 3.52±0.10b | 3.45±0.19b |
|  | 342 | 5.44±0.25a | 4.42±0.15b | 3.29±0.16b | 2.52±0.09c | 1.60±0.03c | 1.62±0.09c |
|  | 513 | 5.39±0.32a | 3.82±0.13c | 1.91±0.08c | 1.80±0.07d | 1.19±0.02d |  |
|  | 684 | 4.82±0.03b | 3.55±0.20c | 1.38±0.06d | 1.00±0.25e |  |  |
| Na^+^/ Ca^2+^ of leaves | CK | 0.730±0.015e | 0.734±0.024e | 0.736±0.016e | 0.746±0.031e | 0.754±0.041e | 0.773±0.058d |
|  | 171 | 1.127±0.033d | 1.449±0.110d | 1.961±0.039d | 3.057±0.249d | 4.168±0.071d | 6.237±0.114c |
|  | 342 | 1.694±0.158c | 2.743±0.081c | 3.993±0.241c | 6.505±0.318c | 9.147±0.326c | 14.354±0.571b |
|  | 513 | 2.171±0.085b | 4.557±0.323b | 6.663±0.251b | 10.380±0.490b | 13.860±0.817b | 25.855±2.602a |
|  | 684 | 3.040±0.067a | 8.060±0.315a | 10.243±0.465a | 16.708±0.892a | 31.349±0.731a |  |
| Na^+^/ K^+^of leaves | CK | 0.103±0.008e | 0.107±0.002e | 0.105±0.002e | 0.108±0.002e | 0.110±0.006e | 0.111±0.005d |
|  | 171 | 0.154±0.007d | 0.204±0.009d | 0.232±0.017d | 0.437±0.028d | 0.666±0.054d | 0.915±0.062c |
|  | 342 | 0.315±0.019c | 0.375±0.006c | 0.585±0.040c | 1.095±0.059c | 2.018±0.057c | 3.156±0.134b |
|  | 513 | 0.425±0.021b | 0.618±0.050b | 1.044±0.078b | 2.099±0.143b | 3.578±0.190b | 4.716±0.523a |
|  | 684 | 0.557±0.015a | 1.146±0.039a | 2.012±0.095a | 4.339±0.523a | 8.030±0.342a |  |
| **Note:** The number after ± is the standard deviation.Different small letters in the same column meant significant difference at 0.05 level among treatments. | | | | | | | |

**Table S18** Correlation of Na^+^/Ca^2+^ and Na^+^/K^+^ in the Leaves of *Salix matsudana* with Gs under Salt Stress **(**data in Fig.4C)

| Indicators | NaCl Concentration(mm) | The processing time of NaCl concentration(Days) | | | | | |
| --- | --- | --- | --- | --- | --- | --- | --- |
|  |  | 1 | 3 | 5 | 8 | 11 | 15 |
| Gs(mol m⁻² s⁻¹) | CK | 0.16622±0.00910a | 0.17290±0.01114a | 0.17112±0.00954a | 0.16374±0.00979a | 0.16114±0.00842a | 0.16744±0.00999a |
|  | 171 | 0.16990±0.00582a | 0.14212±0.00728b | 0.11465±0.00530b | 0.11952±0.00438b | 0.10544±0.00790b | 0.10669±0.00447b |
|  | 342 | 0.14874±0.01159b | 0.11770±0.00768c | 0.09994±0.00664c | 0.08946±0.00676c | 0.08466±0.00369c | 0.08818±0.00202c |
|  | 513 | 0.12413±0.00749c | 0.09994±0.00668d | 0.07112±0.00328d | 0.06647±0.00485d | 0.05793±0.00163d |  |
|  | 684 | 0.12857±0.00932c | 0.08077±0.00631e | 0.07273±0.00465d | 0.05228±0.00324e |  |  |
| Na^+^/ Ca^2+^ of leaves | CK | 0.730±0.015e | 0.734±0.024e | 0.736±0.016e | 0.746±0.031e | 0.754±0.041e | 0.773±0.058d |
|  | 171 | 1.127±0.033d | 1.449±0.110d | 1.961±0.039d | 3.057±0.249d | 4.168±0.071d | 6.237±0.114c |
|  | 342 | 1.694±0.158c | 2.743±0.081c | 3.993±0.241c | 6.505±0.318c | 9.147±0.326c | 14.354±0.571b |
|  | 513 | 2.171±0.085b | 4.557±0.323b | 6.663±0.251b | 10.380±0.490b | 13.860±0.817b | 25.855±2.602a |
|  | 684 | 3.040±0.067a | 8.060±0.315a | 10.243±0.465a | 16.708±0.892a | 31.349±0.731a |  |
| Na^+^/ K^+^of leaves | CK | 0.103±0.008e | 0.107±0.002e | 0.105±0.002e | 0.108±0.002e | 0.110±0.006e | 0.111±0.005d |
|  | 171 | 0.154±0.007d | 0.204±0.009d | 0.232±0.017d | 0.437±0.028d | 0.666±0.054d | 0.915±0.062c |
|  | 342 | 0.315±0.019c | 0.375±0.006c | 0.585±0.040c | 1.095±0.059c | 2.018±0.057c | 3.156±0.134b |
|  | 513 | 0.425±0.021b | 0.618±0.050b | 1.044±0.078b | 2.099±0.143b | 3.578±0.190b | 4.716±0.523a |
|  | 684 | 0.557±0.015a | 1.146±0.039a | 2.012±0.095a | 4.339±0.523a | 8.030±0.342a |  |
| **Note:** The number after ± is the standard deviation.Different small letters in the same column meant significant difference at 0.05 level among treatments. | | | | | | | |

**Table S19** Correlation of Na^+^/Ca^2+^ and Na^+^/K^+^ in the Leaves of *Salix matsudana* with Ci under Salt Stress **(**data in Fig.4D)

| Indicators | NaCl Concentration(mm) | The processing time of NaCl concentration(Days) | | | | | |
| --- | --- | --- | --- | --- | --- | --- | --- |
|  |  | 1 | 3 | 5 | 8 | 11 | 15 |
| Ci (µmol mol⁻¹) | CK | 294.17±27.41a | 300.67±19.10a | 301.75±13.98ab | 293.75±13.99a | 291.90±13.55a | 295.41±4.34a |
|  | 171 | 294.22±11.81a | 291.13±21.85a | 303.89±8.39a | 288.12±16.49ab | 274.78±5.92ab | 262.64±15.06b |
|  | 342 | 299.38±12.32a | 293.93±9.18a | 284.33±12.16bc | 277.55±16.73bc | 262.17±21.00b | 243.78±13.15c |
|  | 513 | 290.35±10.20a | 288.92±18.10a | 275.94±8.02c | 275.82±8.08bc | 265.23±17.36b |  |
|  | 684 | 281.30±6.13a | 285.26±11.22a | 273.45±12.50c | 267.75±9.19c |  |  |
| Na^+^/ Ca^2+^ of leaves | CK | 0.730±0.015e | 0.734±0.024e | 0.736±0.016e | 0.746±0.031e | 0.754±0.041e | 0.773±0.058d |
|  | 171 | 1.127±0.033d | 1.449±0.110d | 1.961±0.039d | 3.057±0.249d | 4.168±0.071d | 6.237±0.114c |
|  | 342 | 1.694±0.158c | 2.743±0.081c | 3.993±0.241c | 6.505±0.318c | 9.147±0.326c | 14.354±0.571b |
|  | 513 | 2.171±0.085b | 4.557±0.323b | 6.663±0.251b | 10.380±0.490b | 13.860±0.817b | 25.855±2.602a |
|  | 684 | 3.040±0.067a | 8.060±0.315a | 10.243±0.465a | 16.708±0.892a | 31.349±0.731a |  |
| Na^+^/ K^+^of leaves | CK | 0.103±0.008e | 0.107±0.002e | 0.105±0.002e | 0.108±0.002e | 0.110±0.006e | 0.111±0.005d |
|  | 171 | 0.154±0.007d | 0.204±0.009d | 0.232±0.017d | 0.437±0.028d | 0.666±0.054d | 0.915±0.062c |
|  | 342 | 0.315±0.019c | 0.375±0.006c | 0.585±0.040c | 1.095±0.059c | 2.018±0.057c | 3.156±0.134b |
|  | 513 | 0.425±0.021b | 0.618±0.050b | 1.044±0.078b | 2.099±0.143b | 3.578±0.190b | 4.716±0.523a |
|  | 684 | 0.557±0.015a | 1.146±0.039a | 2.012±0.095a | 4.339±0.523a | 8.030±0.342a |  |
| **Note:** The number after ± is the standard deviation.Different small letters in the same column meant significant difference at 0.05 level among treatments. | | | | | | | |

**Table S20** Correlation of Na^+^ and Pn in the Leaves of *Salix matsudana* under Salt Stress **(**data in Fig.4E)

| Indicators | NaCl Concentration(mm) | The processing time of NaCl concentration(Days) | | | | | |
| --- | --- | --- | --- | --- | --- | --- | --- |
|  |  | 1 | 3 | 5 | 8 | 11 | 15 |
| Na^+^ of leaves (mg/g) | CK | 4.052±0.159e | 4.038±0.118e | 4.045±0.020e | 4.076±0.209e | 4.161±0.147e | 4.246±0.118d |
|  | 171 | 6.206±0.132d | 7.634±0.459d | 8.586±0.184d | 12.052±0.780d | 14.885±0.918d | 18.795±0.906c |
|  | 342 | 8.920±0.499c | 9.807±0.630c | 13.942±0.684c | 21.049±0.833c | 26.066±0.689c | 33.184±2.790b |
|  | 513 | 11.229±0.449b | 15.028±1.293b | 21.411±0.881b | 28.842±0.931b | 34.227±1.420b | 39.972±3.709a |
|  | 684 | 14.500±1.450a | 24.655±0.973a | 31.249±0.778a | 43.365±2.276a | 54.004±2.312a |  |
| Pn(µmol m⁻² s⁻¹) | CK | 11.74±0.68b | 13.81±0.77a | 12.98±0.66a | 10.07±0.97a | 9.95±0.33a | 9.94±0.44a |
|  | 171 | 13.08±0.42a | 14.66±0.64a | 13.68±0.51a | 8.23±0.21b | 7.79±0.20b | 7.98±0.16b |
|  | 342 | 11.17±0.51b | 11.24±0.62b | 9.54±0.19b | 6.16±0.44c | 5.52±0.29c | 5.27±0.08c |
|  | 513 | 11.66±0.45b | 8.28±0.46c | 7.61±0.26c | 3.26±0.05d | 1.44±0.08d |  |
|  | 684 | 8.26±0.37c | 6.62±0.20d | 3.64±0.12d | 1.55±0.06e |  |  |
| **Note:** The number after ± is the standard deviation.Different small letters in the same column meant significant difference at 0.05 level among treatments. | | | | | | | |

**Table S21** Correlation of Na^+^ in the Leaves of *Salix matsudana* to E under Salt Stress. **(**data in Fig.4F)

| Indicators | NaCl Concentration(mm) | The processing time of NaCl concentration(Days) | | | | | |
| --- | --- | --- | --- | --- | --- | --- | --- |
|  |  | 1 | 3 | 5 | 8 | 11 | 15 |
| E(mmol m⁻² s⁻¹) | CK | 5.21±0.40ab | 5.31±0.17a | 5.47±0.18a | 5.53±0.35a | 5.50±0.37a | 5.33±0.42a |
|  | 171 | 5.14±0.20ab | 5.37±0.29a | 5.10±0.22a | 3.81±0.31b | 3.52±0.10b | 3.45±0.19b |
|  | 342 | 5.44±0.25a | 4.42±0.15b | 3.29±0.16b | 2.52±0.09c | 1.60±0.03c | 1.62±0.09c |
|  | 513 | 5.39±0.32a | 3.82±0.13c | 1.91±0.08c | 1.80±0.07d | 1.19±0.02d |  |
|  | 684 | 4.82±0.03b | 3.55±0.20c | 1.38±0.06d | 1.00±0.25e |  |  |
| Na^+^ of leaves (mg/g) | CK | 4.052±0.159e | 4.038±0.118e | 4.045±0.020e | 4.076±0.209e | 4.161±0.147e | 4.246±0.118d |
|  | 171 | 6.206±0.132d | 7.634±0.459d | 8.586±0.184d | 12.052±0.780d | 14.885±0.918d | 18.795±0.906c |
|  | 342 | 8.920±0.499c | 9.807±0.630c | 13.942±0.684c | 21.049±0.833c | 26.066±0.689c | 33.184±2.790b |
|  | 513 | 11.229±0.449b | 15.028±1.293b | 21.411±0.881b | 28.842±0.931b | 34.227±1.420b | 39.972±3.709a |
|  | 684 | 14.500±1.450a | 24.655±0.973a | 31.249±0.778a | 43.365±2.276a | 54.004±2.312a |  |
| **Note:** The number after ± is the standard deviation.Different small letters in the same column meant significant difference at 0.05 level among treatments. | | | | | | | |

**Table S22** Correlation of Na^+^ in the Leaves of *Salix matsudana* with Gs under Salt Stress(data in Fig.4G)

| Indicators | NaCl Concentration(mm) | The processing time of NaCl concentration(Days) | | | | | |
| --- | --- | --- | --- | --- | --- | --- | --- |
|  |  | 1 | 3 | 5 | 8 | 11 | 15 |
| Gs(mmol m⁻² s⁻¹) | CK | 0.16622±0.00910a | 0.17290±0.01114a | 0.17112±0.00954a | 0.16374±0.00979a | 0.16114±0.00842a | 0.16744±0.00999a |
|  | 171 | 0.16990±0.00582a | 0.14212±0.00728b | 0.11465±0.00530b | 0.11952±0.00438b | 0.10544±0.00790b | 0.10669±0.00447b |
|  | 342 | 0.14874±0.01159b | 0.11770±0.00768c | 0.09994±0.00664c | 0.08946±0.00676c | 0.08466±0.00369c | 0.08818±0.00202c |
|  | 513 | 0.12413±0.00749c | 0.09994±0.00668d | 0.07112±0.00328d | 0.06647±0.00485d | 0.05793±0.00163d |  |
|  | 684 | 0.12857±0.00932c | 0.08077±0.00631e | 0.07273±0.00465d | 0.05228±0.00324e |  |  |
| Na^+^ of leaves (mg/g) | CK | 4.052±0.159e | 4.038±0.118e | 4.045±0.020e | 4.076±0.209e | 4.161±0.147e | 4.246±0.118d |
|  | 171 | 6.206±0.132d | 7.634±0.459d | 8.586±0.184d | 12.052±0.780d | 14.885±0.918d | 18.795±0.906c |
|  | 342 | 8.920±0.499c | 9.807±0.630c | 13.942±0.684c | 21.049±0.833c | 26.066±0.689c | 33.184±2.790b |
|  | 513 | 11.229±0.449b | 15.028±1.293b | 21.411±0.881b | 28.842±0.931b | 34.227±1.420b | 39.972±3.709a |
|  | 684 | 14.500±1.450a | 24.655±0.973a | 31.249±0.778a | 43.365±2.276a | 54.004±2.312a |  |
| **Note:** The number after ± is the standard deviation.Different small letters in the same column meant significant difference at 0.05 level among treatments. | | | | | | | |

**Table S23** Correlation of Na^+^ in the Leaves of *Salix matsudana* with Ci under Salt Stress **(**data in Fig.4H)

| Indicators | NaCl Concentration(mm) | The processing time of NaCl concentration(Days) | | | | | |
| --- | --- | --- | --- | --- | --- | --- | --- |
|  |  | 1 | 3 | 5 | 8 | 11 | 15 |
| Ci (µmol mol⁻¹) | CK | 294.17±27.41a | 300.67±19.10a | 301.75±13.98ab | 293.75±13.99a | 291.90±13.55a | 295.41±4.34a |
|  | 171 | 294.22±11.81a | 291.13±21.85a | 303.89±8.39a | 288.12±16.49ab | 274.78±5.92ab | 262.64±15.06b |
|  | 342 | 299.38±12.32a | 293.93±9.18a | 284.33±12.16bc | 277.55±16.73bc | 262.17±21.00b | 243.78±13.15c |
|  | 513 | 290.35±10.20a | 288.92±18.10a | 275.94±8.02c | 275.82±8.08bc | 265.23±17.36b |  |
|  | 684 | 281.30±6.13a | 285.26±11.22a | 273.45±12.50c | 267.75±9.19c |  |  |
| Na^+^ of leaves (mg/g) | CK | 4.052±0.159e | 4.038±0.118e | 4.045±0.020e | 4.076±0.209e | 4.161±0.147e | 4.246±0.118d |
|  | 171 | 6.206±0.132d | 7.634±0.459d | 8.586±0.184d | 12.052±0.780d | 14.885±0.918d | 18.795±0.906c |
|  | 342 | 8.920±0.499c | 9.807±0.630c | 13.942±0.684c | 21.049±0.833c | 26.066±0.689c | 33.184±2.790b |
|  | 513 | 11.229±0.449b | 15.028±1.293b | 21.411±0.881b | 28.842±0.931b | 34.227±1.420b | 39.972±3.709a |
|  | 684 | 14.500±1.450a | 24.655±0.973a | 31.249±0.778a | 43.365±2.276a | 54.004±2.312a |  |
| **Note:** The number after ± is the standard deviation.Different small letters in the same column meant significant difference at 0.05 level among treatments. | | | | | | | |


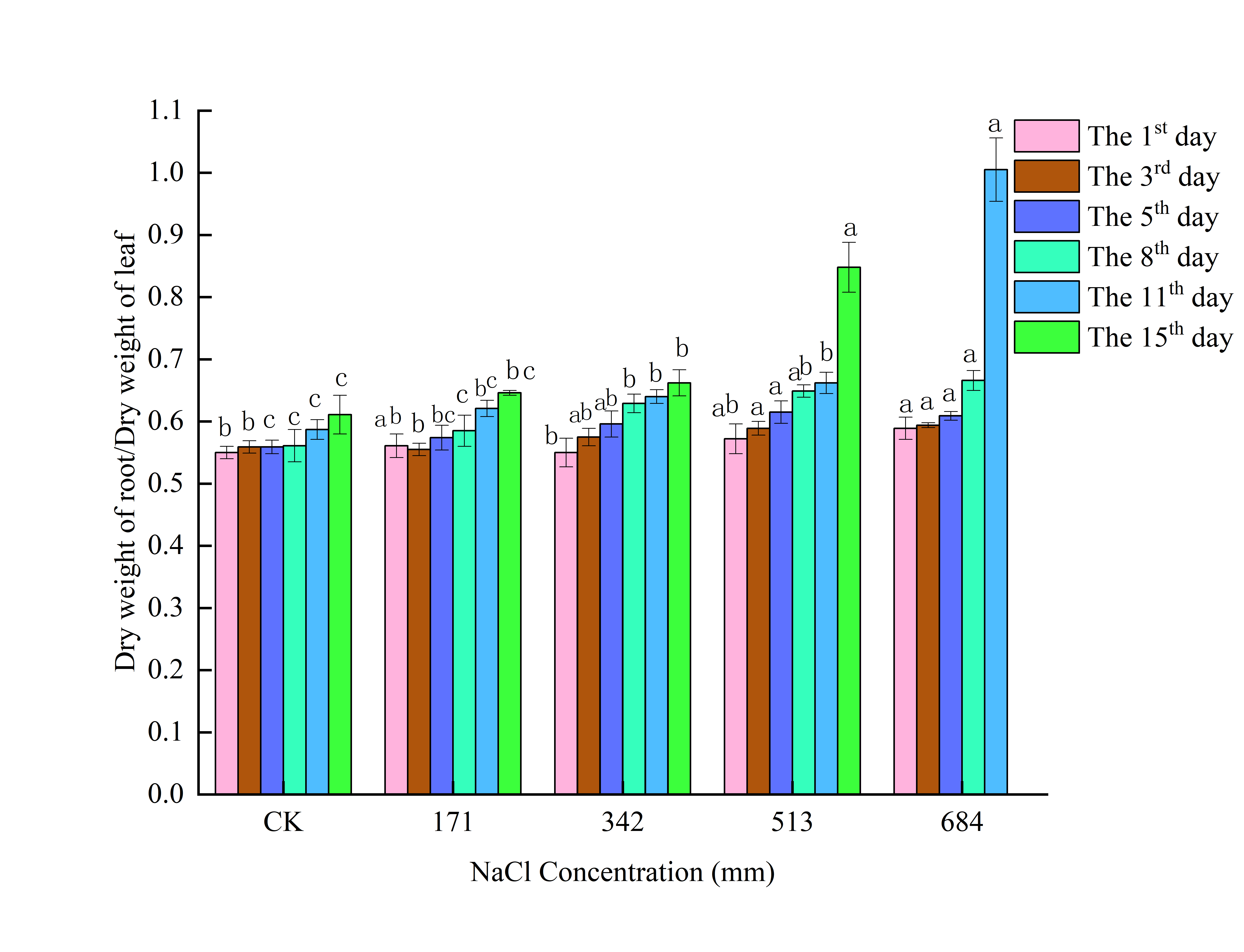


**Fig. S1** Effects of salt stress on ratio of roots and leaves dry weight of *Salix matsudana.* (data in Fig.1A)

**
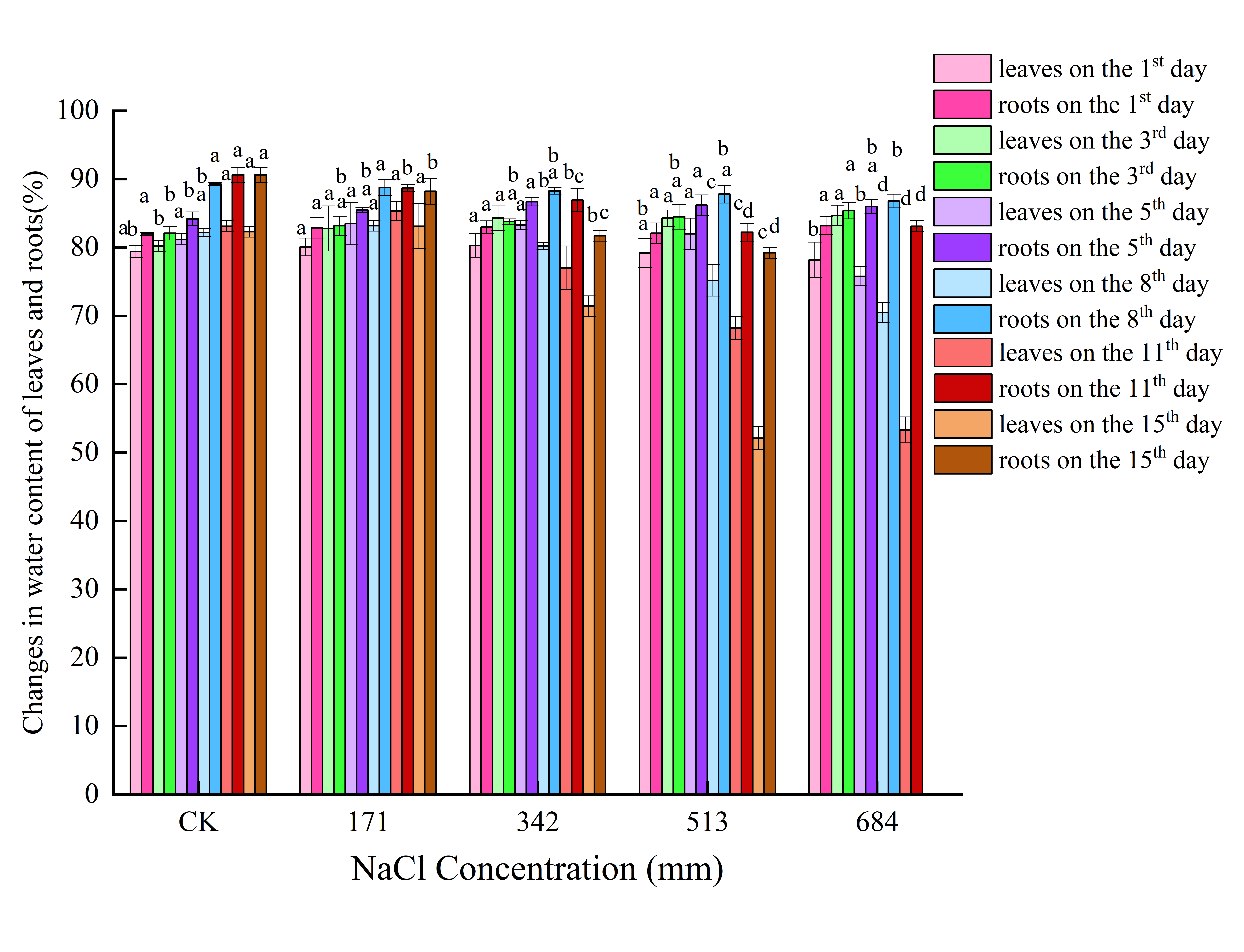
**

**Fig. S2** Effects of salt stress on water content of roots and leaves of *Salix matsulosa.* (data in Fig.1B)

**
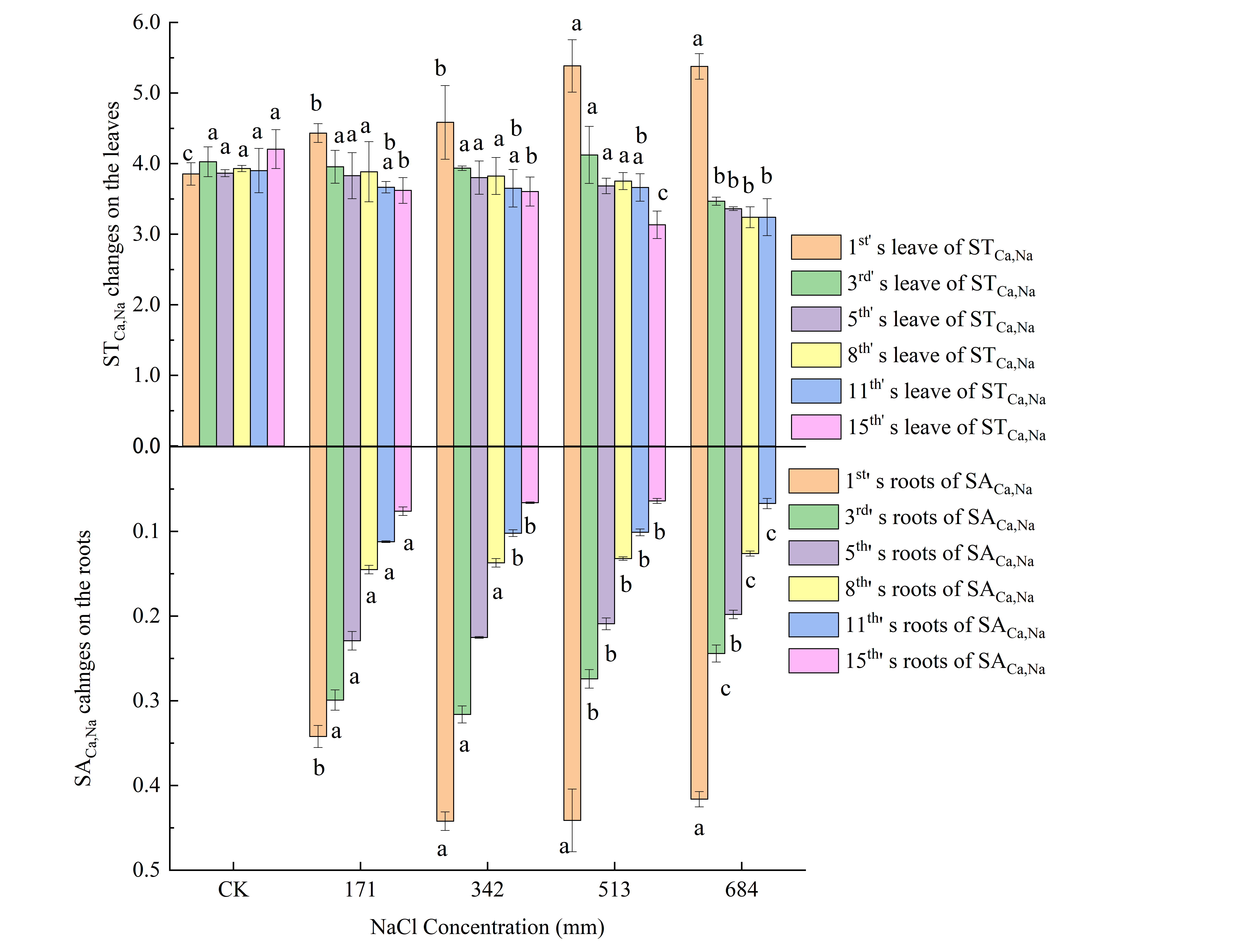
**

**Fig. S3** Effect of salt stress on selective uptake and transportation of Ca^2+^ in roots and leaves of *Salix matsudana* (data in Fig.1C )

**
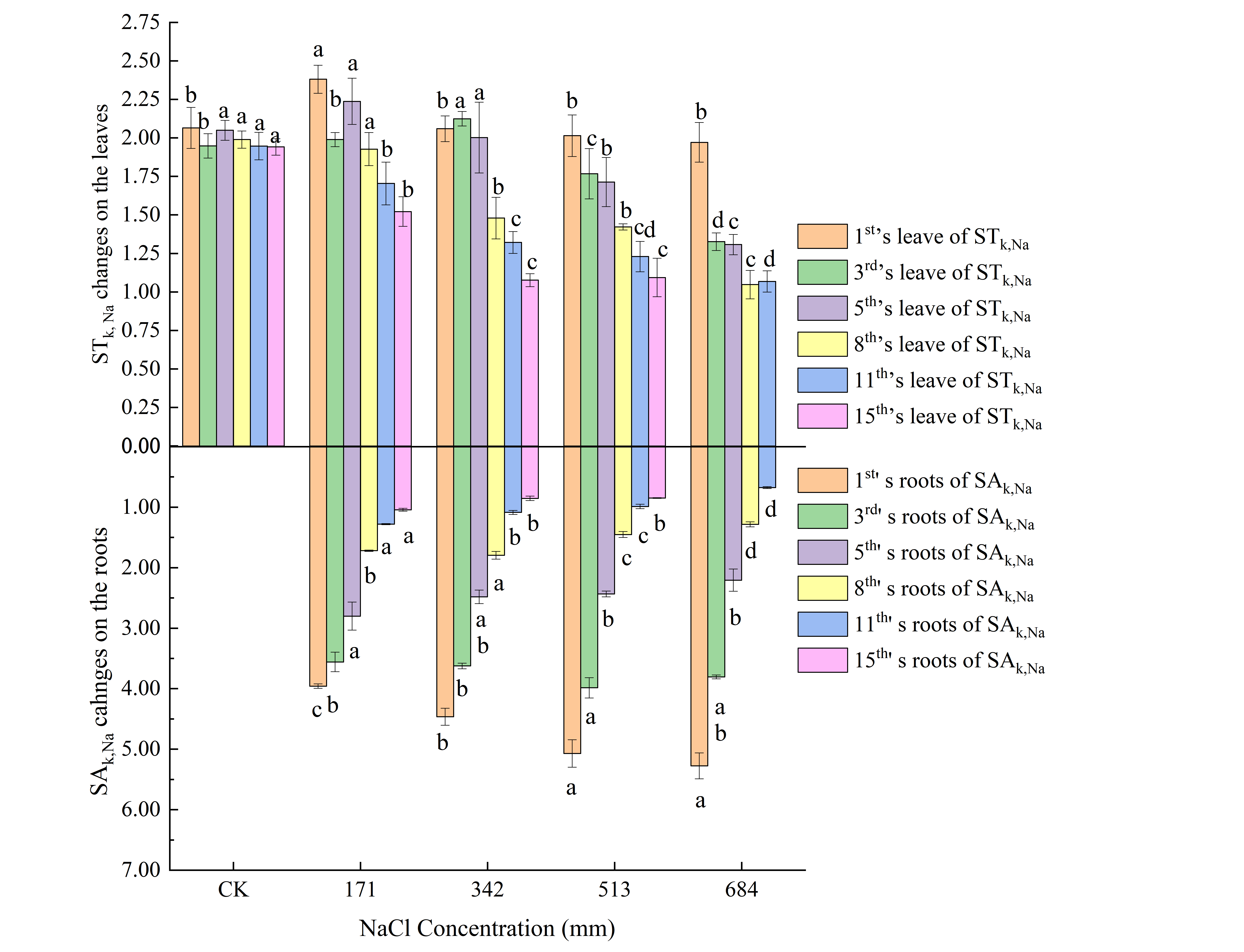
**

**Fig. S4** Effect of salt stress on selective uptake and transportation of K^+^ in roots and leaves of *Salix matsudana* (data in Fig.1D )

**
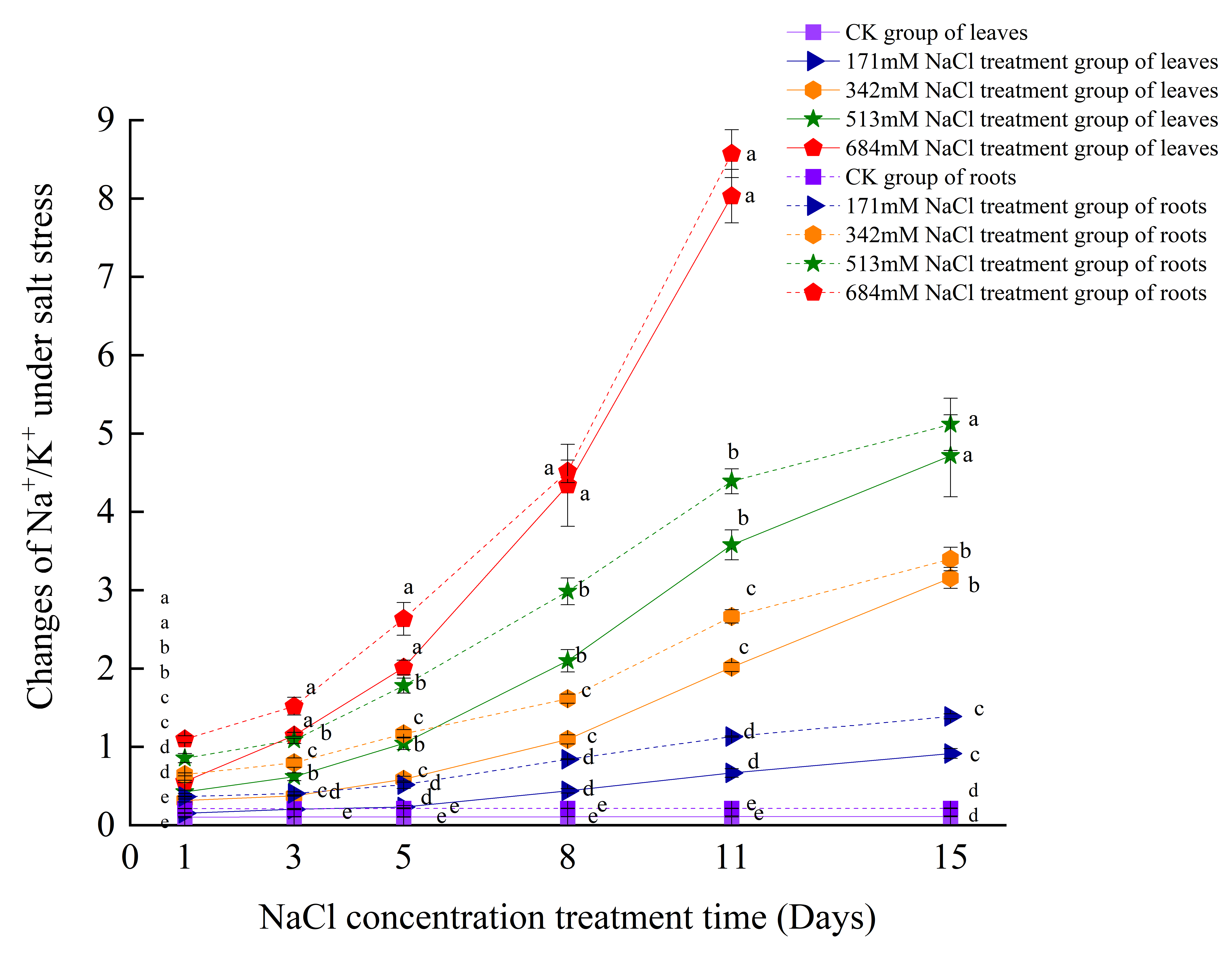
**

**Fig. S5** Changes of Na^+^/ K^+^ in roots and leaves under salt stress(data in Fig.2A )

**
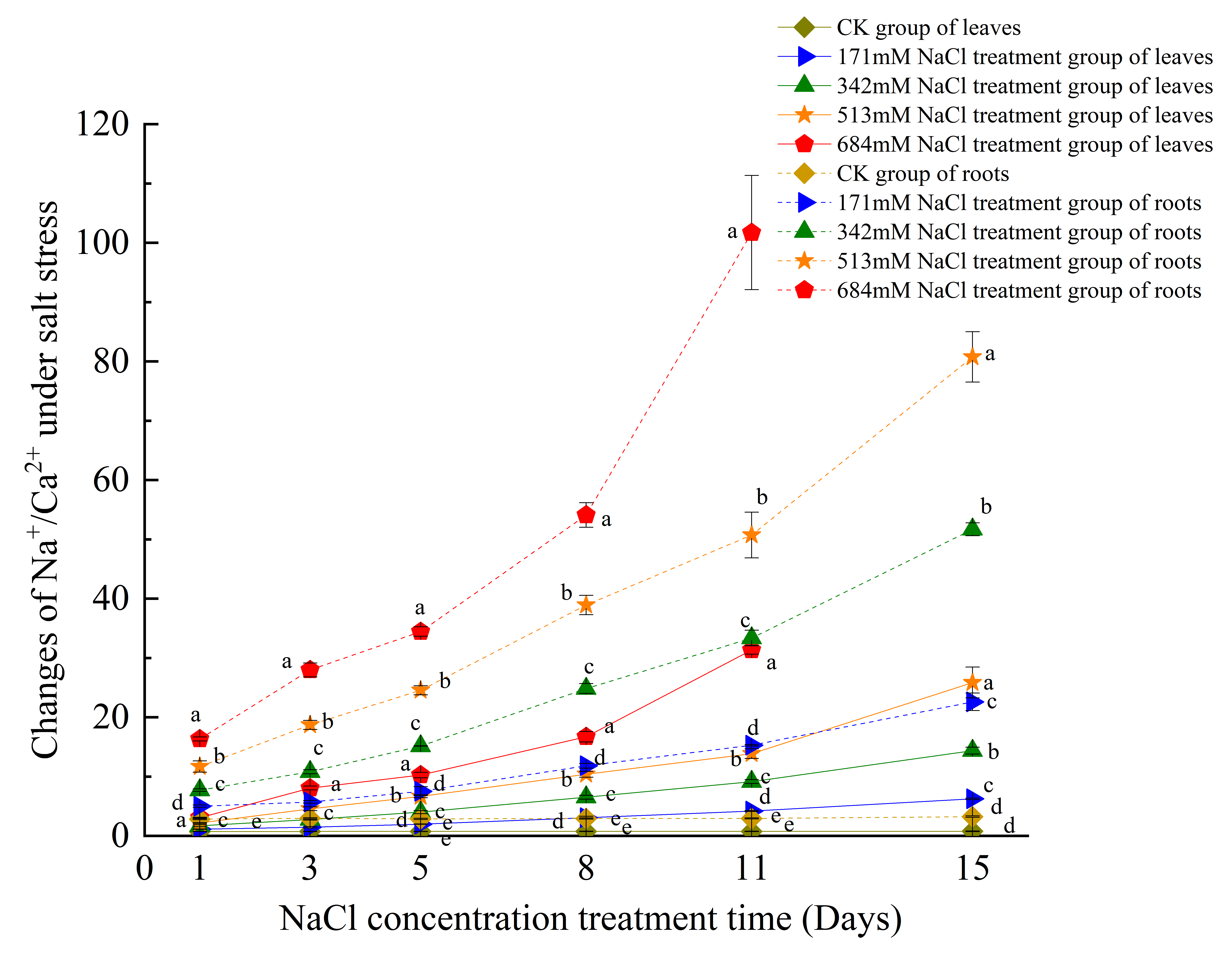
**

**Fig. S6** Changes of Na^+^/ Ca^2+^ in roots and leaves under salt stress(data in Fig.2B )

**
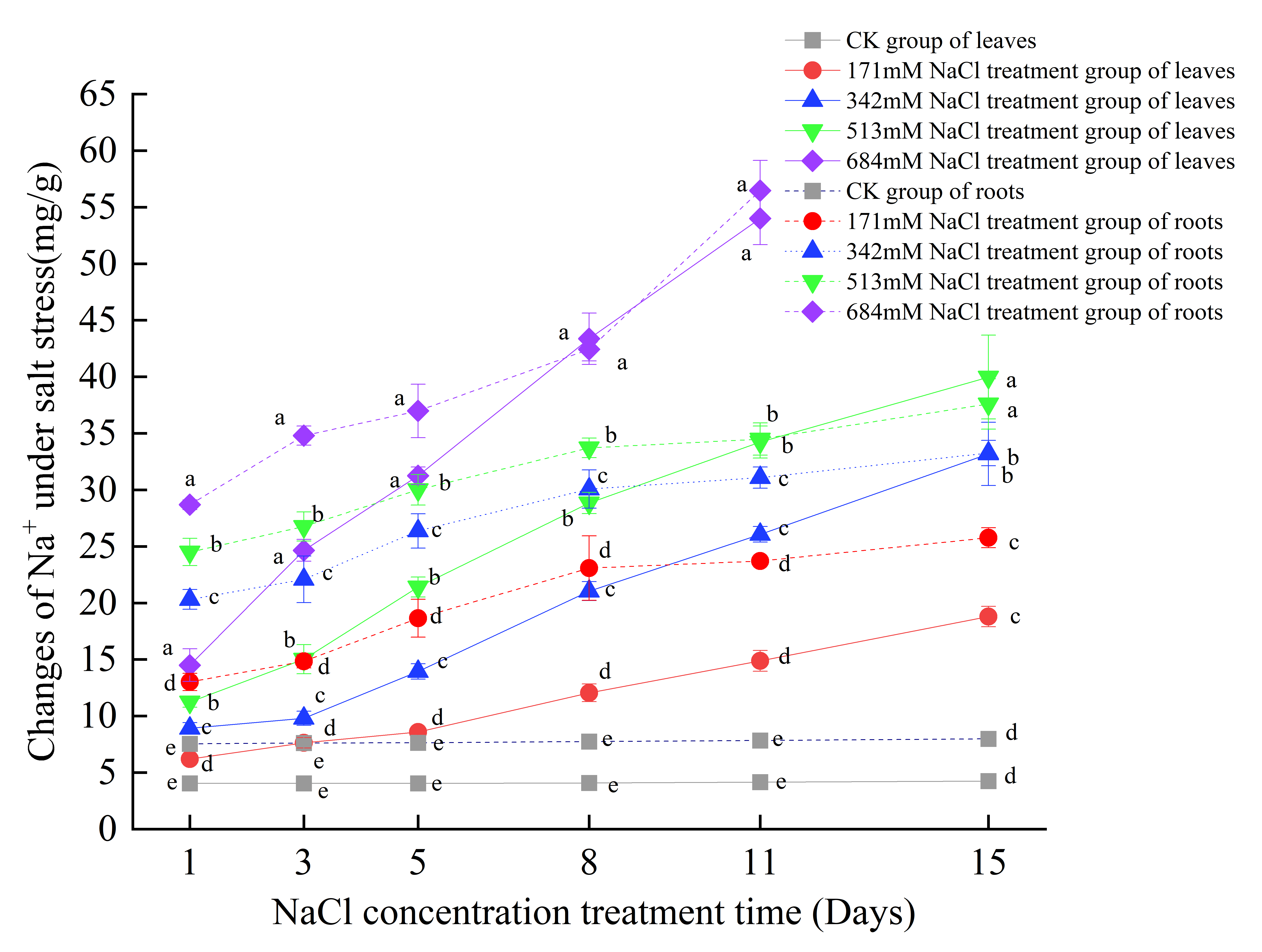
**

**Fig. S7** Changes of Na^+^ in roots and leaves under salt stress (data in Fig.2C )

**
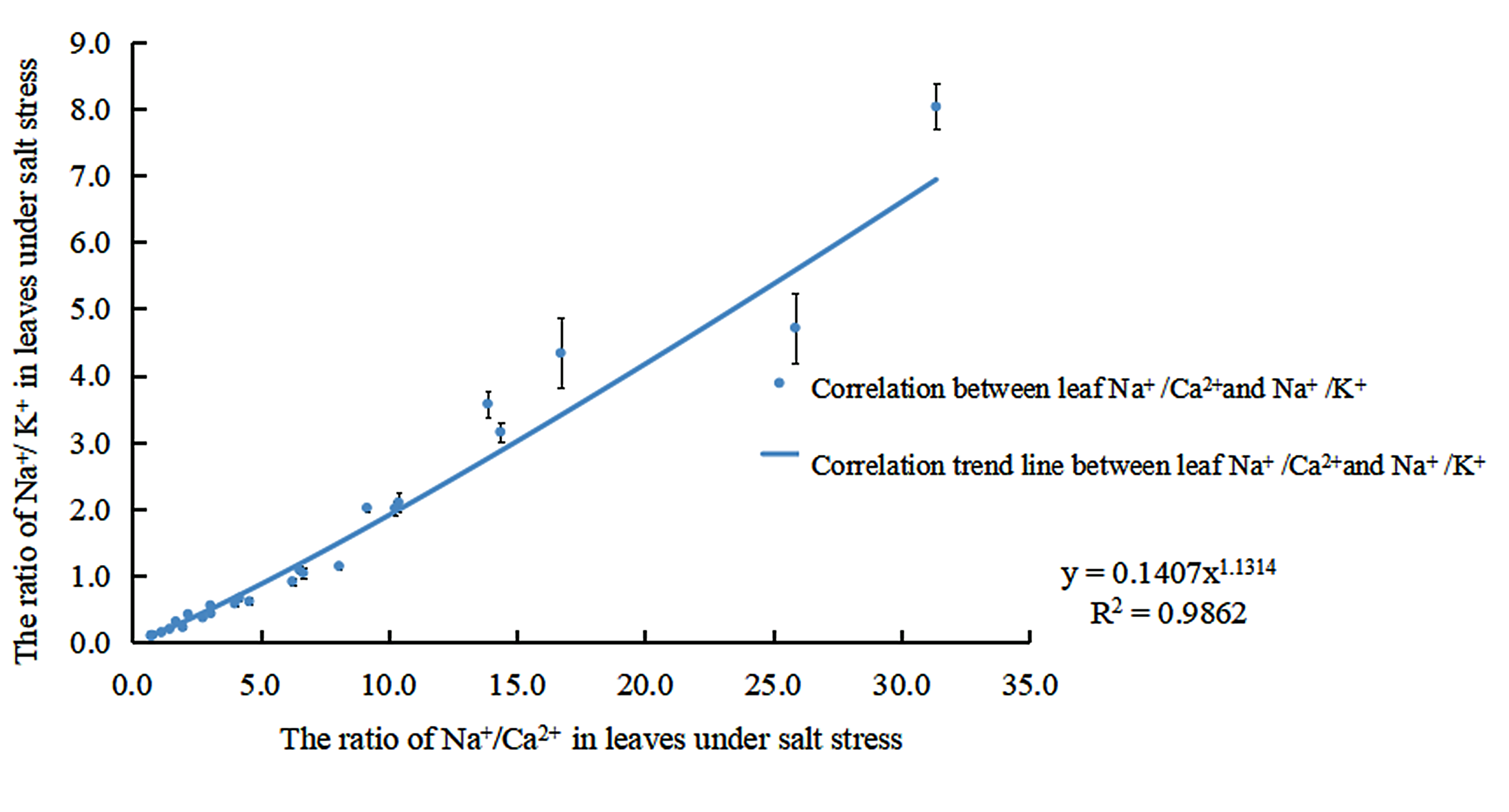
**

**Fig. S8** Correlation between Na^+^/ K^+^ and Na^+^/ Ca^2+^ in leaves under salt stress (data in Fig.2D )

**
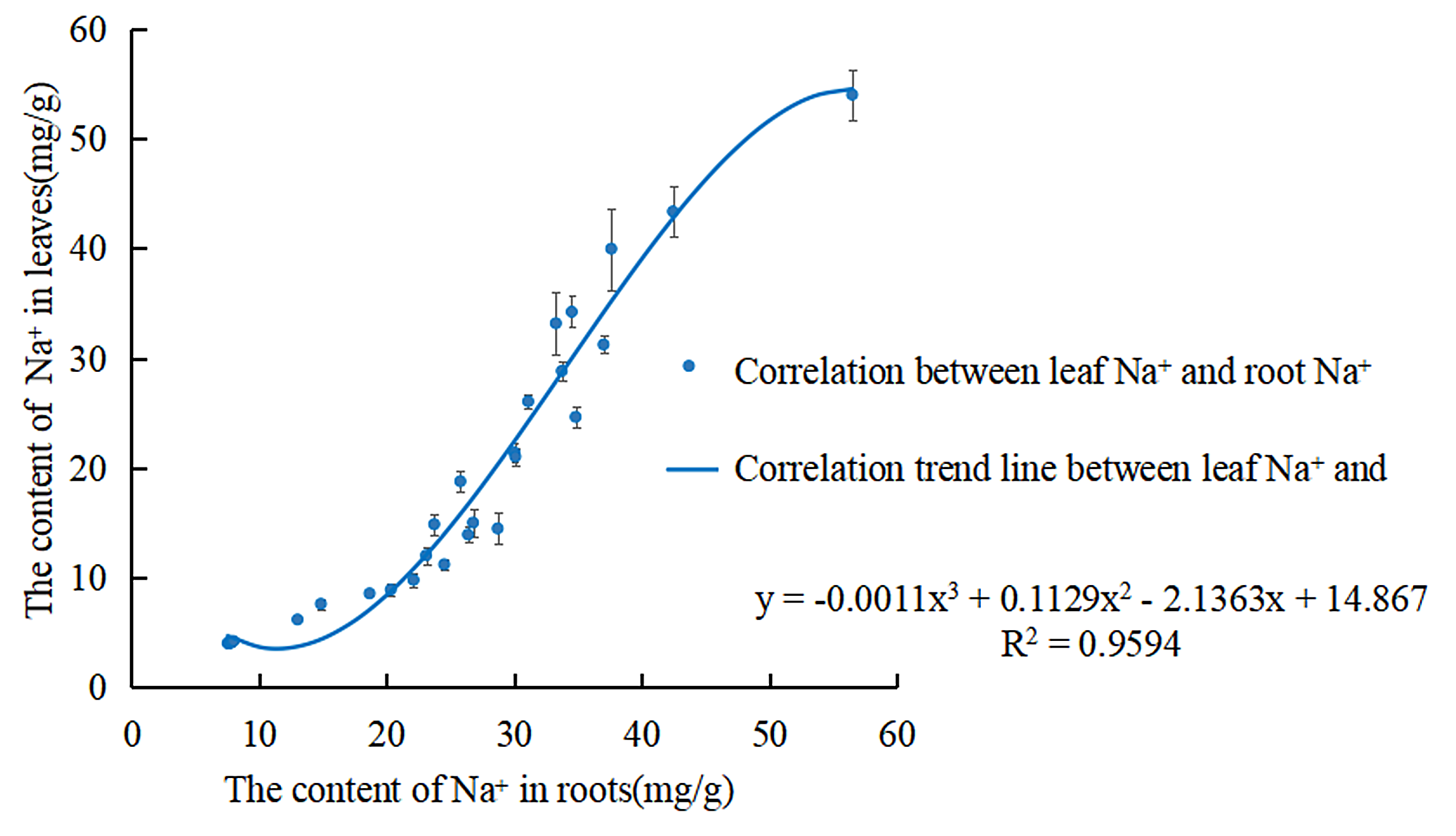
**

**Fig. S9** Correlation between Na^+^ in leaves and roots under salt stress (data in Fig.2E )


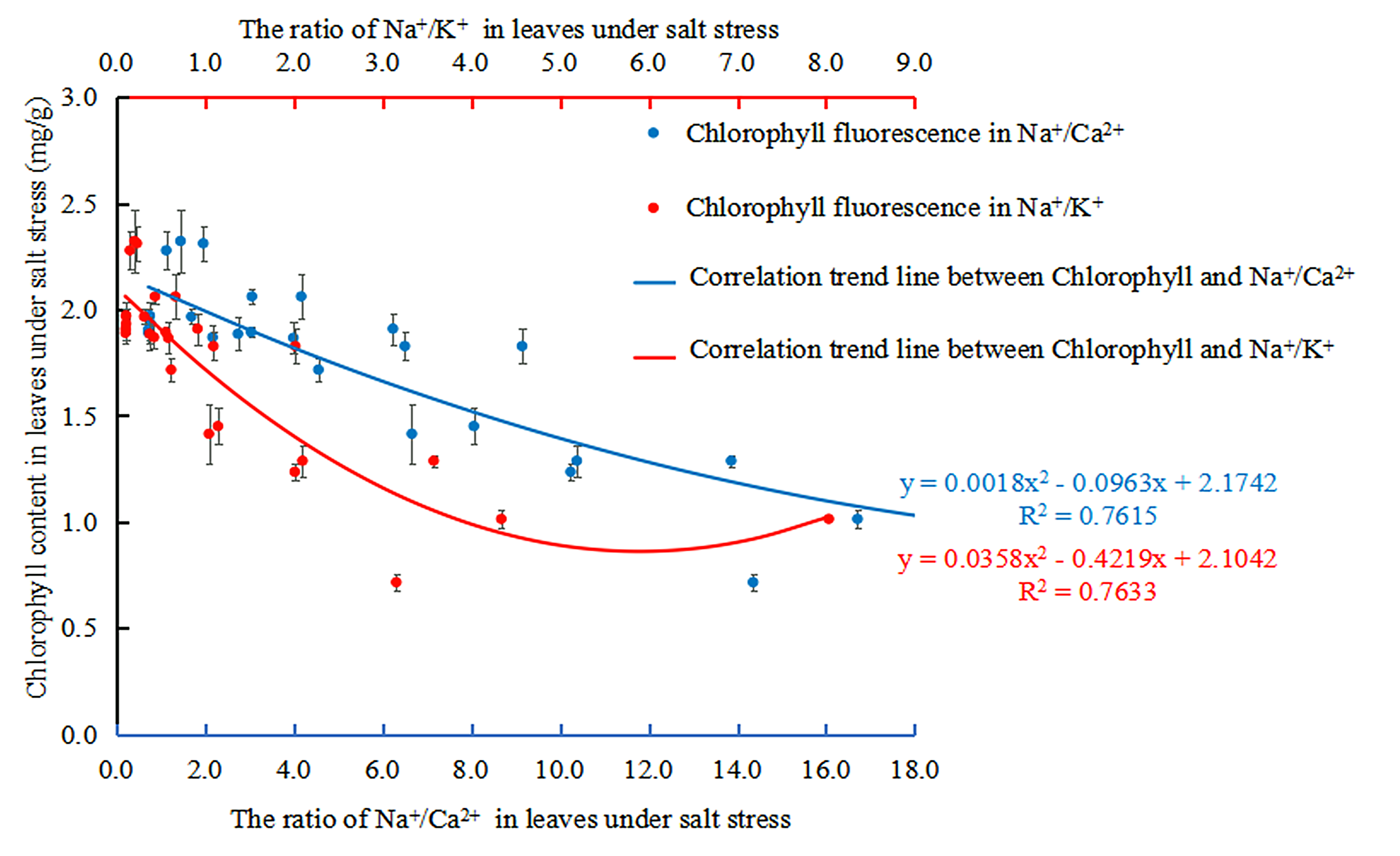


**Fig. S10** Correlation of Na^+^/ Ca^2+^ and Na^+^/ K^+^ to Chlorophyll Content in the Leaves of *Salix matsudana* under Salt Stress (data in Fig.3A )


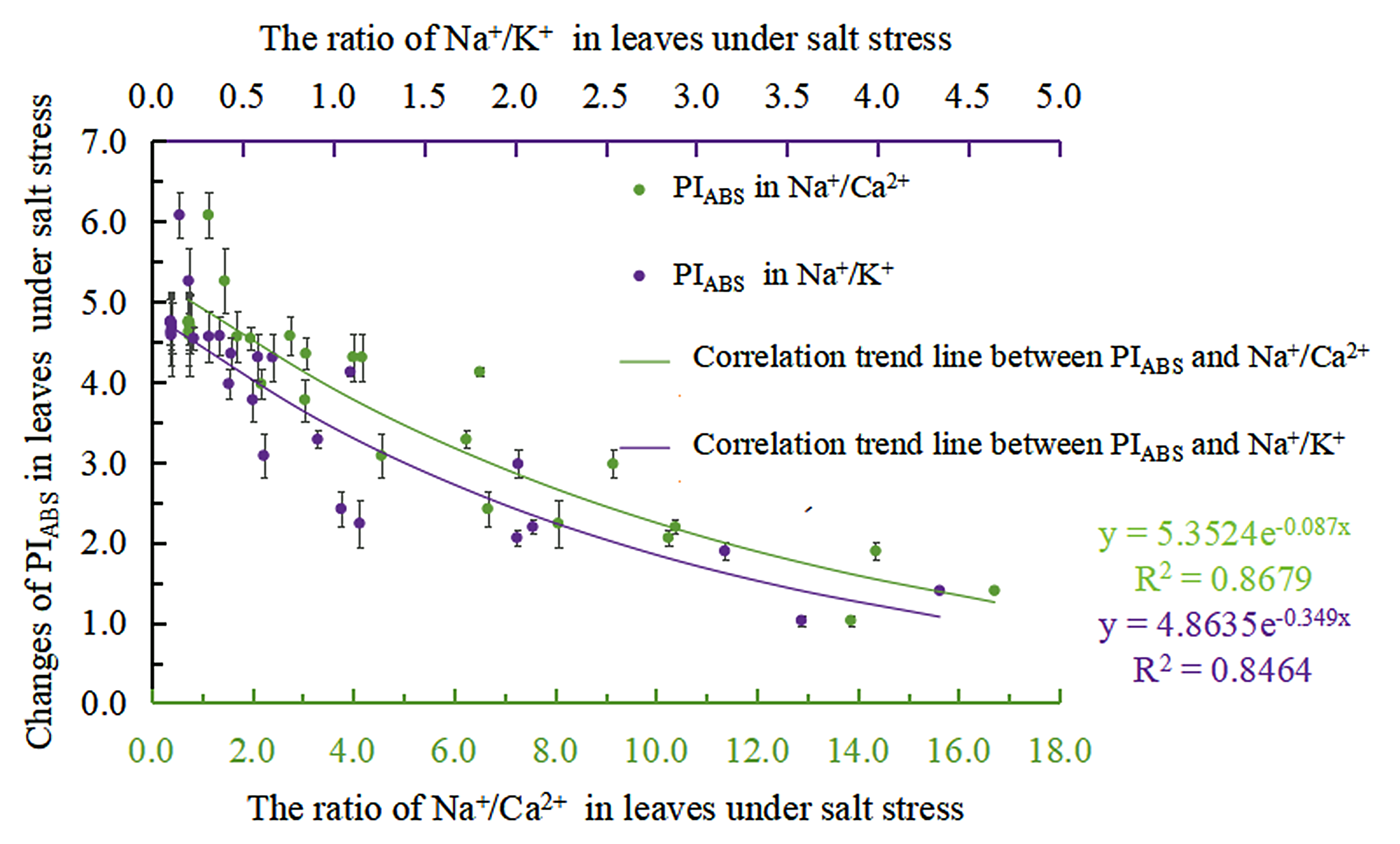


**Fig. S11** Correlation between Na^+^/Ca^2+^, Na^+^/ K^+^ and PI_ABS_ in the Leaves of *Salix matsudana* under Salt Stress (data in Fig.3B )


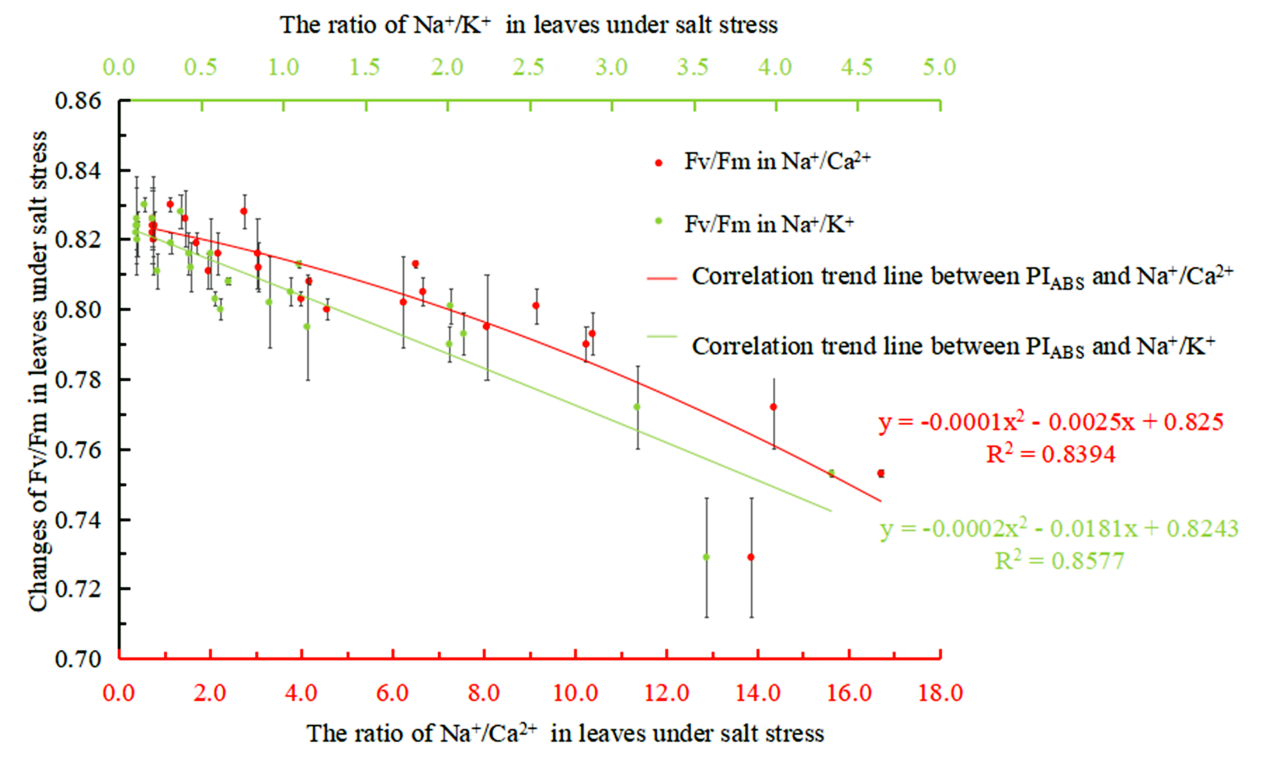


**Fig. S12** Correlation of Na^+^/Ca^2+^ and Na^+^/K^+^ in the Leaves of *Salix matsudana* with Fv/Fm under Salt Stress (data in Fig.3C )


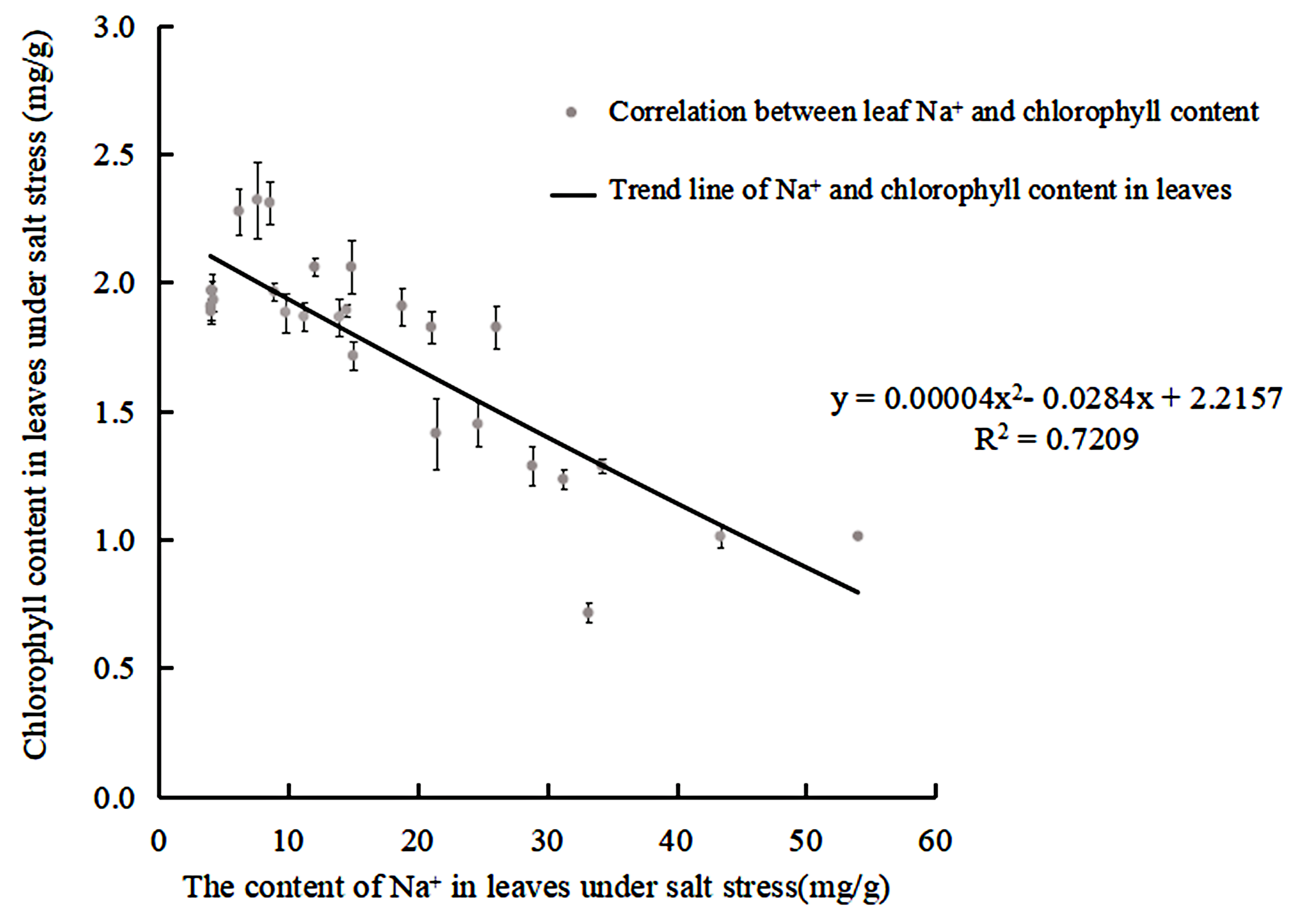


**Fig. S13** Correlation between Na^+^ and chlorophyll content in *salix matsulosa* leaves under salt stress (data in Fig.3D )


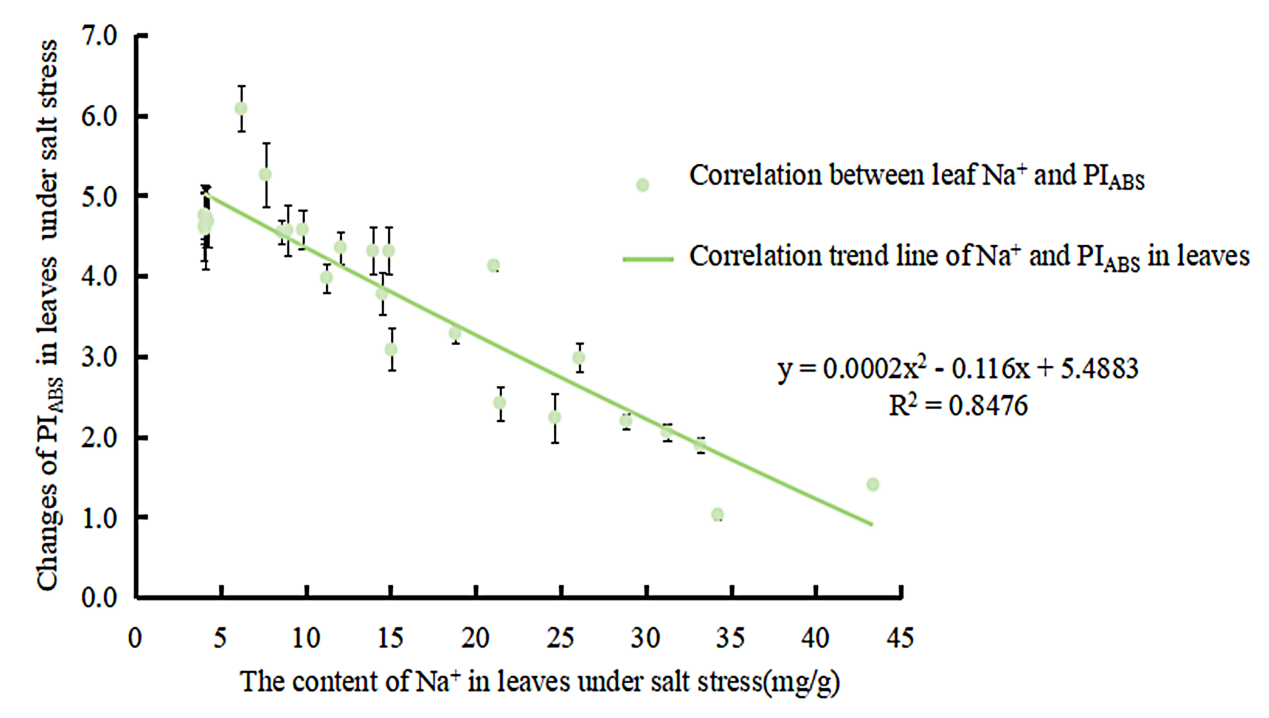


**Fig. S14** Correlation between Na^+^ and PI_ABS_ in *salix matsulosa* leaves under salt stress (data in Fig.3E)


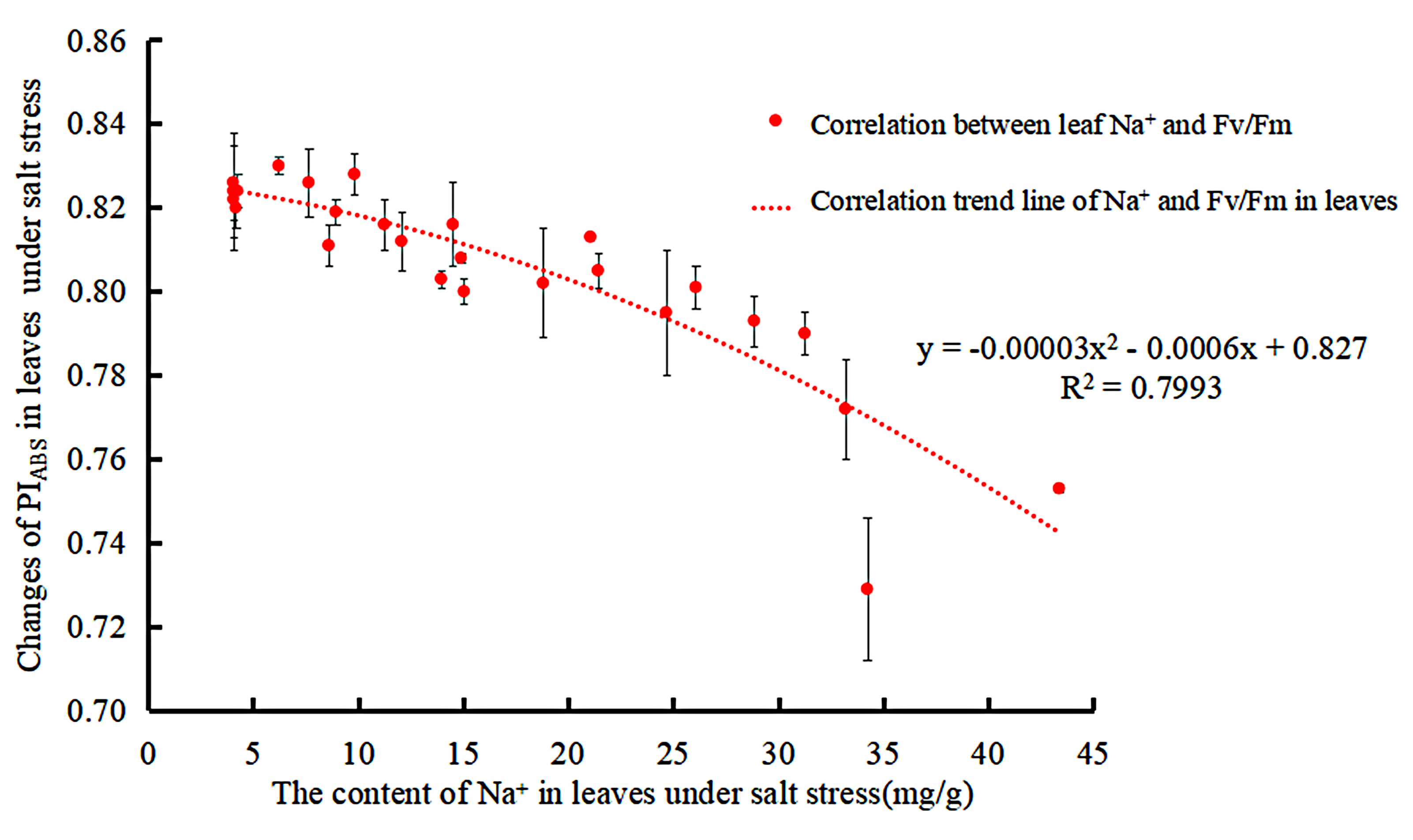


**Fig. S15** Correlation between Na^+^ and Fv/Fm in *salix matsulosa* leaves under salt stress (data in Fig.3F)


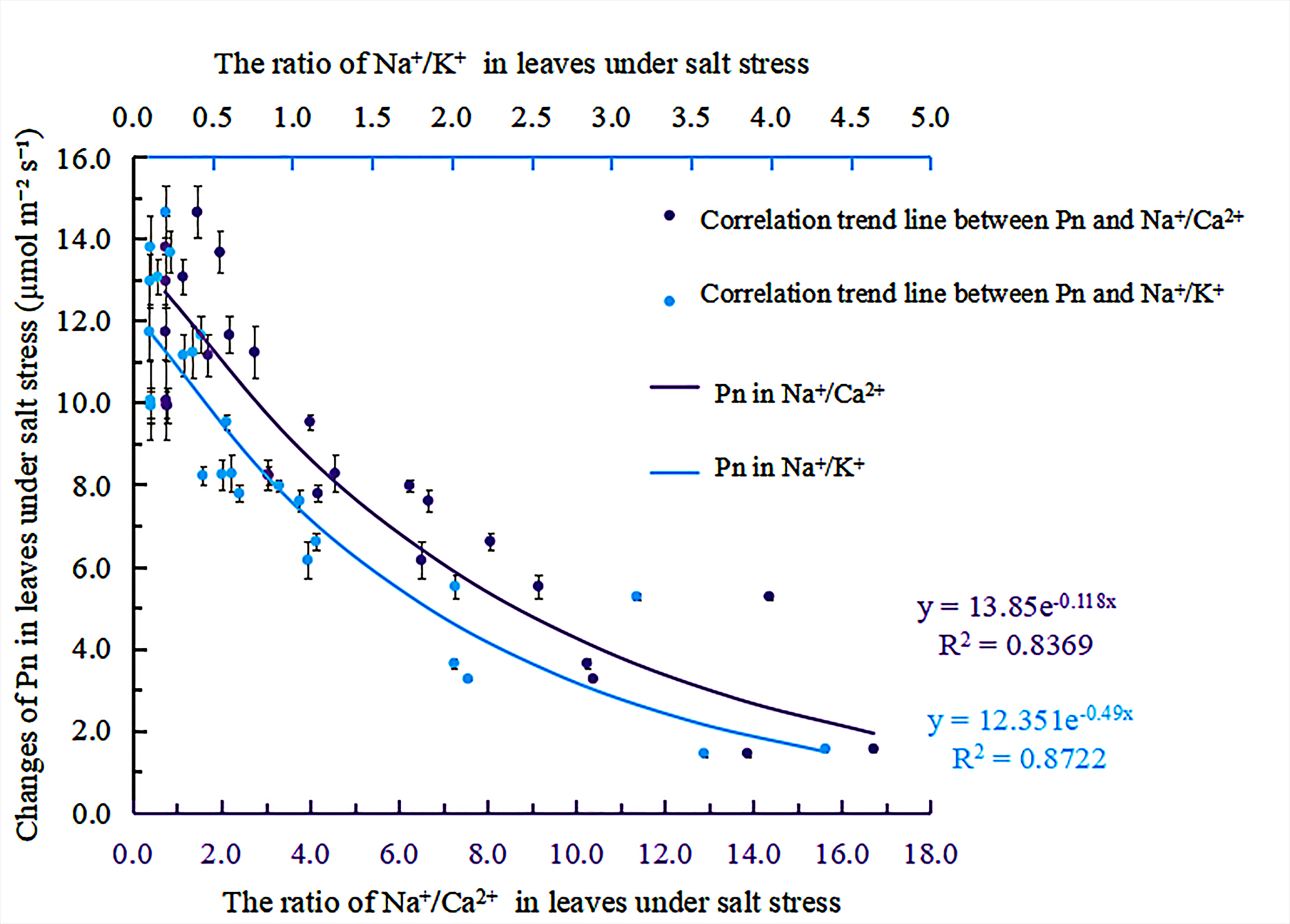


**Fig. S16** Correlation of Na^+^/ Ca^2+^ and Na^+^/ K^+^ in the Leaves of *Salix matsudana* to Pn under Salt Stress (data in Fig.4A)


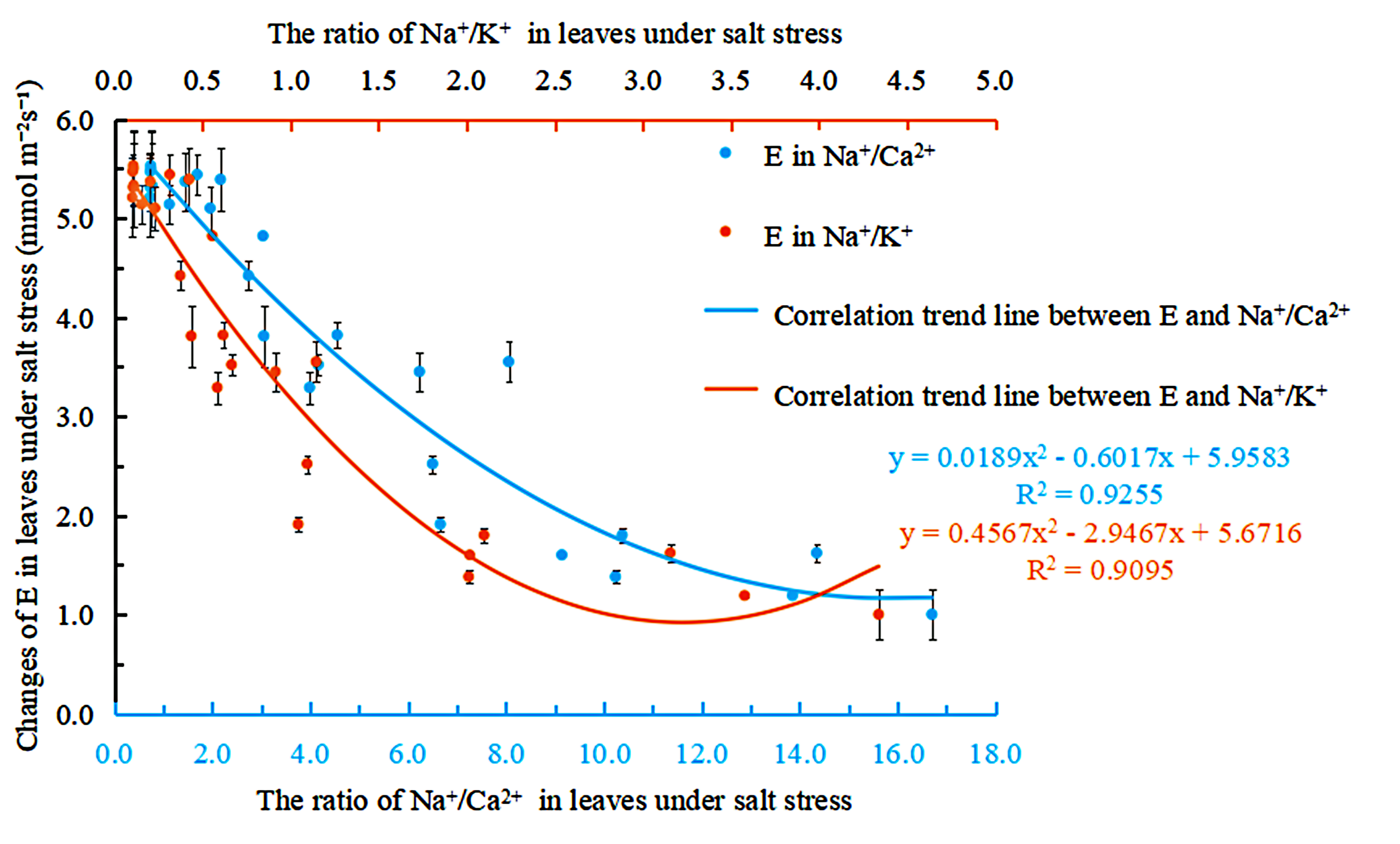


**Fig. S17** Correlation of Na^+^/ Ca^2+^ and Na^+^/ K^+^ in the Leaves of *Salix matsudana* to E under Salt Stress (data in Fig.4B)


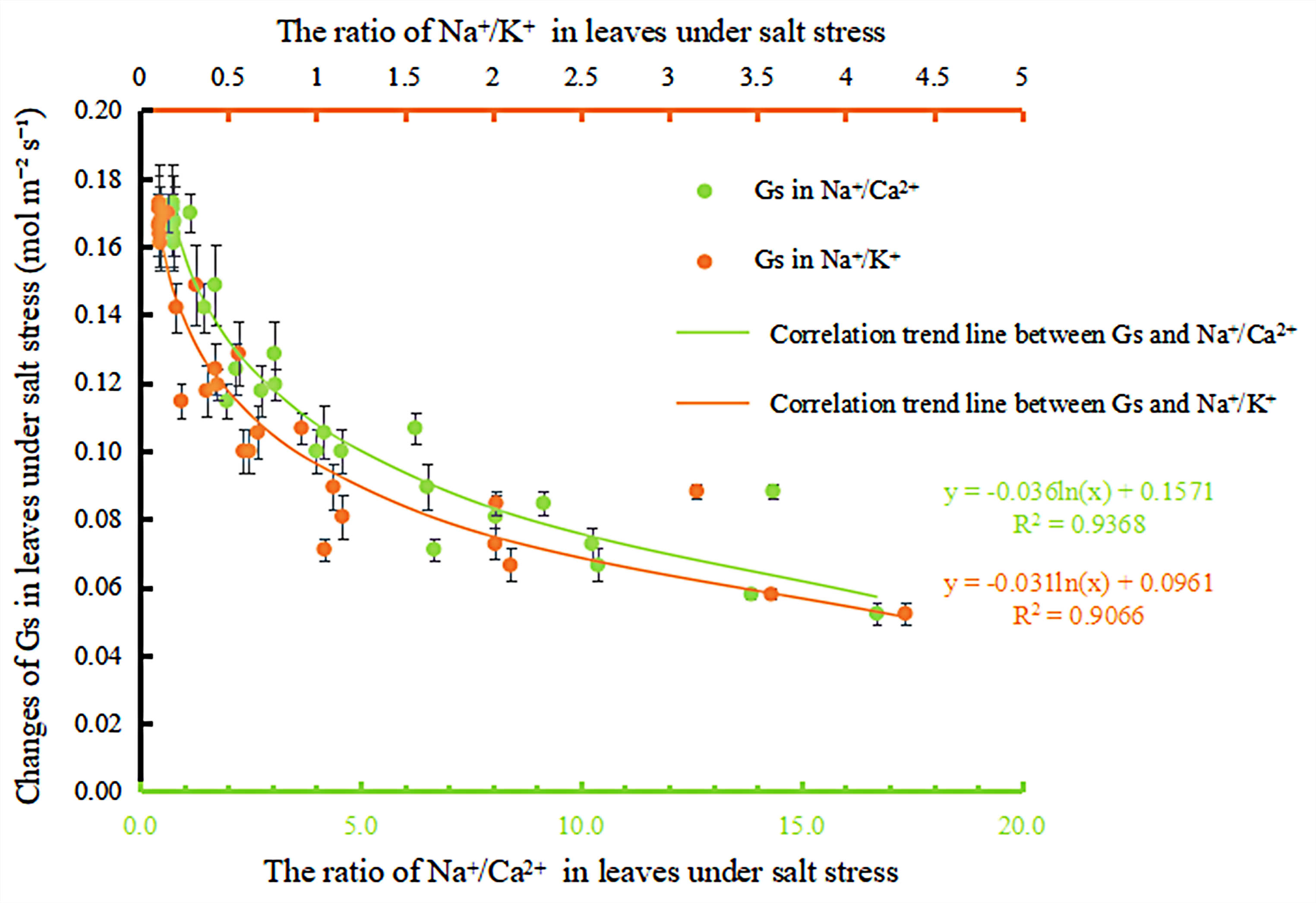


**Fig. S18** Analysis of the Correlation of Na^+^/Ca^2+^ and Na^+^/K^+^ in the Leaves of *Salix matsudana* with Gs under Salt Stress (data in Fig.4C)

**
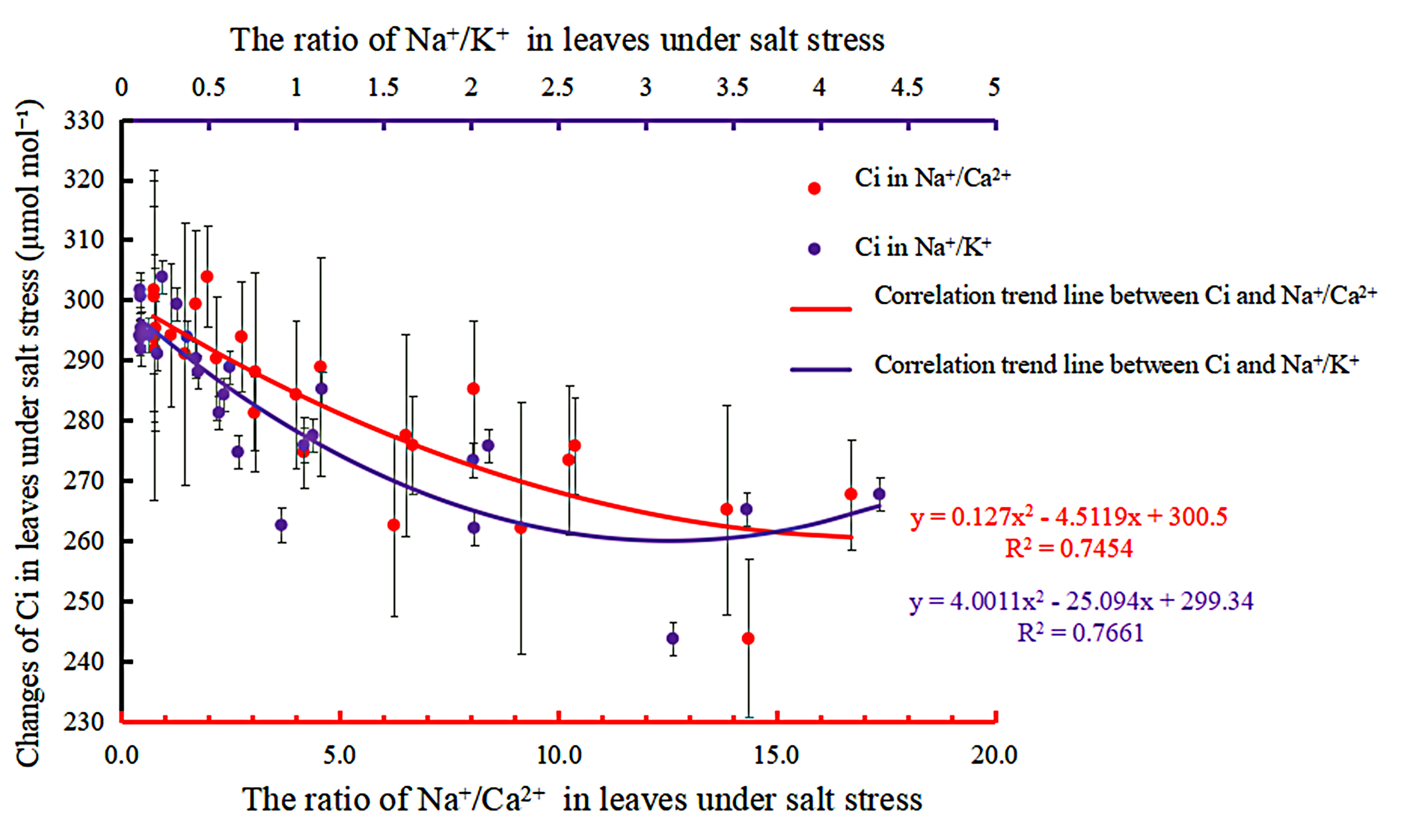
**

**Fig. S19** Correlation of Na^+^/Ca^2+^ and Na^+^/ K^+^ in the Leaves of *Salix matsudana* to Ci under Salt Stress (data in Fig.4D)


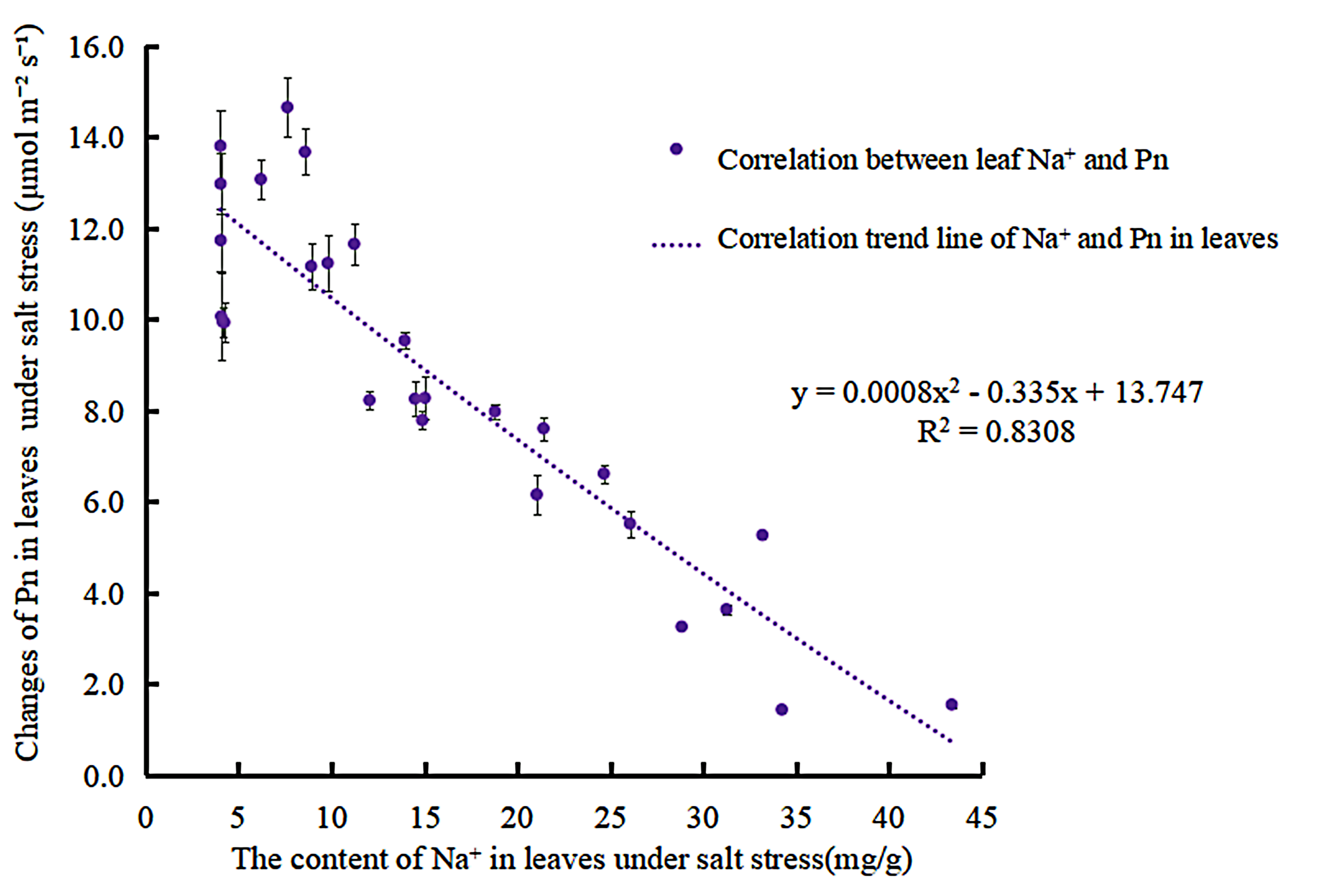


**Fig. S20** Correlation of Na^+^ and Pn in the Leaves of *Salix matsudana* under Salt Stress (data in Fig.4E)


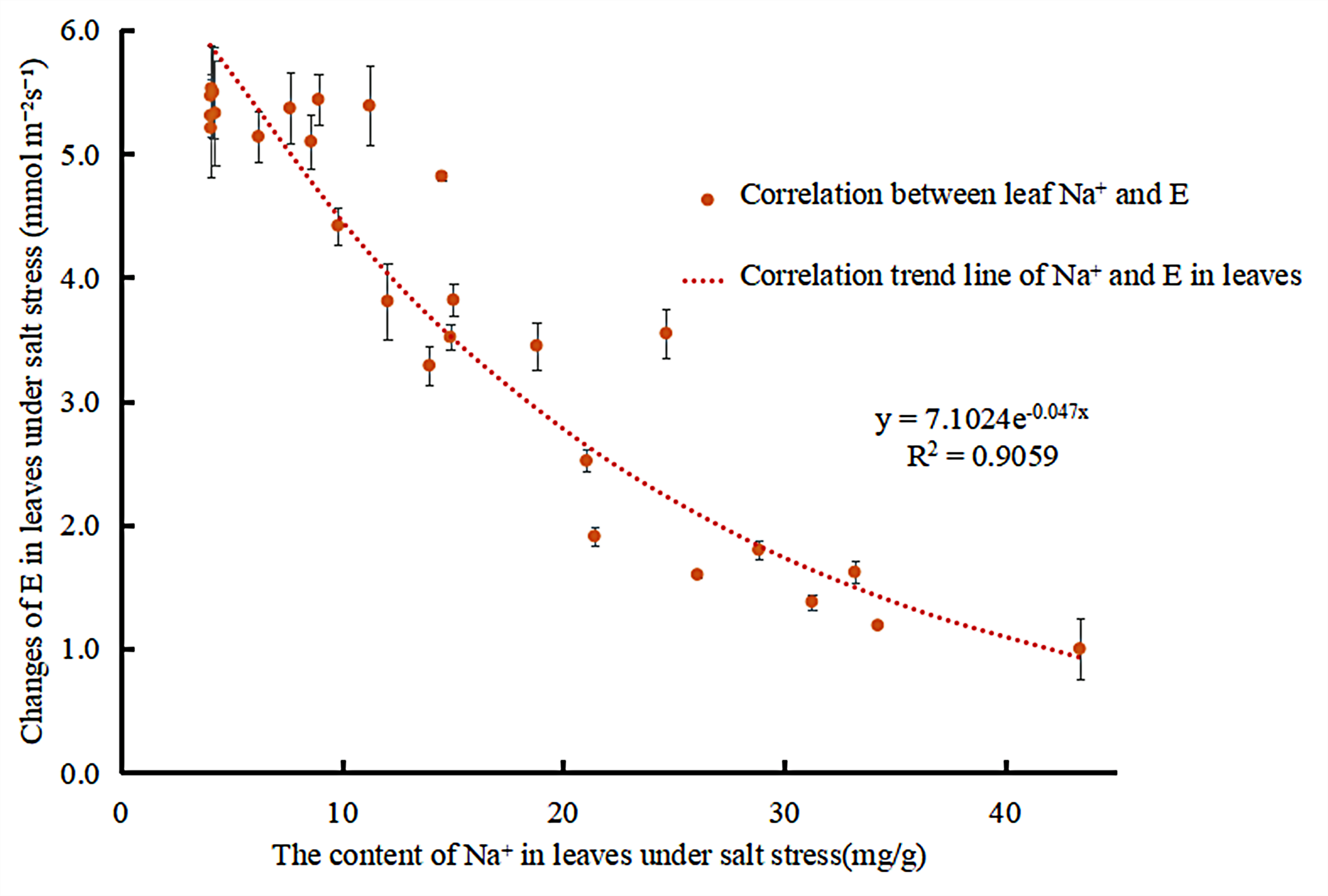


**Fig. S21** Correlation of Na^+^ in the Leaves of *Salix matsudana* to E under Salt Stress (data in Fig.4F)


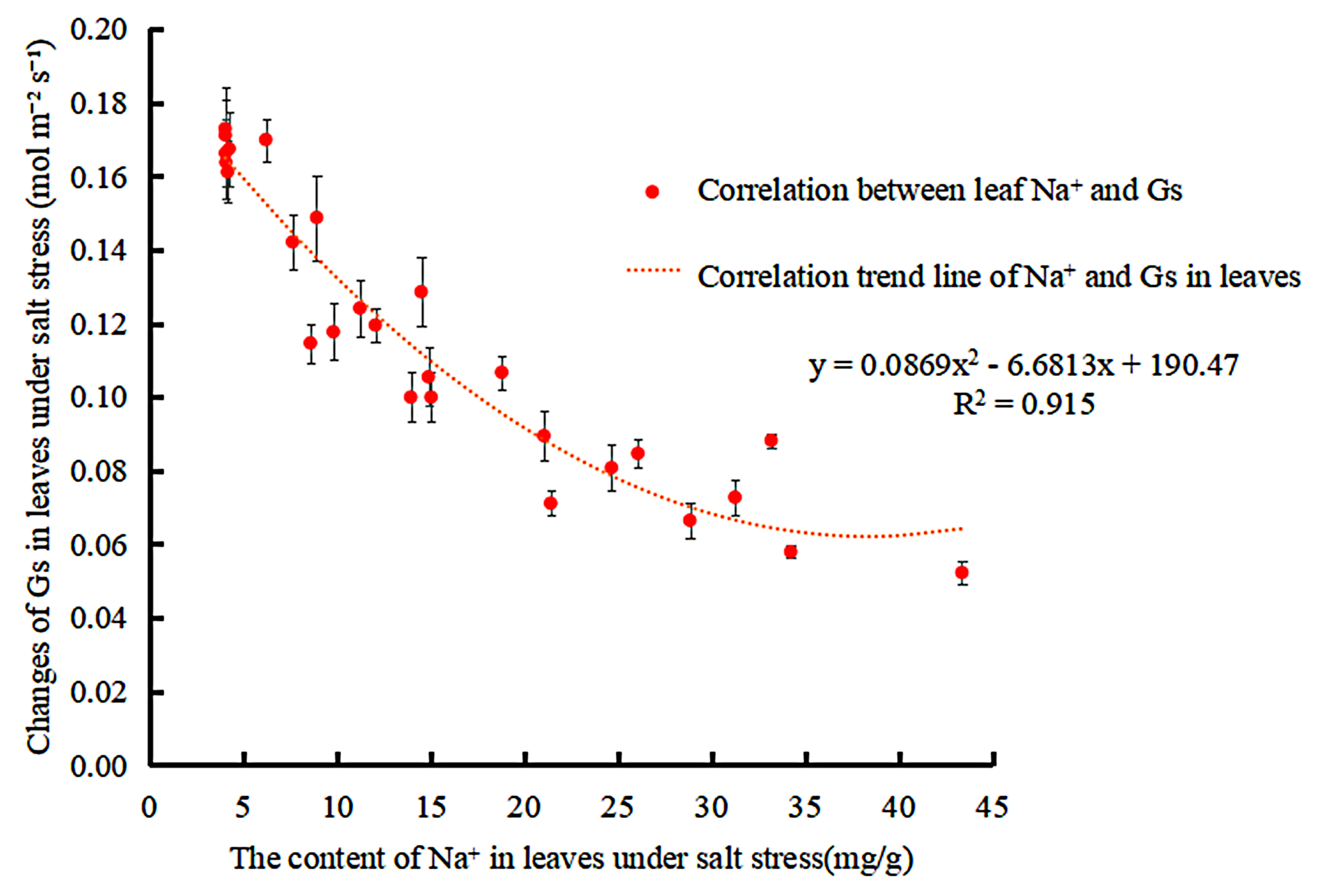


**Fig. S22** Correlation of Na^+^ in the Leaves of *Salix matsudana* with Gs under Salt Stress (data in Fig.4G)


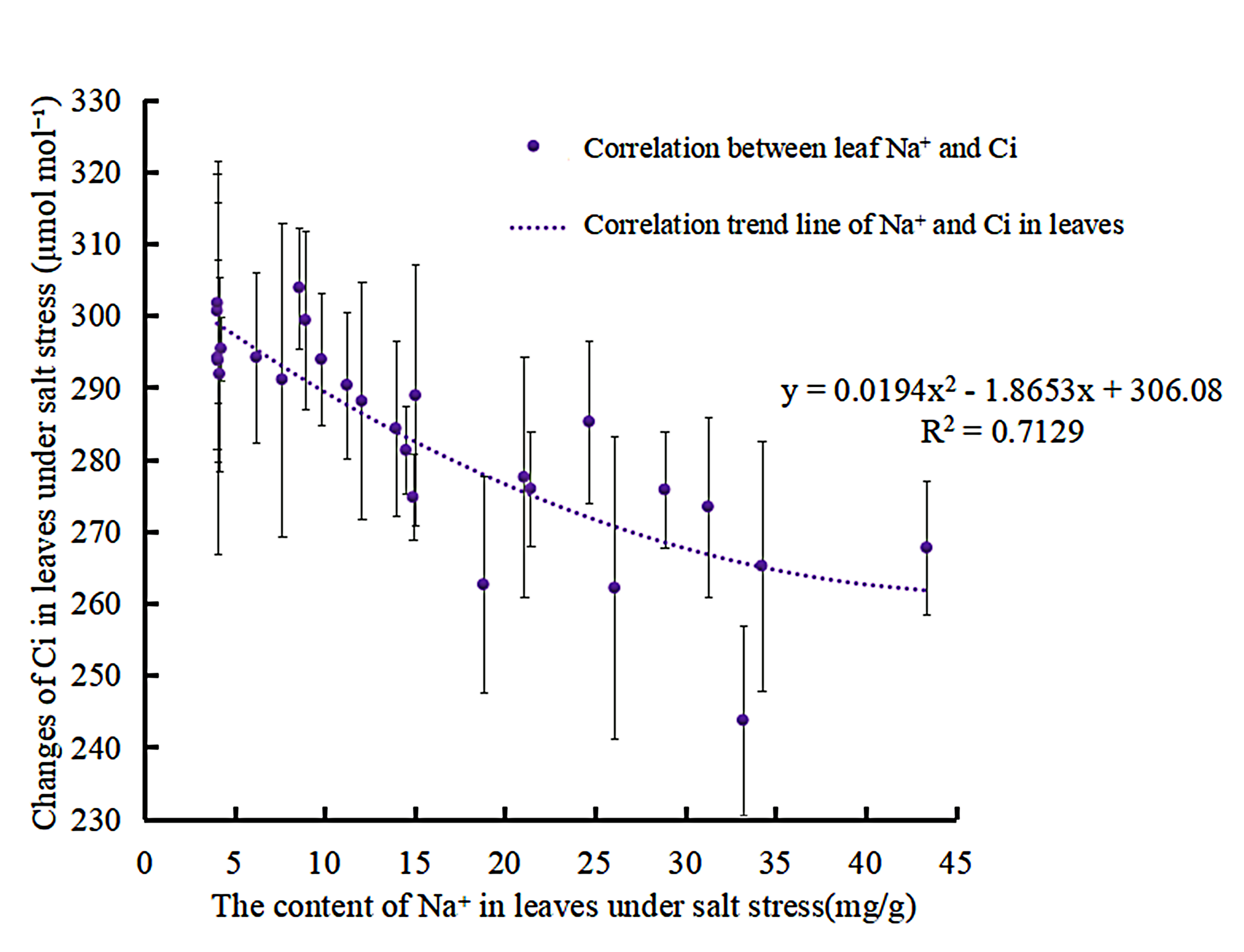


**Fig. S23** Correlation of Na^+^ in the Leaves of *Salix matsudana* with Gs under Salt Stress (data in Fig.4H)
