# Supplementary figures and images for: Study on the Relationship of Ions (Na, K, Ca) Absorption and Distribution to Photosynthetic Response of Salix matsudana Koidz Under Salt Stress
Source: Front Plant Sci. 2022 May 3;13:860111. doi: 10.3389/fpls.2022.860111 (PMC9111522; doi:10.3389/fpls.2022.860111)

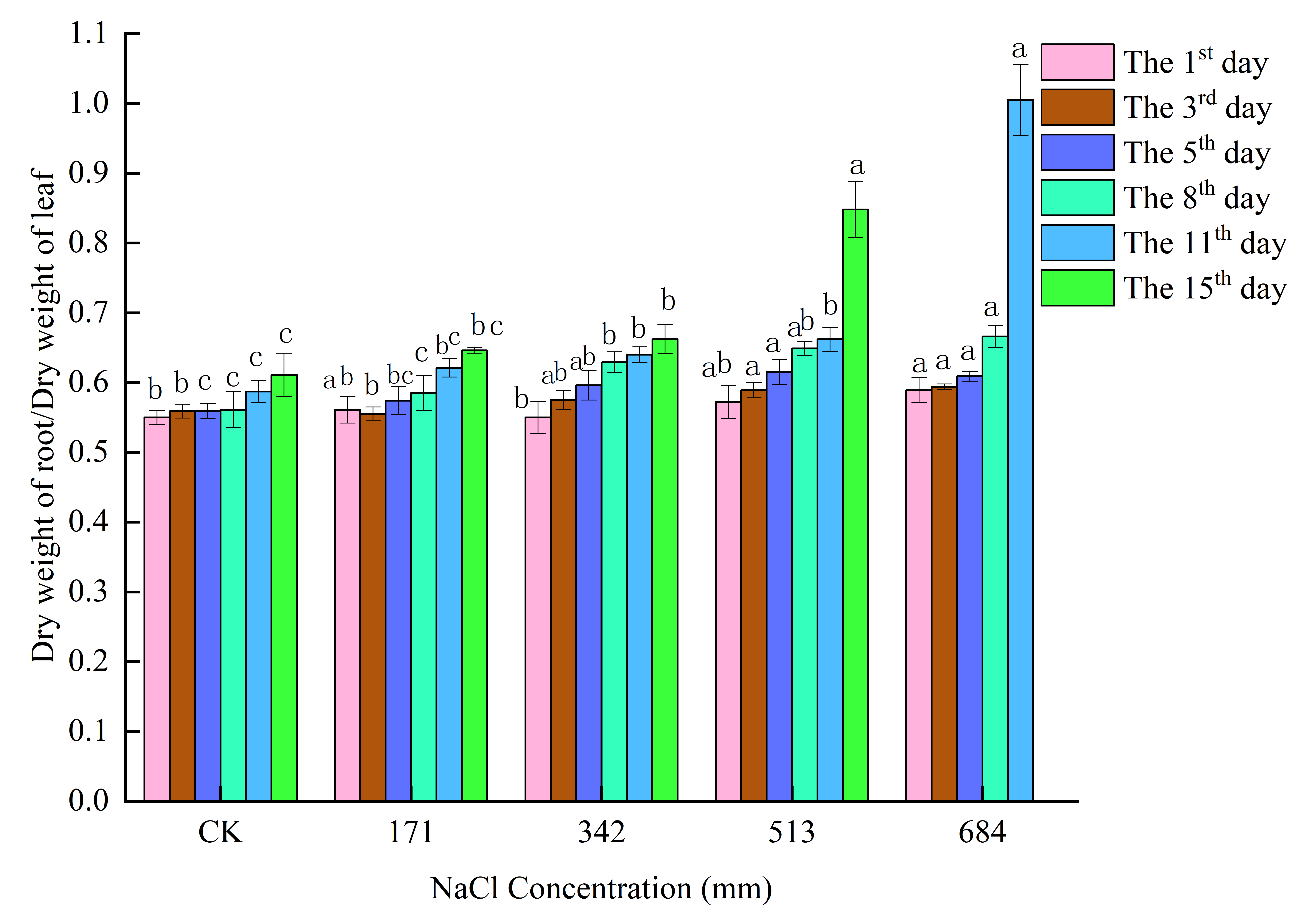

Supplement: Supplementary file 2 [file Data_Sheet_3.ZIP › Fig1a.tif]

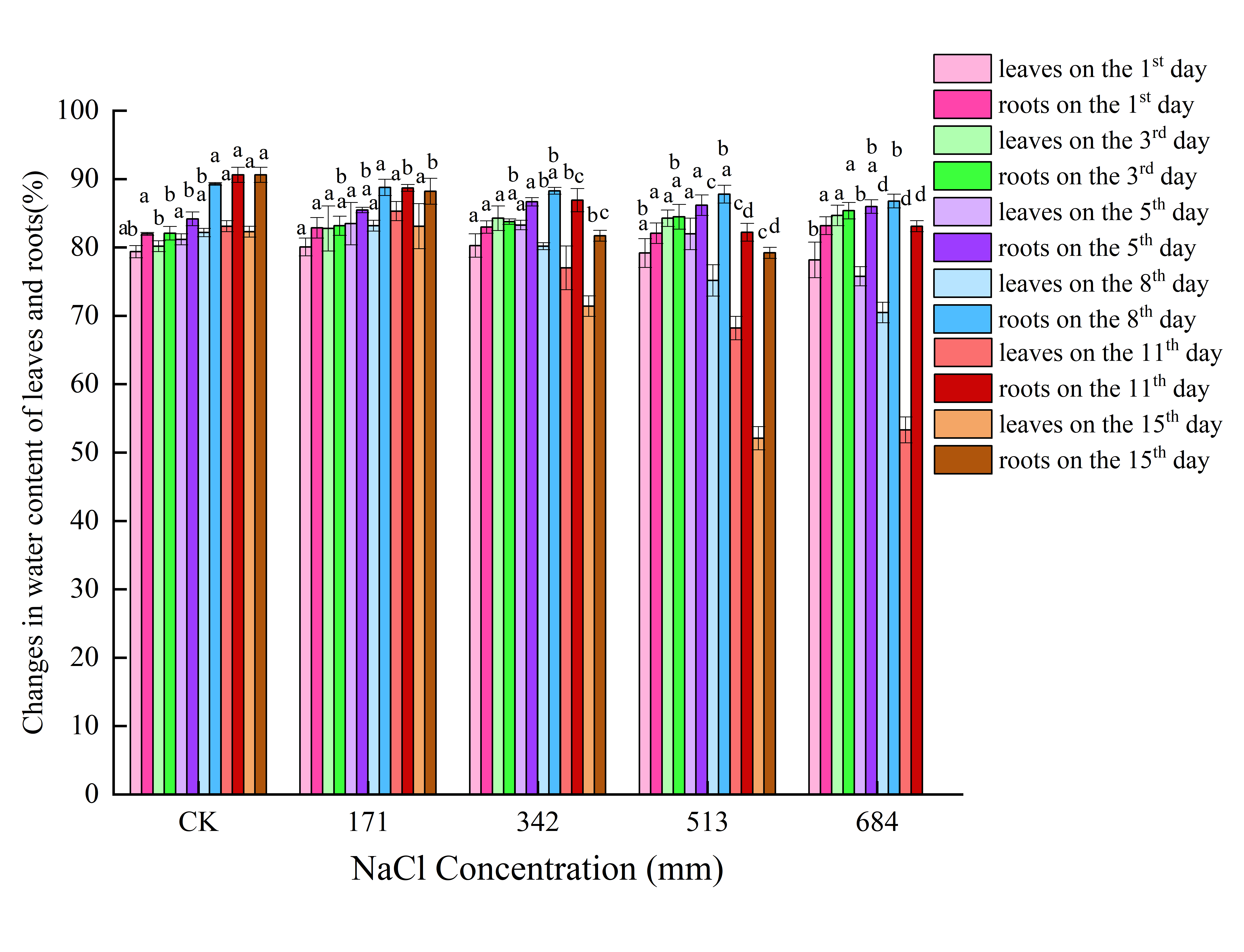

Supplement: Supplementary file 2 [file Data_Sheet_3.ZIP › Fig1b.tif]

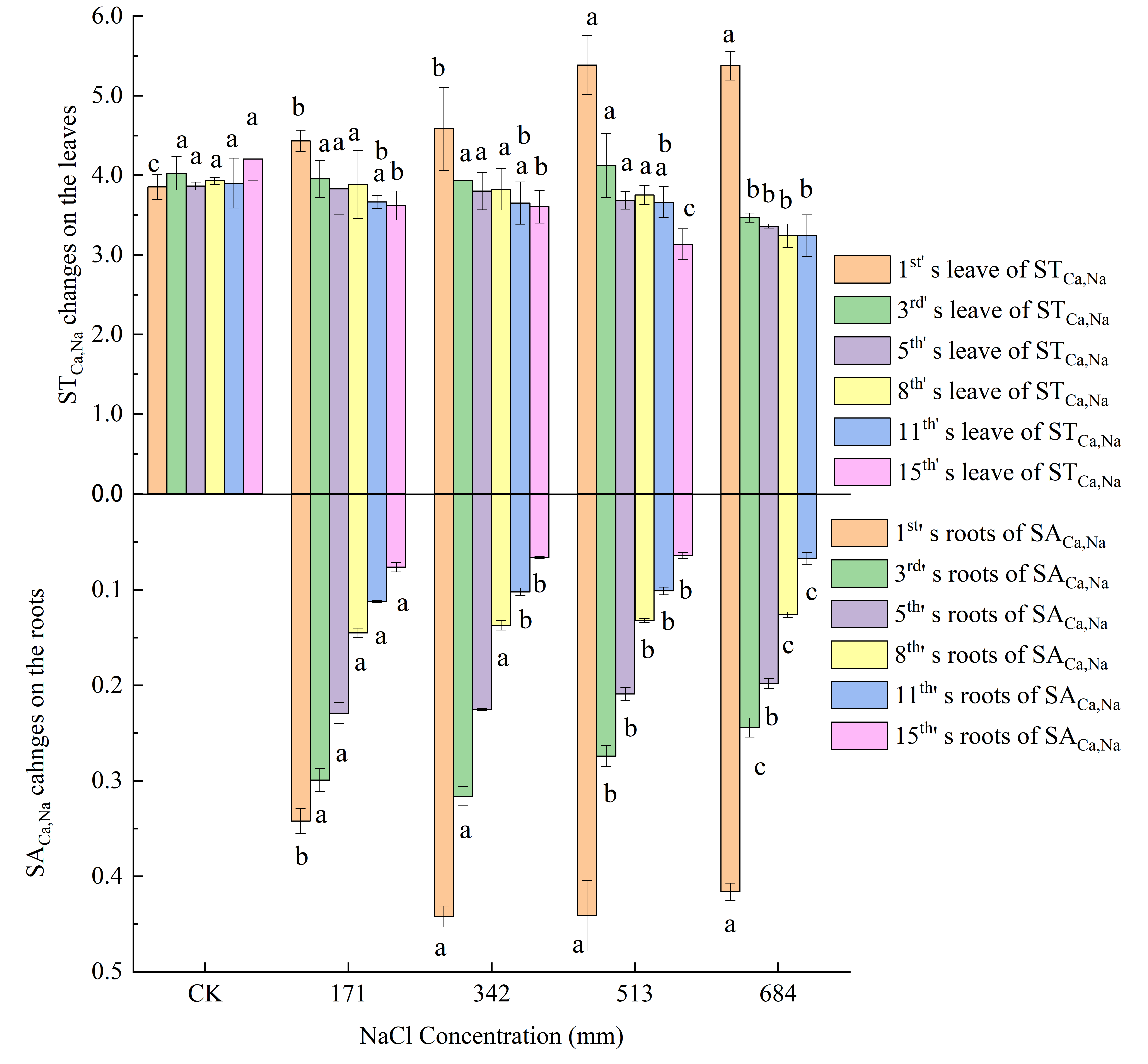

Supplement: Supplementary file 2 [file Data_Sheet_3.ZIP › Fig1c.tif]

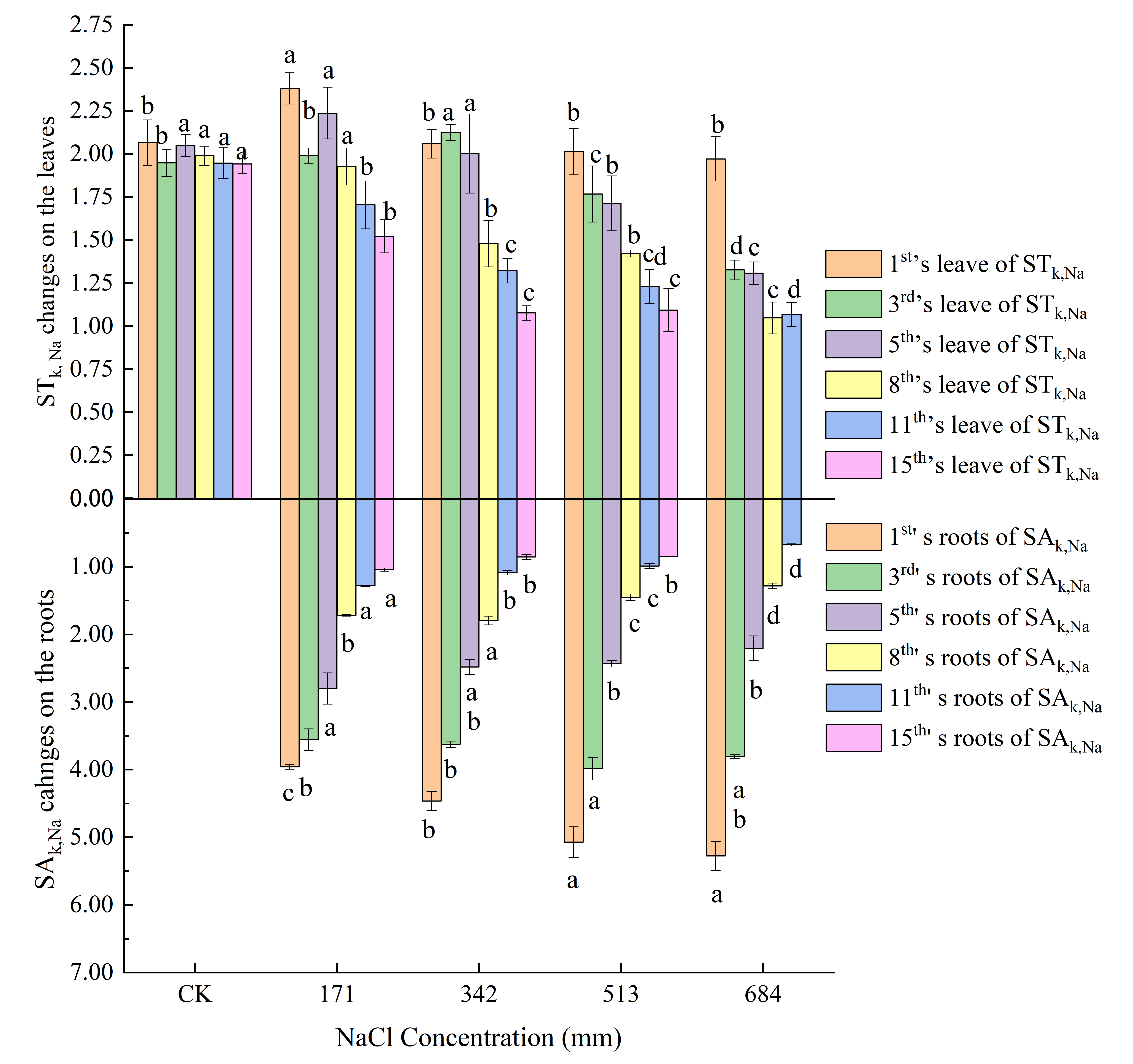

Supplement: Supplementary file 2 [file Data_Sheet_3.ZIP › Fig1d.tif]

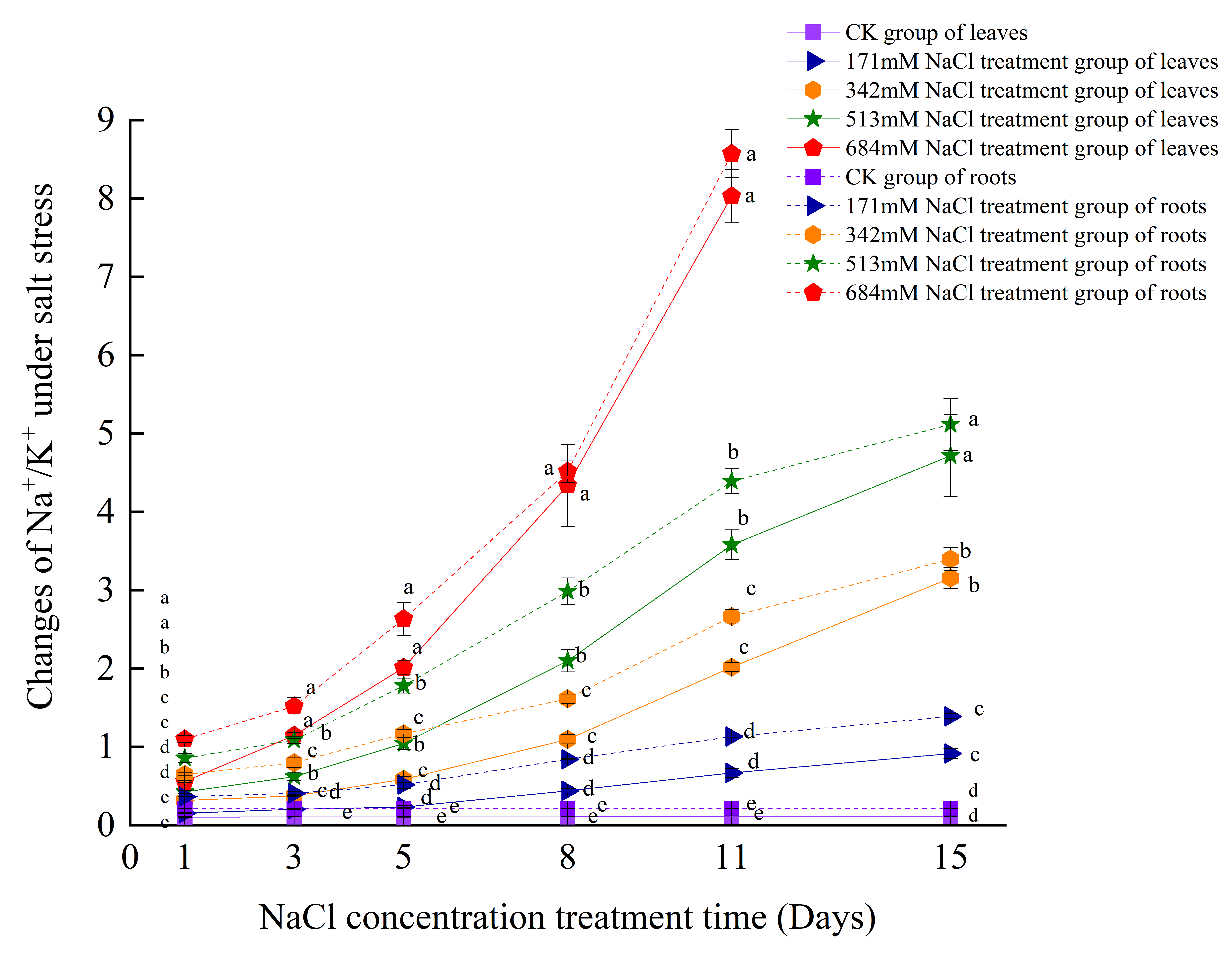

Supplement: Supplementary file 2 [file Data_Sheet_3.ZIP › Fig2a.tif]

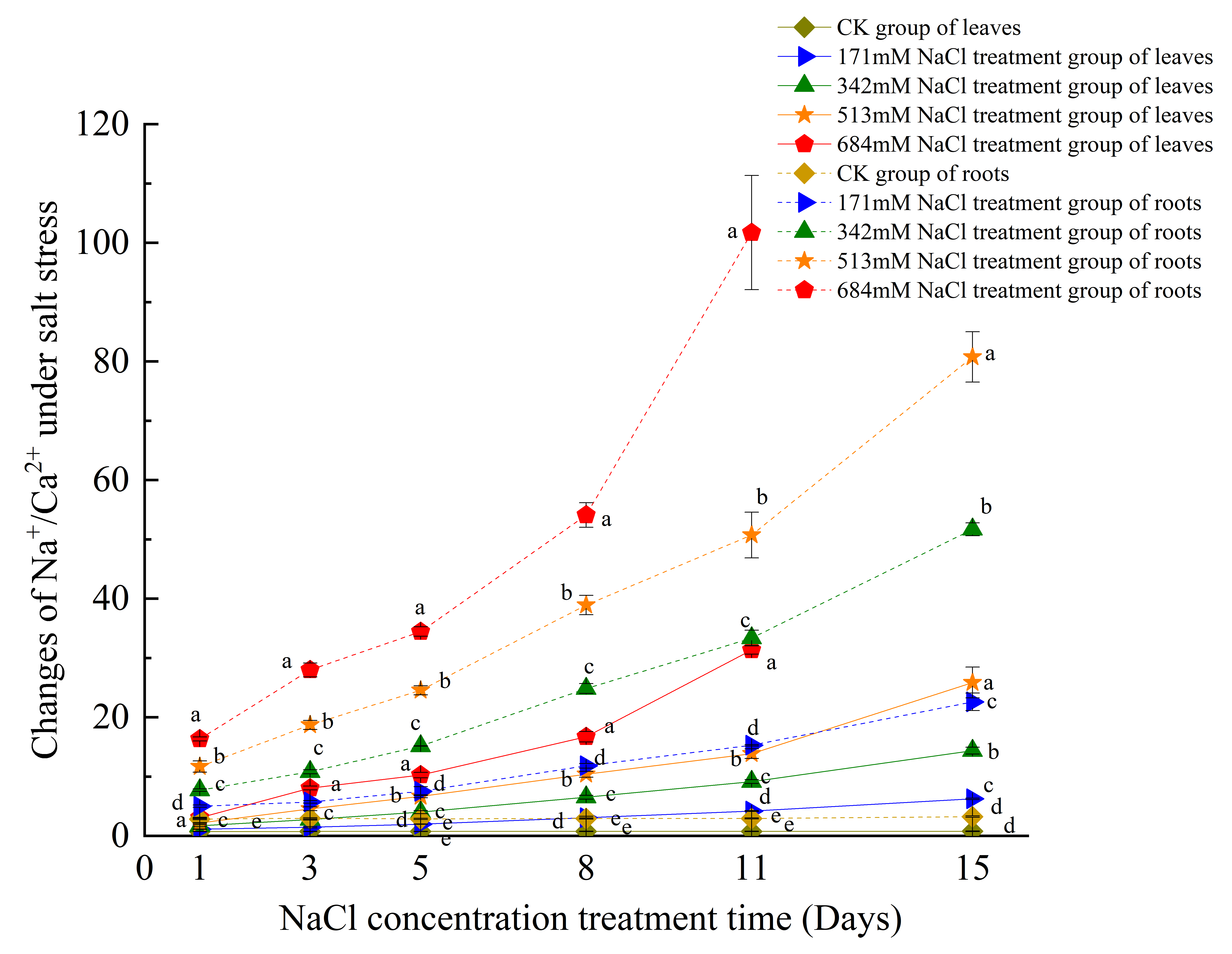

Supplement: Supplementary file 2 [file Data_Sheet_3.ZIP › Fig2b.tif]

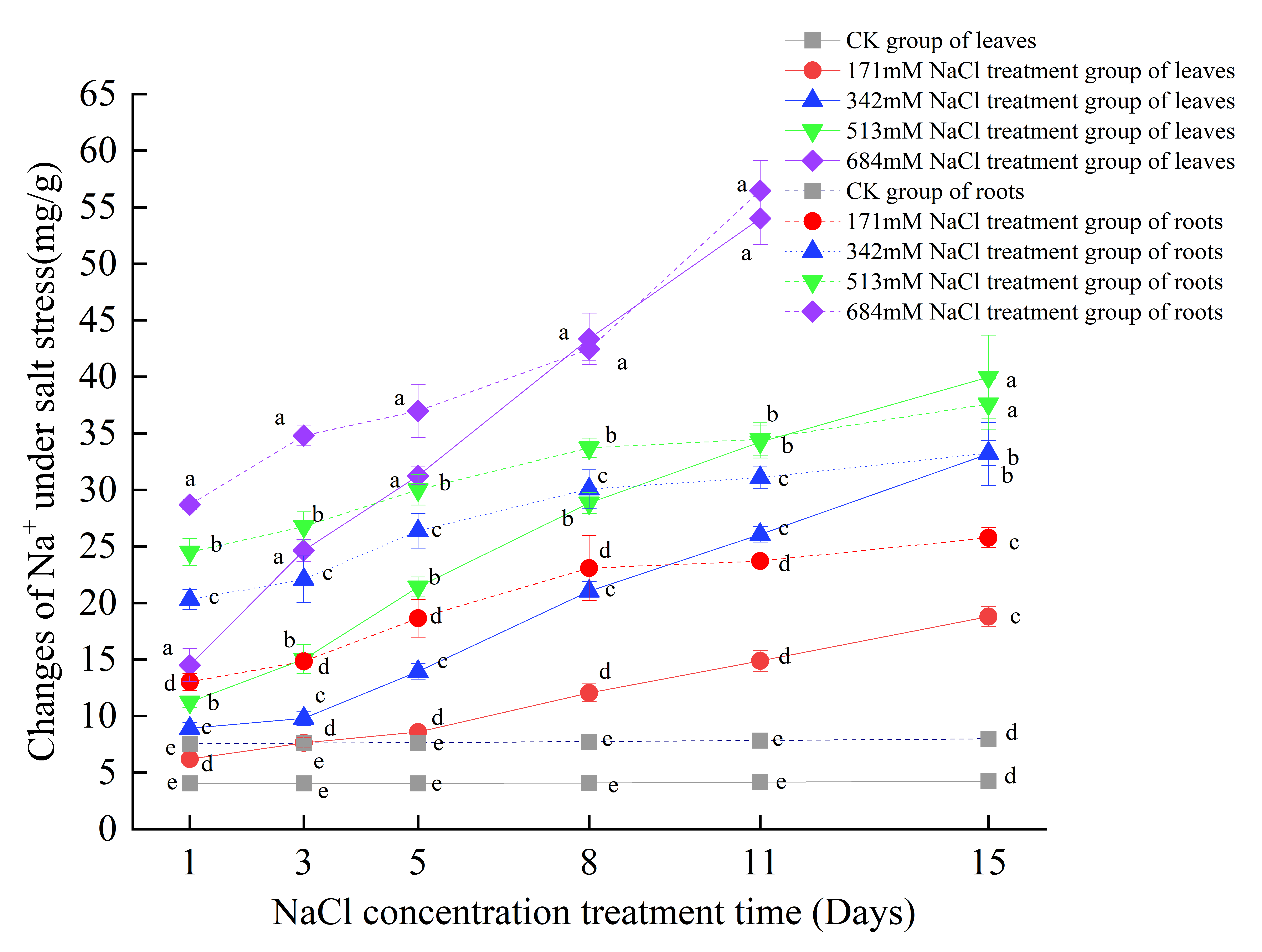

Supplement: Supplementary file 2 [file Data_Sheet_3.ZIP › Fig2c.tif]

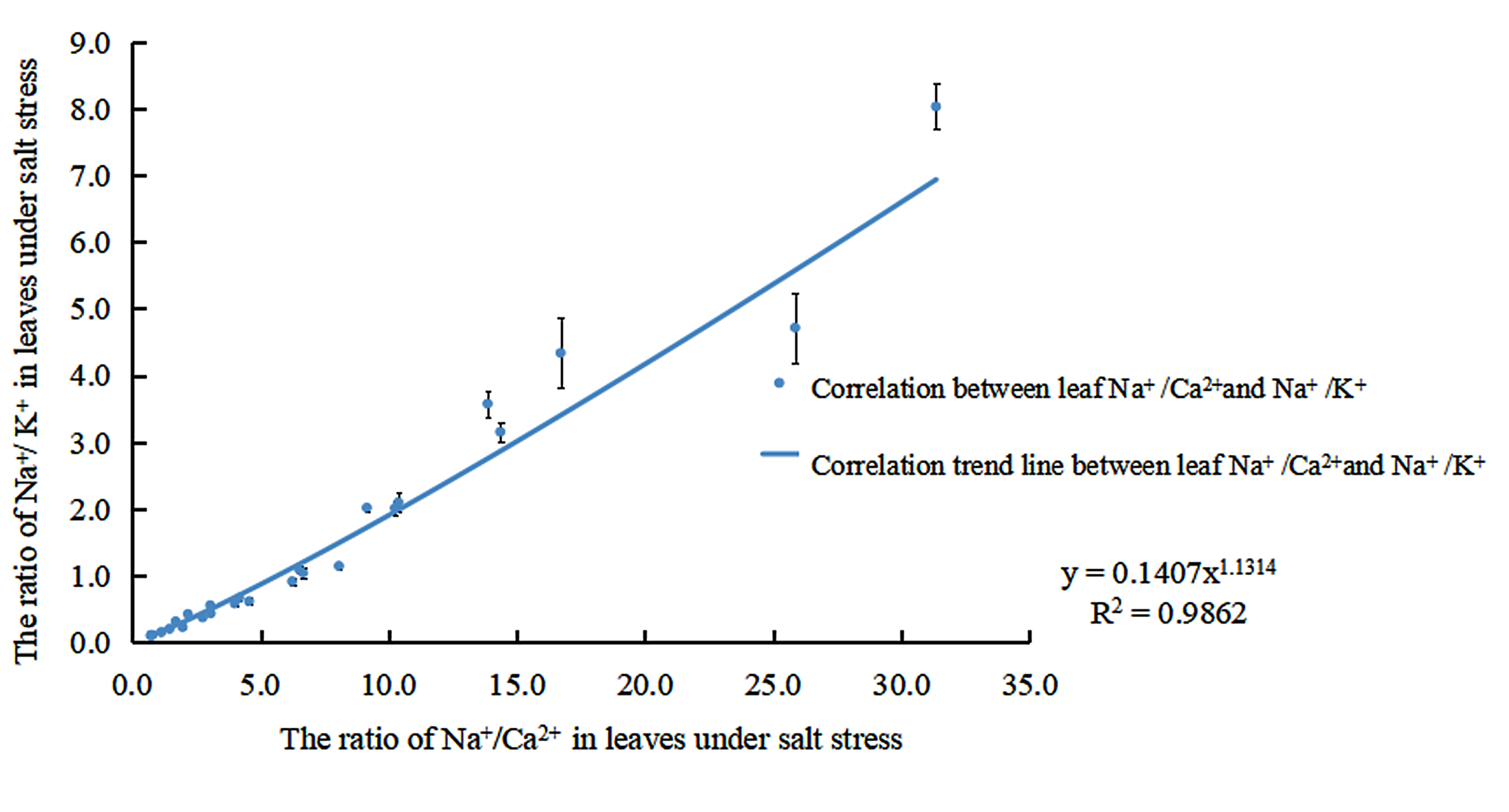

Supplement: Supplementary file 2 [file Data_Sheet_3.ZIP › Fig2d.tif]

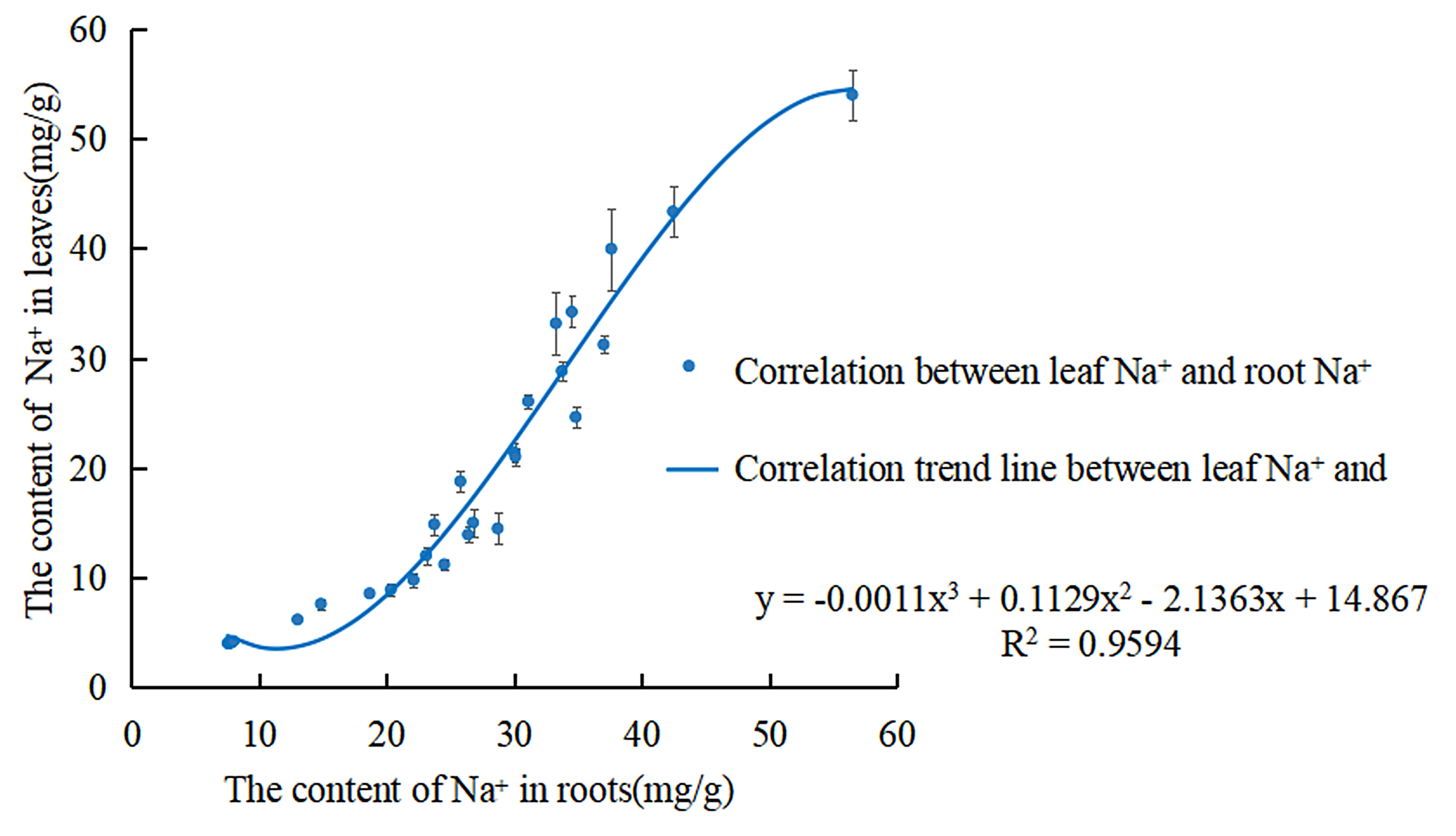

Supplement: Supplementary file 2 [file Data_Sheet_3.ZIP › Fig2e.tif]

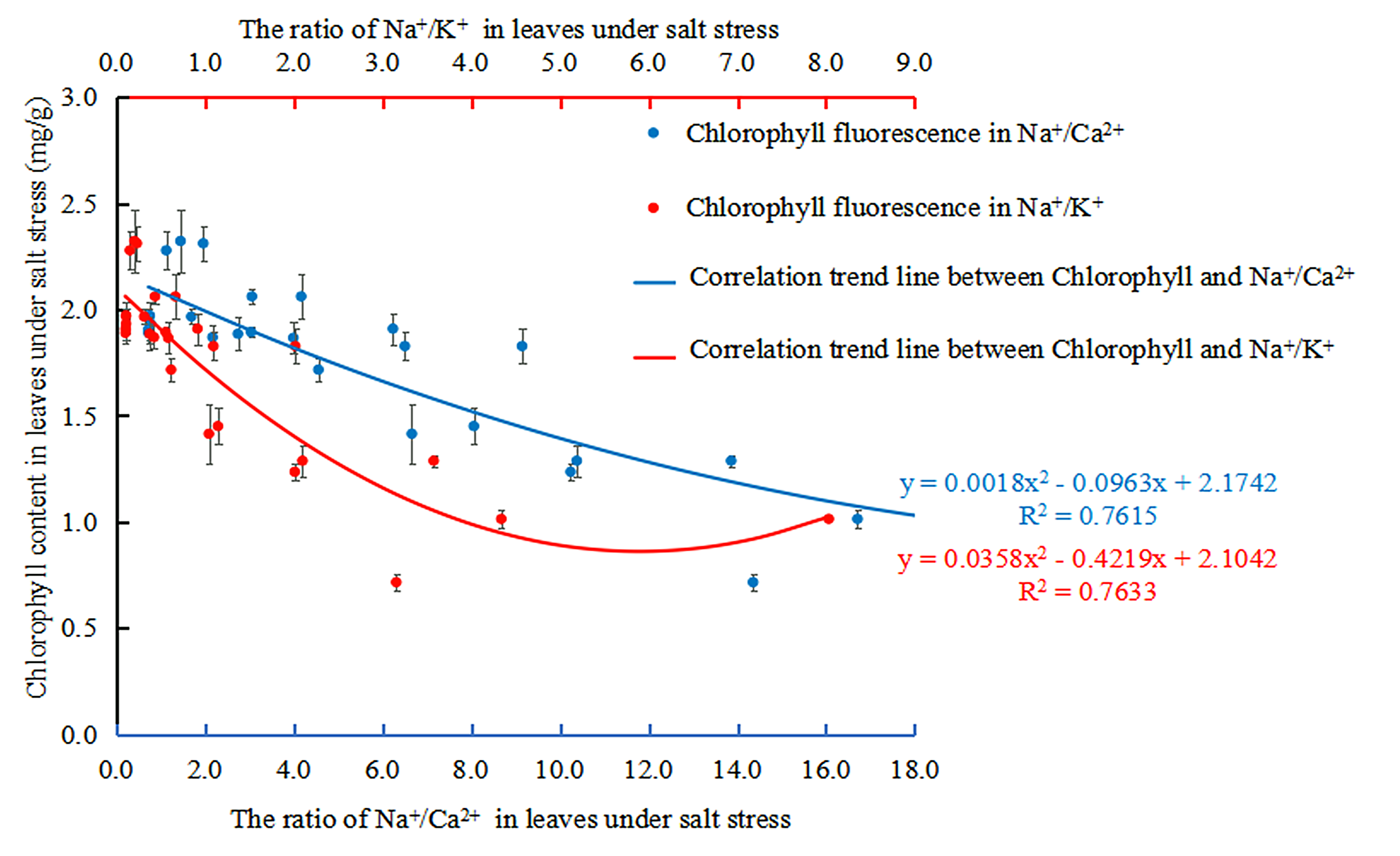

Supplement: Supplementary file 3 [file Data_Sheet_4.ZIP › Fig3a.tif]

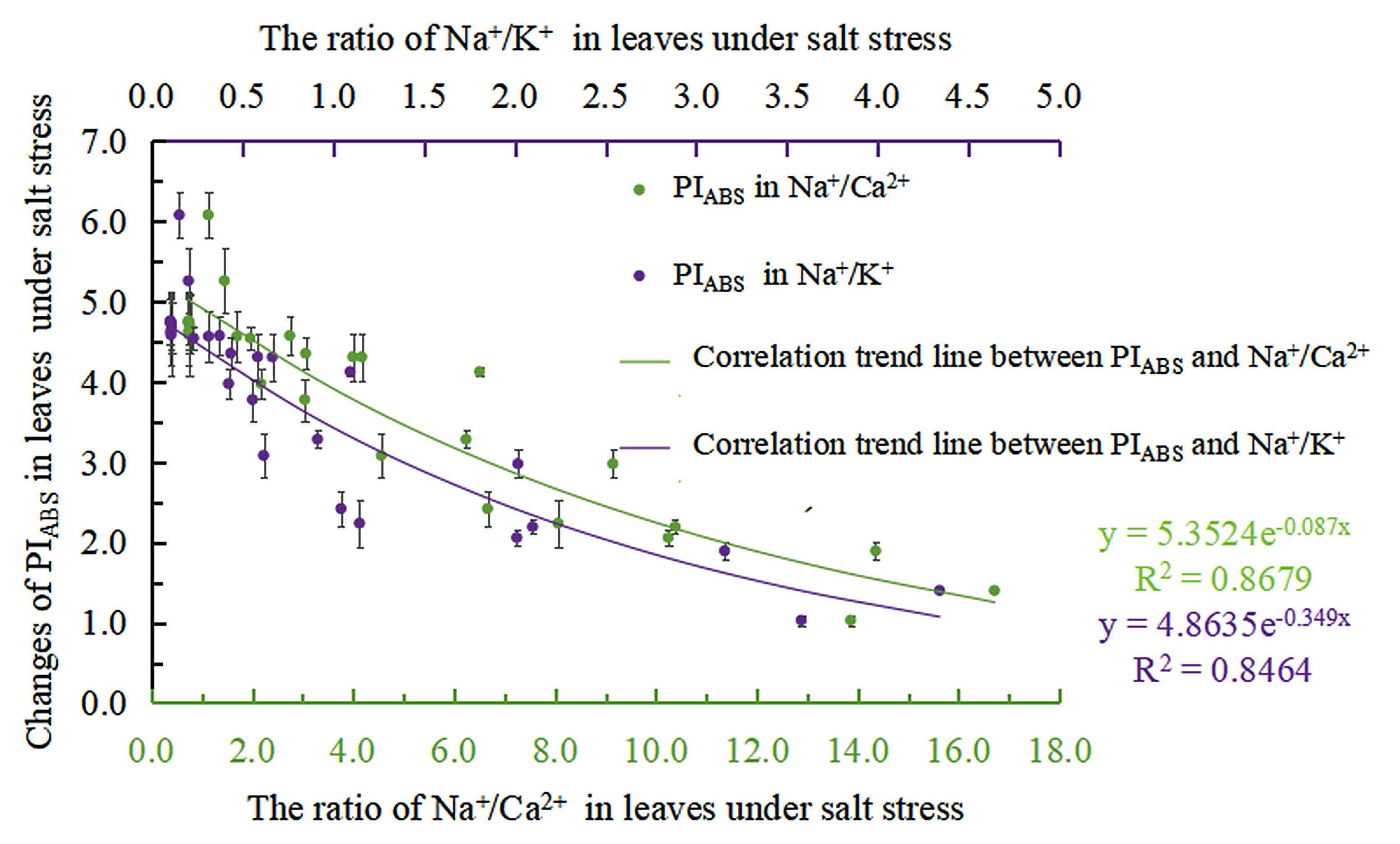

Supplement: Supplementary file 3 [file Data_Sheet_4.ZIP › Fig3b.tif]

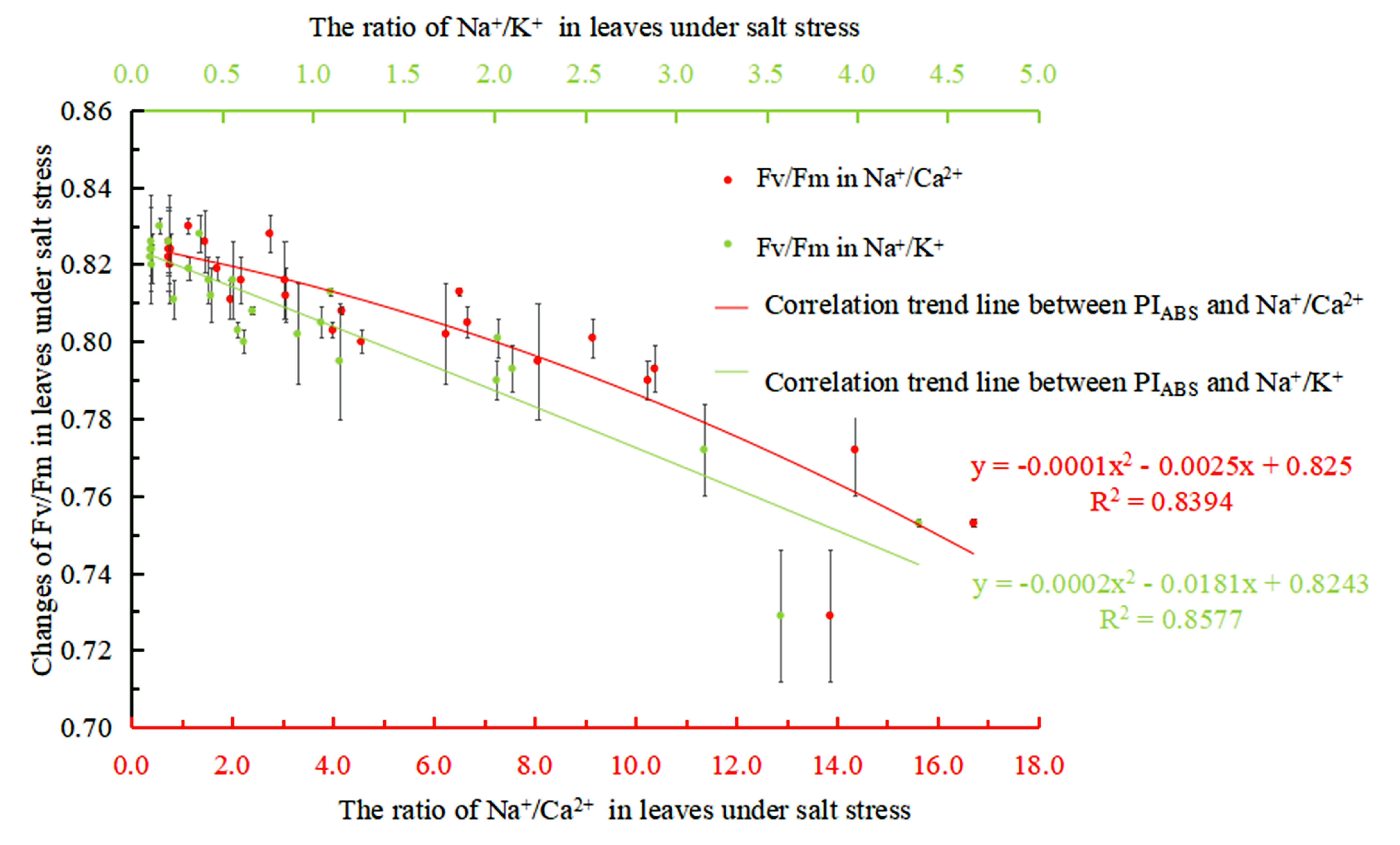

Supplement: Supplementary file 3 [file Data_Sheet_4.ZIP › Fig3c.tif]

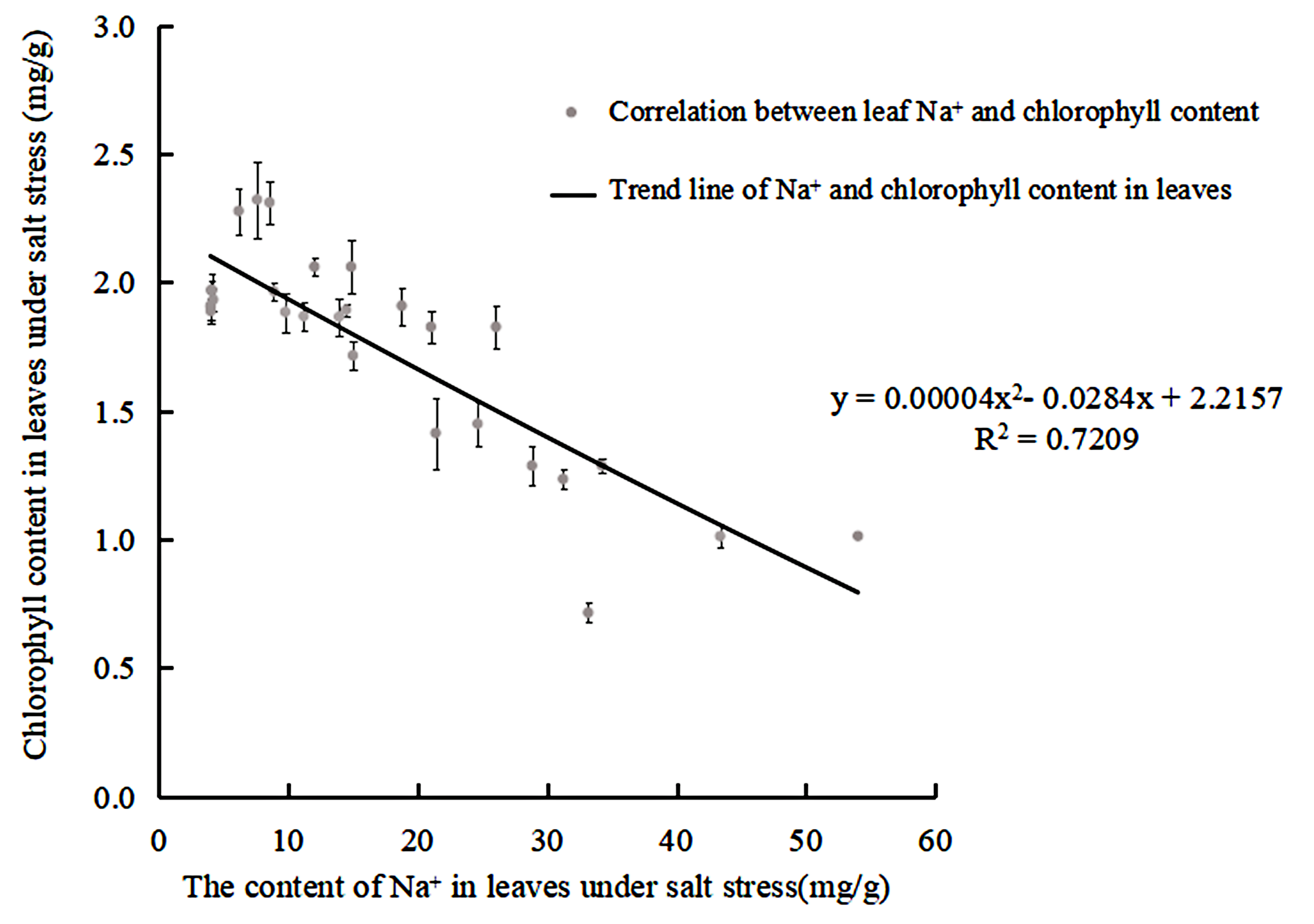

Supplement: Supplementary file 3 [file Data_Sheet_4.ZIP › Fig3d.tif]

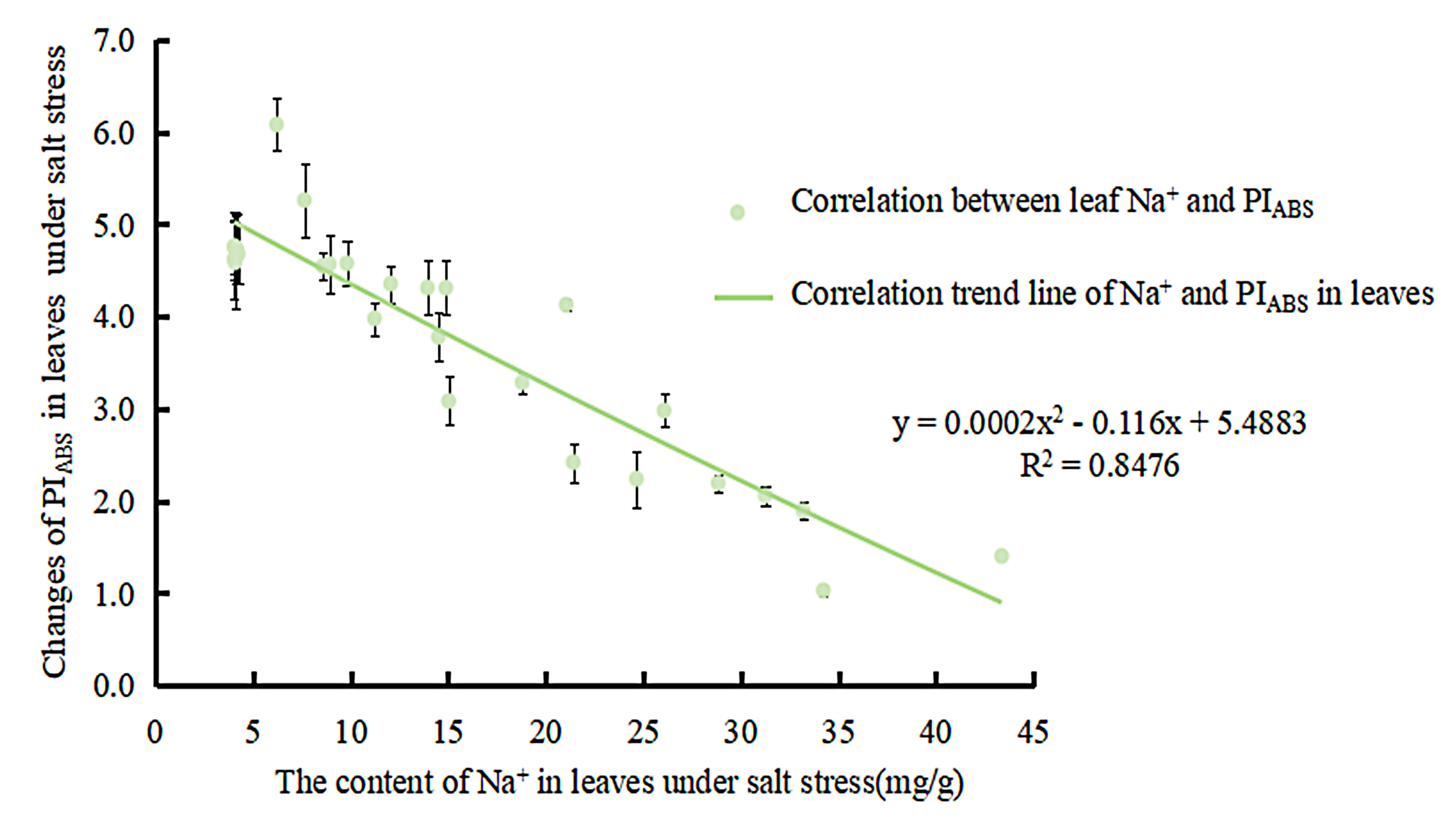

Supplement: Supplementary file 3 [file Data_Sheet_4.ZIP › Fig3e.tif]

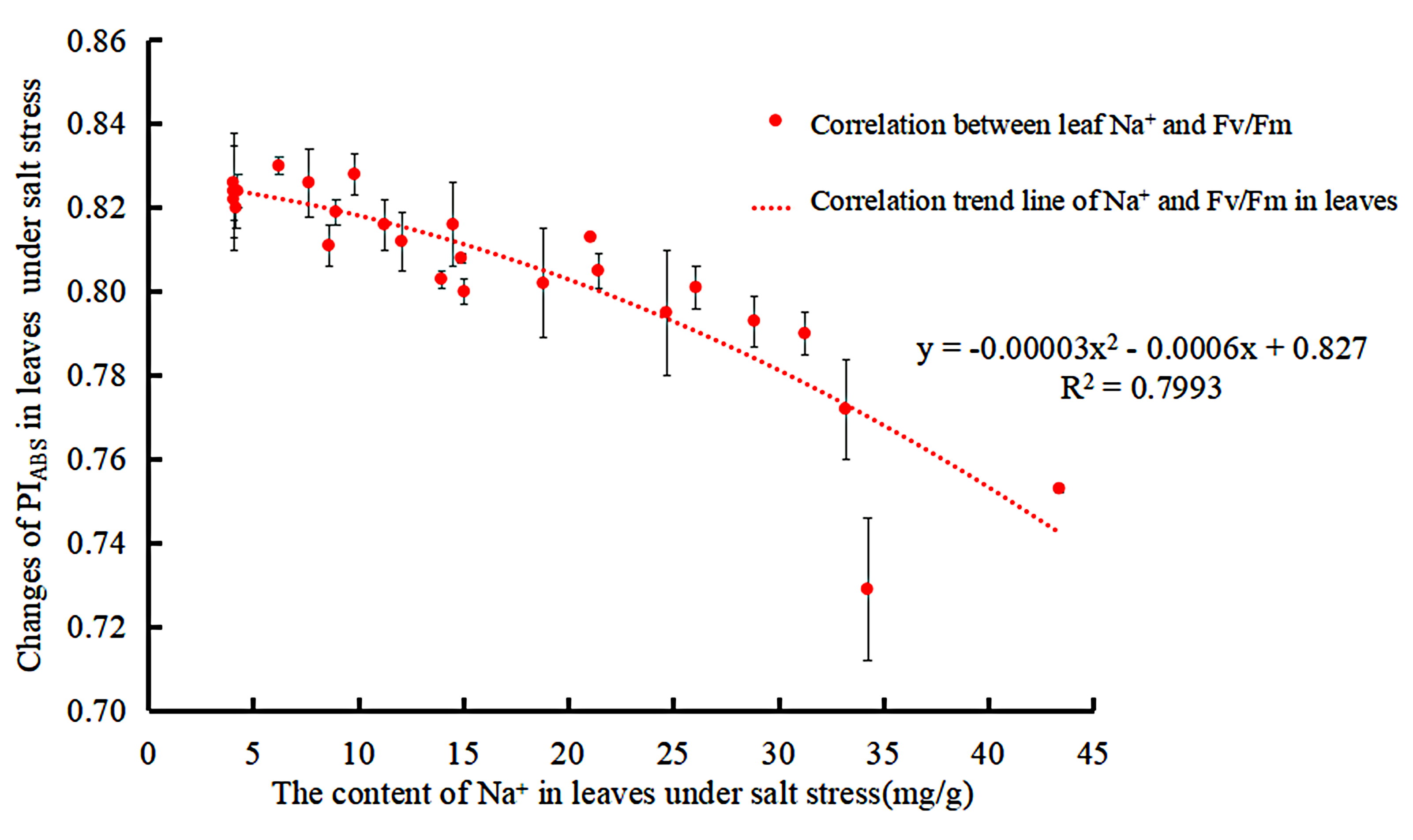

Supplement: Supplementary file 3 [file Data_Sheet_4.ZIP › Fig3f.tif]

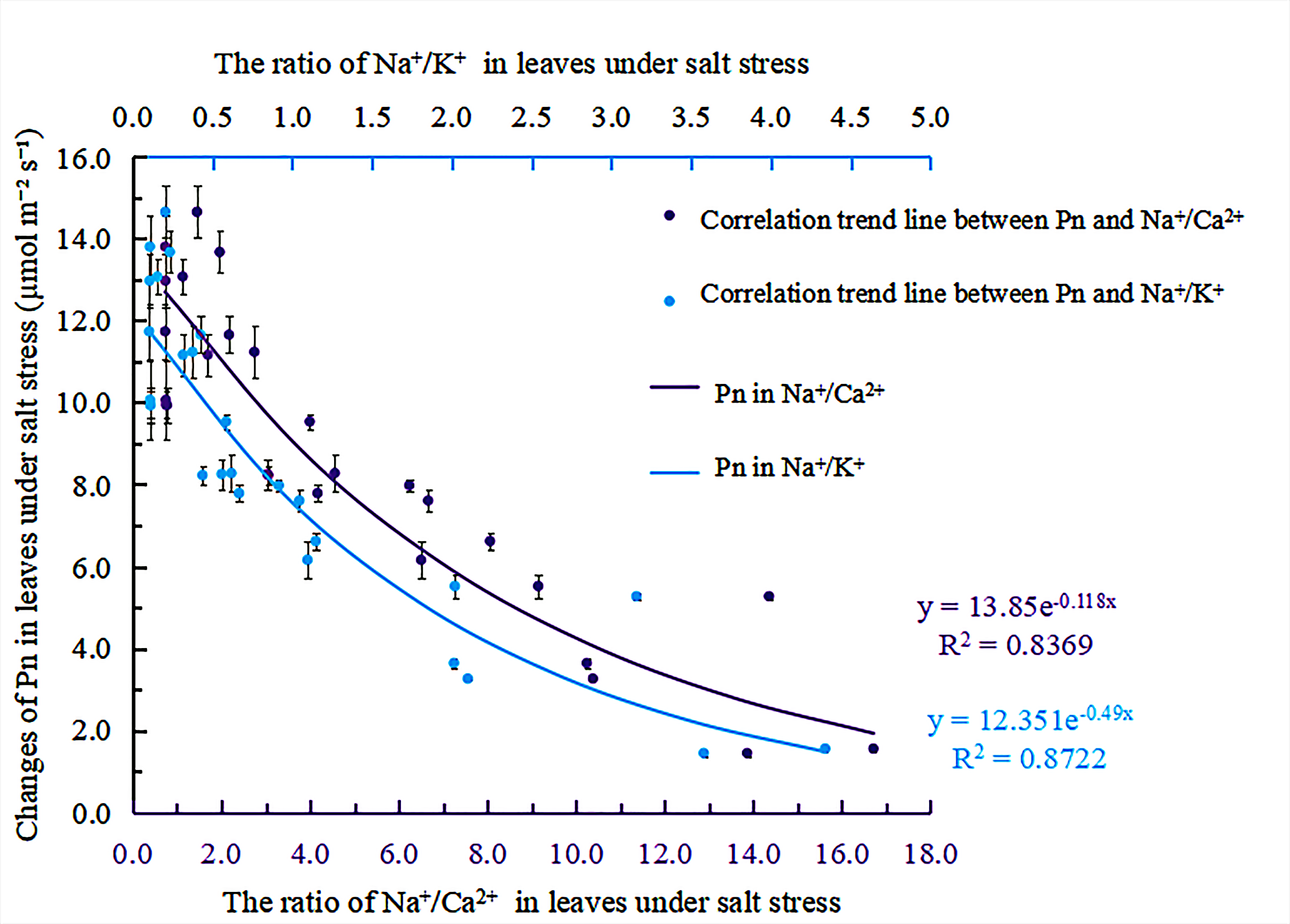

Supplement: Supplementary file 3 [file Data_Sheet_4.ZIP › Fig4a.tif]

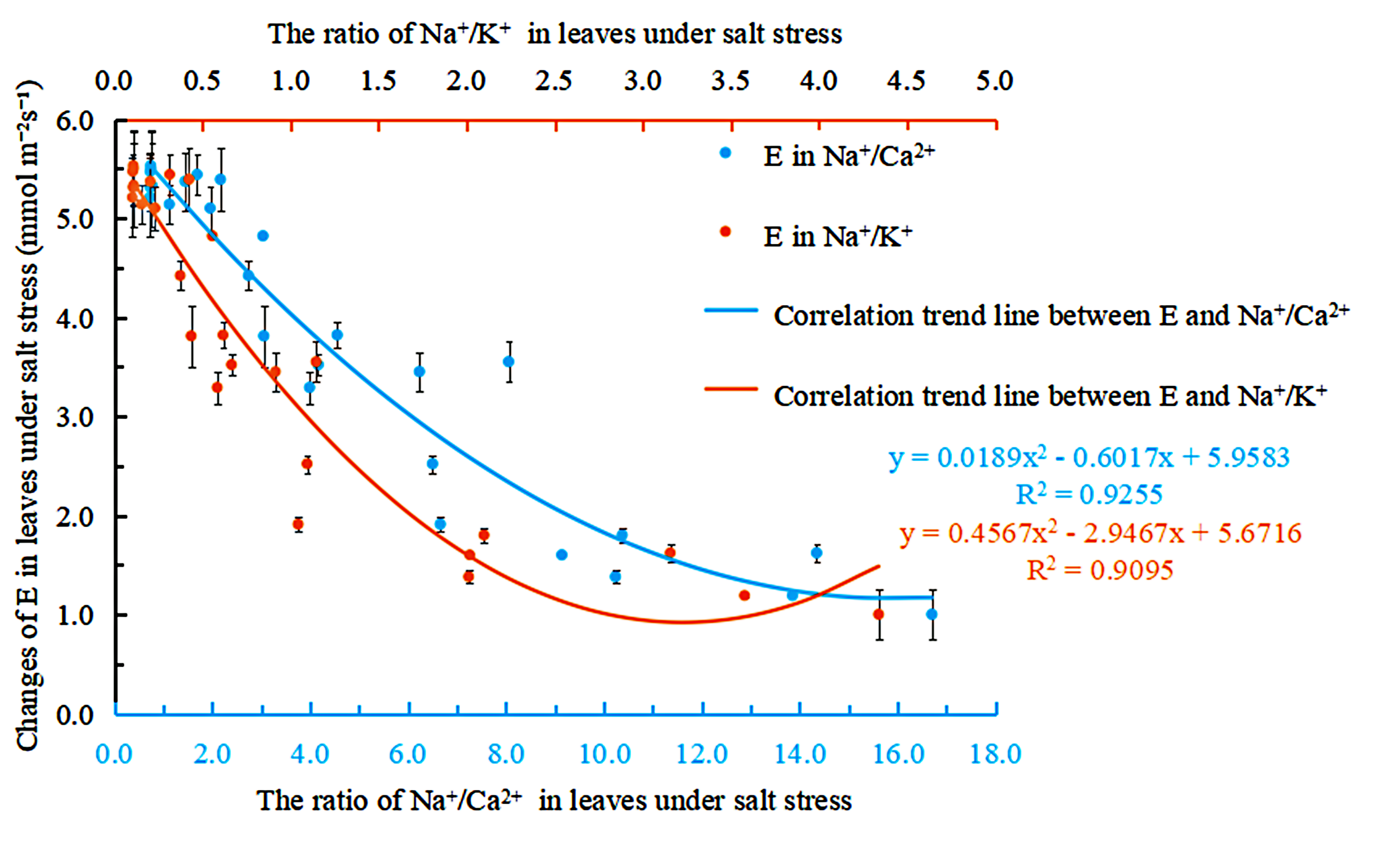

Supplement: Supplementary file 3 [file Data_Sheet_4.ZIP › Fig4b.tif]

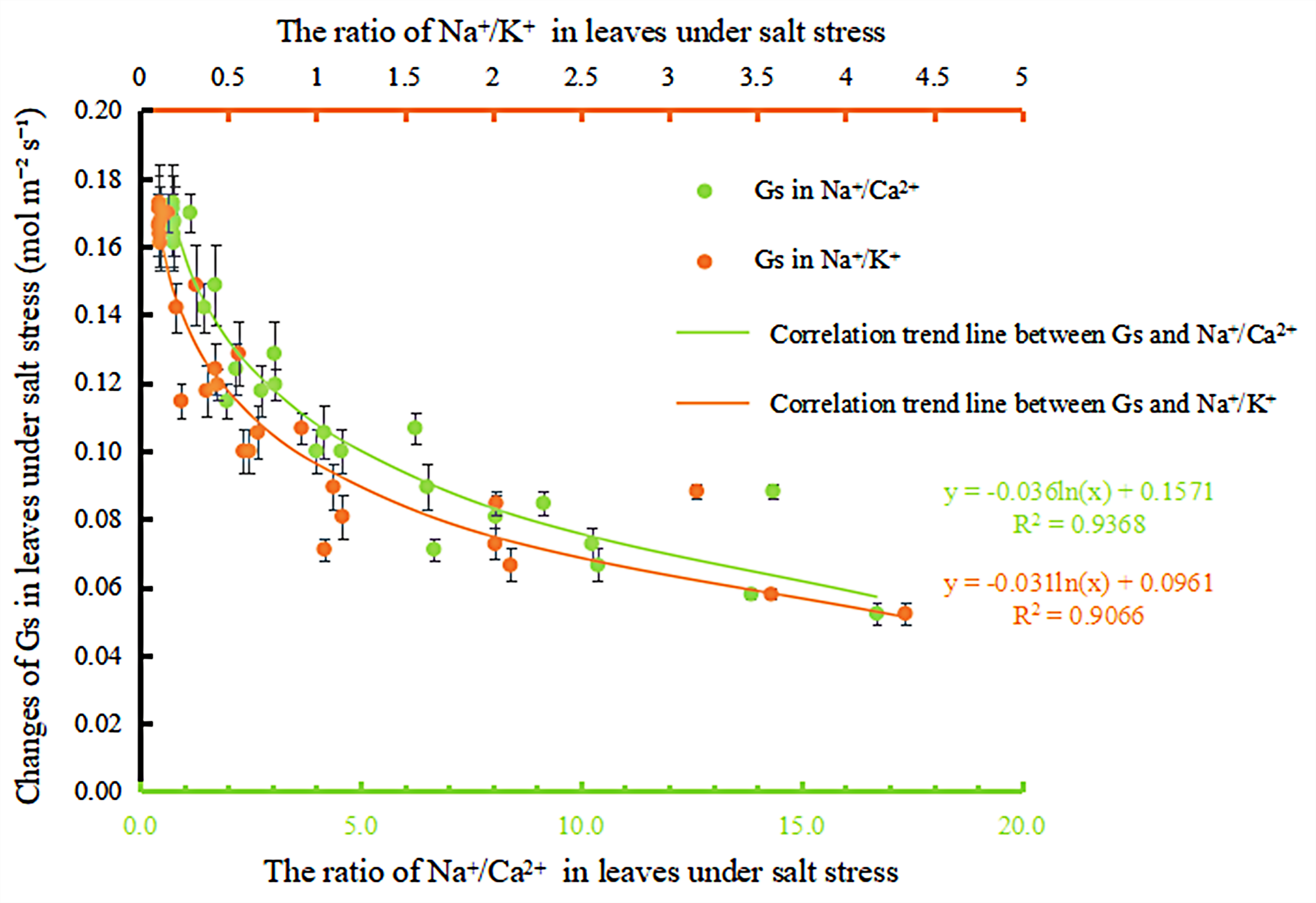

Supplement: Supplementary file 3 [file Data_Sheet_4.ZIP › Fig4c.tif]

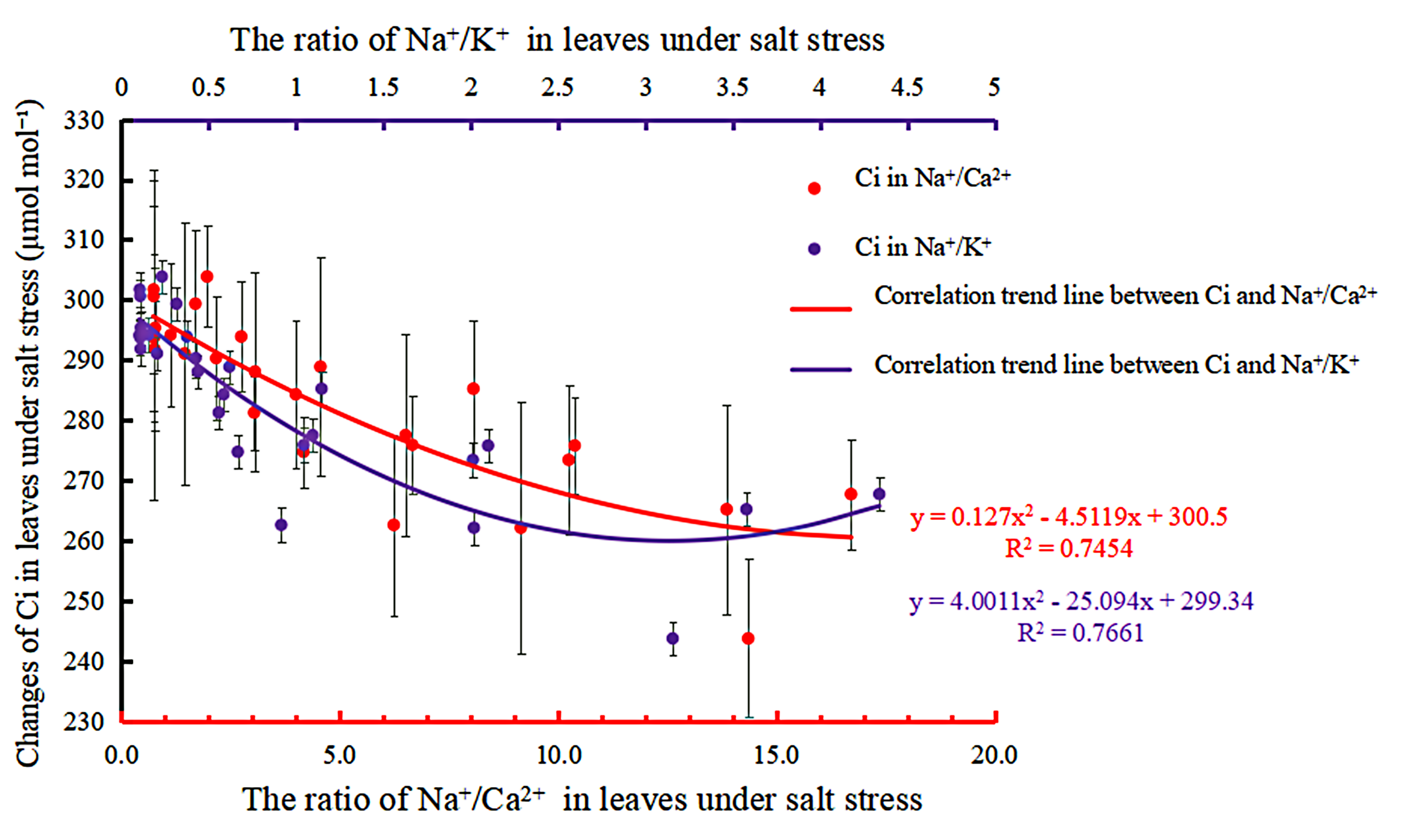

Supplement: Supplementary file 3 [file Data_Sheet_4.ZIP › Fig4d.tif]

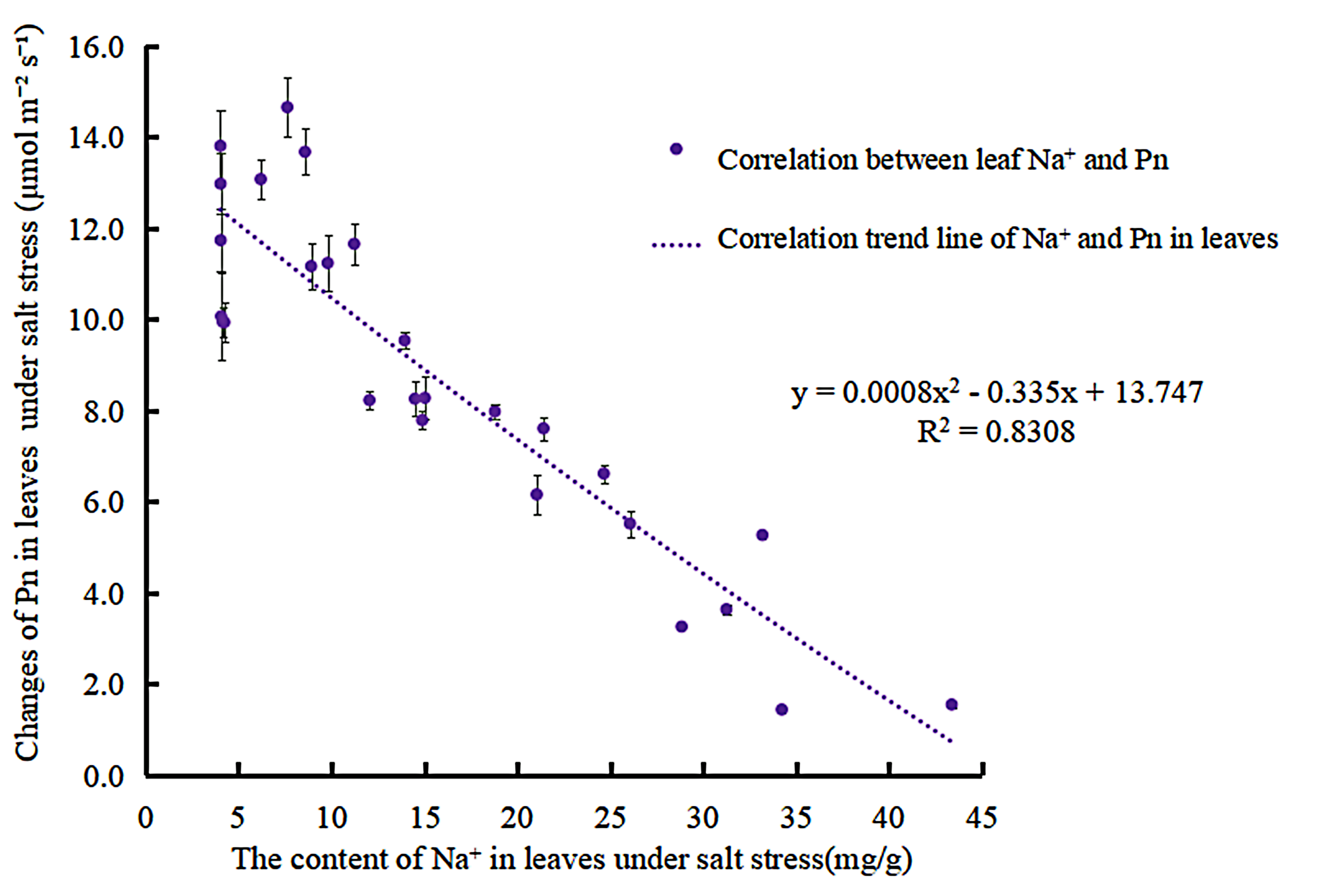

Supplement: Supplementary file 3 [file Data_Sheet_4.ZIP › Fig4e.tif]

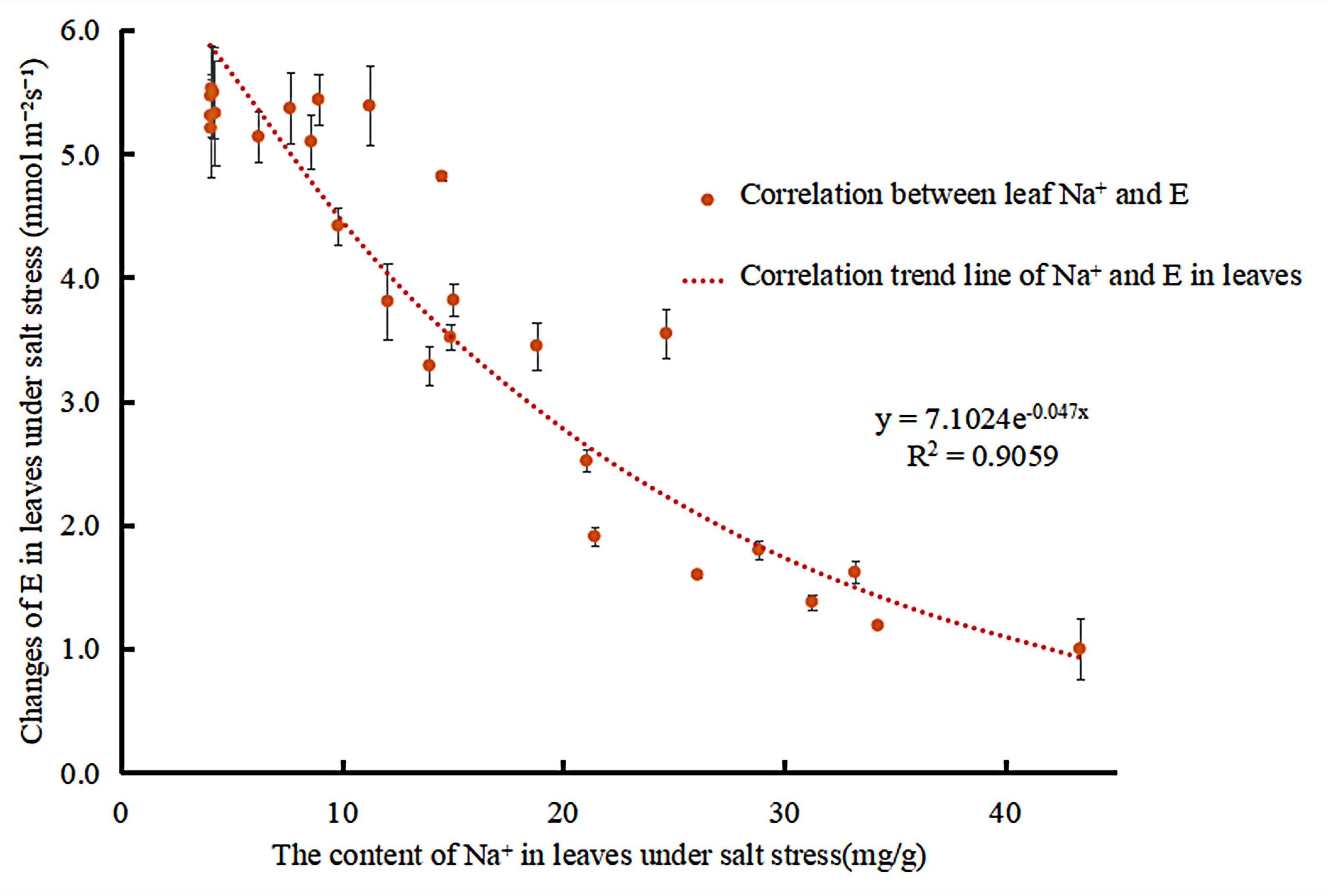

Supplement: Supplementary file 3 [file Data_Sheet_4.ZIP › Fig4f.tif]

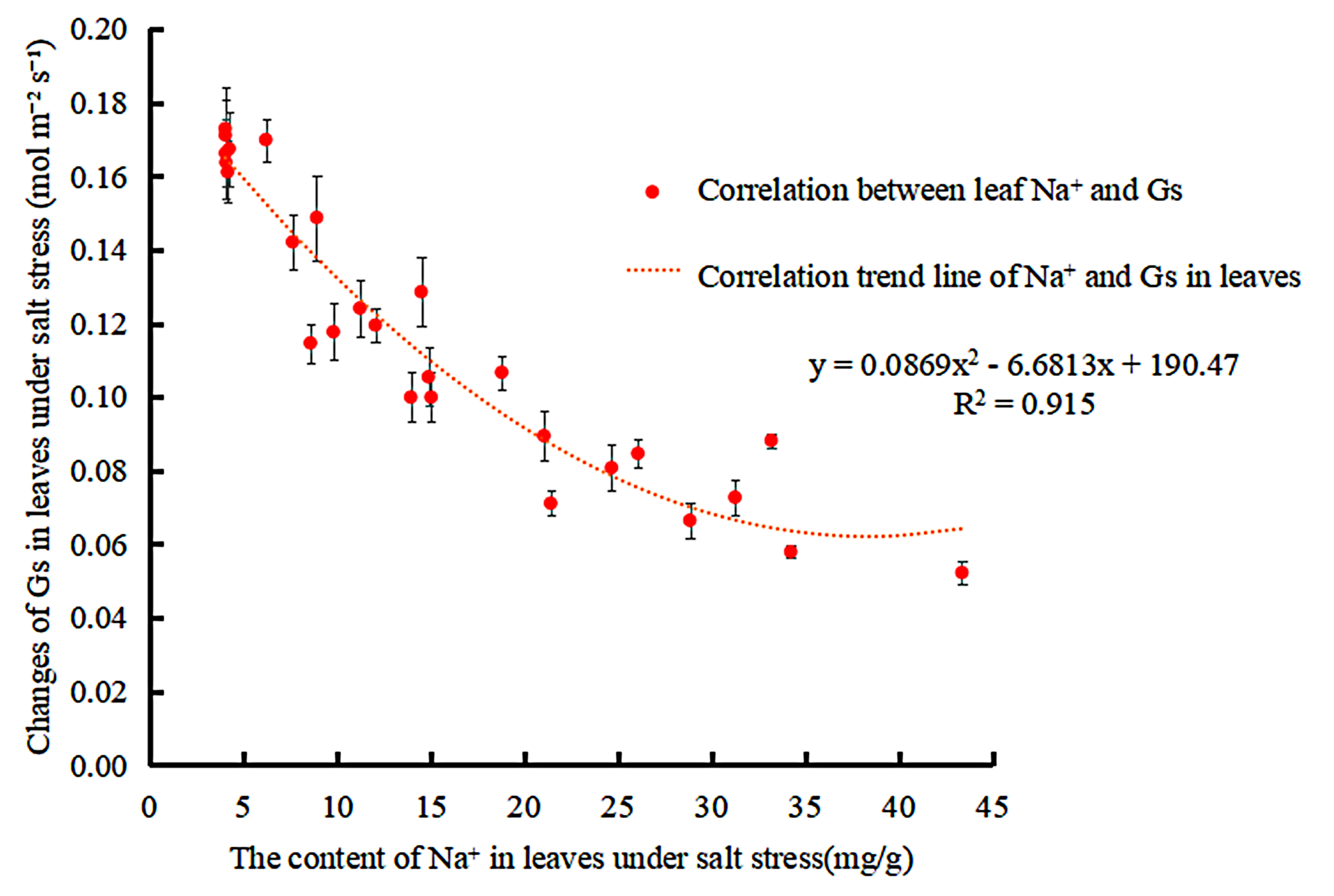

Supplement: Supplementary file 3 [file Data_Sheet_4.ZIP › Fig4g.tif]

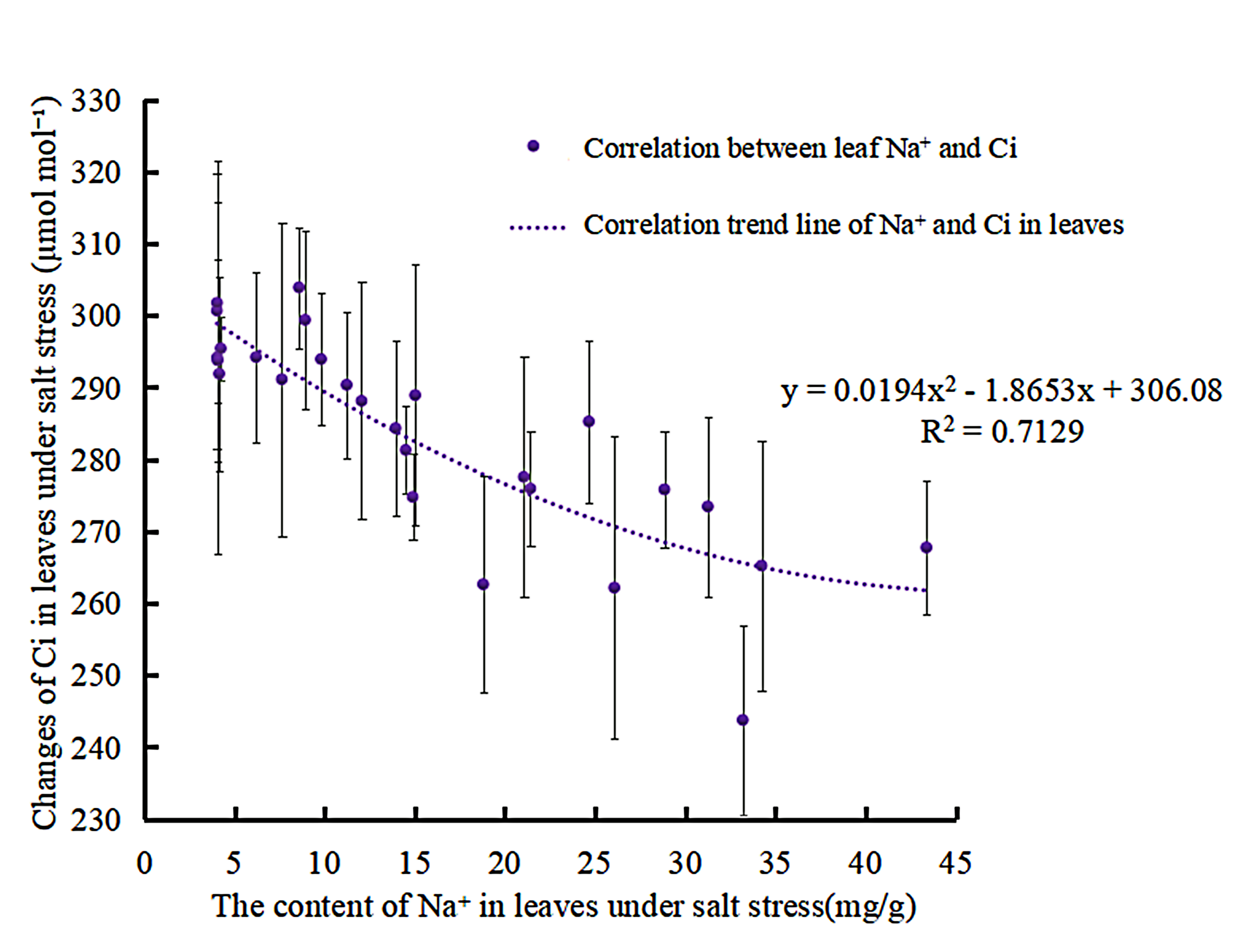

Supplement: Supplementary file 3 [file Data_Sheet_4.ZIP › Fig4h.tif]

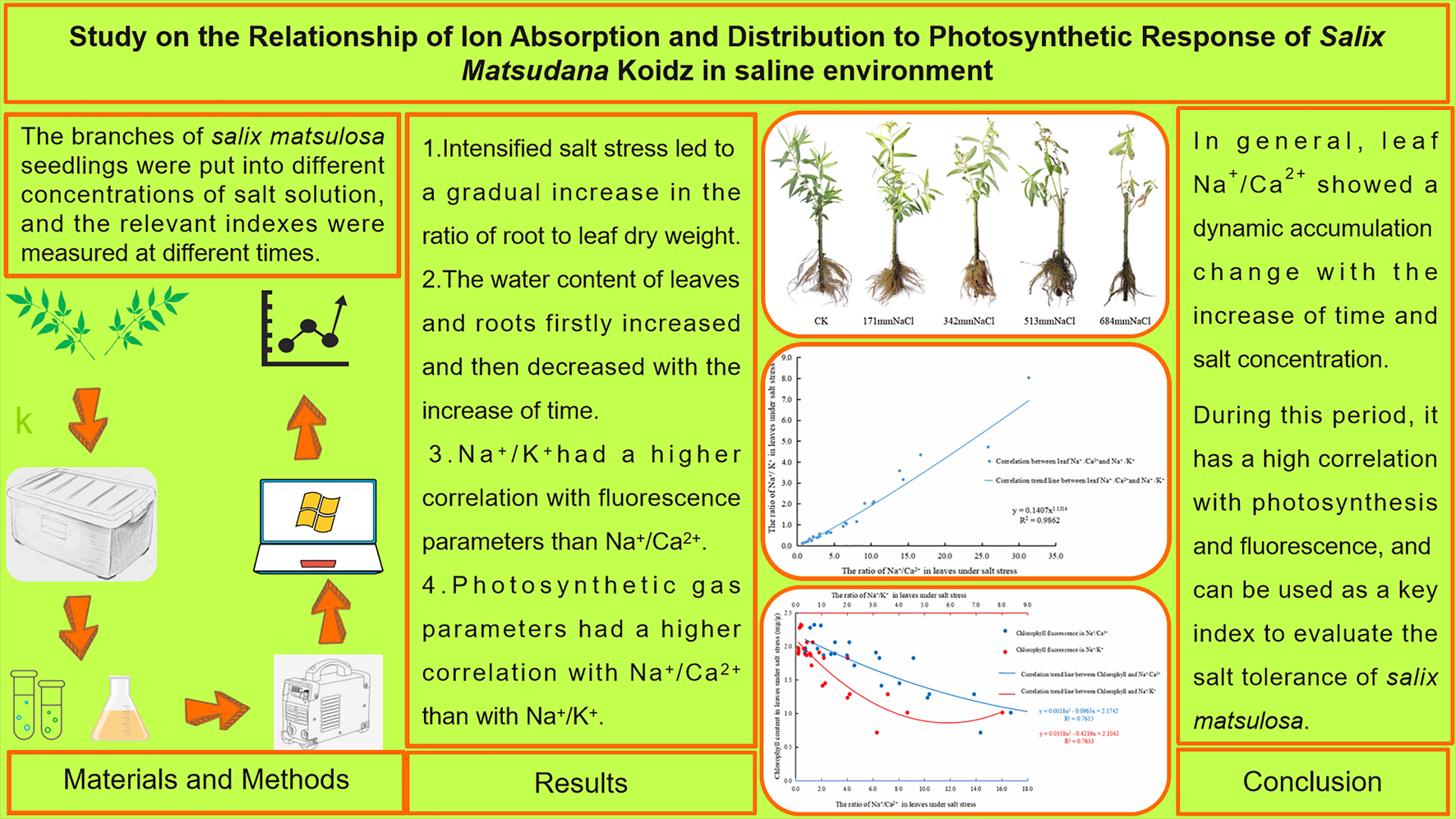

Supplement: Supplementary file 4 [file Image_1.TIF]
